# Pangenome analysis reveals genomic variations associated with domestication traits in broomcorn millet

**DOI:** 10.1038/s41588-023-01571-z

**Published:** 2023-11-30

**Authors:** Jinfeng Chen, Yang Liu, Minxuan Liu, Wenlei Guo, Yongqiang Wang, Qiang He, Weiyao Chen, Yi Liao, Wei Zhang, Yuanzhu Gao, Kongjun Dong, Ruiyu Ren, Tianyu Yang, Liyuan Zhang, Mingyu Qi, Zhiguang Li, Min Zhao, Haigang Wang, Junjie Wang, Zhijun Qiao, Haiquan Li, Yanmiao Jiang, Guoqing Liu, Xiaoqiang Song, Yarui Deng, Hai Li, Feng Yan, Yang Dong, Qingquan Li, Tao Li, Wenyao Yang, Jianghui Cui, Hongru Wang, Yongfeng Zhou, Xiaoming Zhang, Guanqing Jia, Ping Lu, Hui Zhi, Sha Tang, Xianmin Diao

**Affiliations:** 1 Institute of Crop Sciences, Chinese Academy of Agricultural Sciences, Beijing, China; 2 State Key Laboratory of Integrated Management of Pest Insects and Rodents, Institute of Zoology, Chinese Academy of Sciences, Beijing, China; 3 University of Chinese Academy of Sciences, Beijing, China; 4 Institute of Cotton, Hebei Academy of Agriculture and Forestry Sciences, Shijiazhuang, China; 5 College of Horticulture, South China Agricultural University, Guangzhou, China; 6 Crop Research Institute, Gansu Academy of Agricultural Sciences, Lanzhou, China; 7 Chifeng Academy of Agricultural and Animal Husbandry Sciences, Chifeng, China; 8 Center for Agricultural Genetic Resources Research, Shanxi Agricultural University, Taiyuan, China; 9 Institute of Millet Crops, Hebei Academy of Agriculture and Forestry Sciences, Shijiazhuang, China; 10 High Latitude Crops Institute to Shanxi Academy, Shanxi Agricultural University (Shanxi Academy of Agricultural Sciences), Datong, China; 11 Qiqihar Sub-academy of Heilongjiang Academy of Agricultural Sciences, Qiqihar, China; 12 Institute of Crop Sciences, Inner Mongolia Academy of Agricultural and Animal Husbandry Sciences, Hohhot, China; 13 College of Agronomy, Hebei Agricultural University, Baoding, China; 14 Shenzhen Branch, Guangdong Laboratory of Lingnan Modern Agriculture, Genome Analysis Laboratory of the Ministry of Agriculture and Rural Affairs, Agricultural Genomics Institute at Shenzhen, Chinese Academy of Agricultural Sciences, Shenzhen, China

**Keywords:** Plant breeding, Plant genetics

## Abstract

Broomcorn millet (*Panicum miliaceum* L.) is an orphan crop with the potential to improve cereal production and quality, and ensure food security. Here we present the genetic variations, population structure and diversity of a diverse worldwide collection of 516 broomcorn millet genomes. Population analysis indicated that the domesticated broomcorn millet originated from its wild progenitor in China. We then constructed a graph-based pangenome of broomcorn millet based on long-read de novo genome assemblies of 32 representative accessions. Our analysis revealed that the structural variations were highly associated with transposable elements, which influenced gene expression when located in the coding or regulatory regions. We also identified 139 loci associated with 31 key domestication and agronomic traits, including candidate genes and superior haplotypes, such as *LG1*, for panicle architecture. Thus, the study’s findings provide foundational resources for developing genomics-assisted breeding programs in broomcorn millet.

## Abstract (web summary)

Population analysis of 516 wild and domesticated broomcorn millet genomes and a graph-based pangenome based on de novo assemblies of 32 representative accessions identify genomic variations associated with domestication traits.

## Main

Climate change is a severe threat to global food security. Even though high-yielding, resource-efficient major crops have been developed, orphan crops provide an opportunity for climate-resilient agriculture and increased food supply^1,2^. However, despite exhibiting great nutritional diversity under low-input conditions, orphan crops are grown only locally by small and marginal farmers^3–5^. Therefore, studying these crops may help improve the nutritional diversity and environmental resilience of major crops.

Broomcorn millet (*Panicum miliaceum* L.) is an orphan crop mainly cultivated and consumed in the semiarid regions of Asia and Europe^6,7^. It was domesticated in Northern China around 10,000 years before present (BP)^8,9^ and was a staple food before the rise of rice and wheat in the area^8,10^. Broomcorn millet spread to Europe at approximately 3,600–4,000 years BP^11–13^. Broomcorn millet has potential as an alternative to major cereals, mainly due to its gluten-free nature, high protein content, and fast-growing and drought-tolerance characteristics^14,15^. However, despite the increasing demand and harvested areas in the United States^16^, only a few cultivars have been released to farmers^15,17^. Besides, the genomic diversity of broomcorn millet has not been extensively characterized^18–21^ and the genetic basis of its domestication remains to be explored.

Therefore, the present study aimed to analyze the genomes of a worldwide collection of broomcorn millet to identify its origin and explore the genetic basis of agronomic traits related to domestication. We used PacBio high-fidelity (HiFi) reads to generate de novo genome assemblies for 32 representative samples and built a graph-based pangenome to reveal the genomic variations in the broomcorn millet population. We surveyed 43 traits across multiple locations and analyzed the candidate genes associated with domestication and agronomic traits.

## Results

### Genome sequence, genetic diversity and population structure

**Fig. 1:**
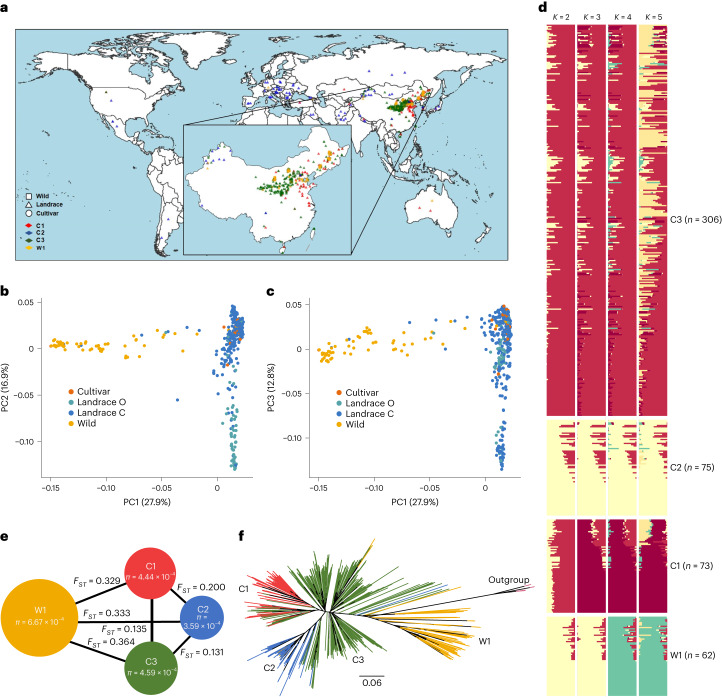
Geographical distribution and genomic diversity in broomcorn millet accessions in this study. **a**, Sample distribution of wild and cultivated broomcorn millet accessions. The squares represent the wild samples, the triangles represent the landraces and the circles represent the cultivars. Different colors represent the population structures inferred based on the resequencing dataset. The geographical map was obtained from Google Maps (https://www.google.com/maps) using the R package ggmap. **b**,**c**, PCA of 516 broomcorn millet accessions. PC1 (27.9%) clearly separates wild accessions from cultivated ones (landraces and cultivars); PC2 (16.9%) (**b**) separates most Chinese landraces (landrace C) from the European and Central Asian landraces (landrace O); and PC3 (12.8%) (**c**) divides the Chinese landraces into two subpopulations. **d**, Ancestral component analysis of broomcorn millet accessions with ADMIXTURE for *K* = 2**–**5. **e**, *π* and *F*_*ST*_ of broomcorn millet populations. **f**, A maximum likelihood phylogenetic tree of broomcorn millet accessions using switchgrass and foxtail millet (*Setaria italica*) as the outgroups. The colors represent populations identified with ADMIXTURE: 73 C1 (red branches); 75 C2 (blue branches); 306 C3 (green branches); and 62 W1 (brown branches). Source data

To explore broomcorn millet’s genetic diversity and population structure, we sequenced the genomes of 516 accessions, including 415 landraces, 38 cultivars and 63 wild accessions using 150-bp paired-end reads (Fig. 1a and Supplementary Table 1). This approach generated 7.6 terabytes of sequencing data (mapping rate = 99.4%; genomic coverage = 97.3%; depth of 17×) (Supplementary Table 2). After mapping these reads to the Longmi4 reference genome^6^, we identified 1,890,542 high-quality SNPs and 168,878 insertions and deletions (indels; 1–50 bp). The SNPs were denser in the chromosomal arms than in the pericentromeric regions (Supplementary Fig. 1a), probably due to low selection-associated nucleotide diversity (*π*) in the low-recombination regions^22,23^. Additionally, the linkage disequilibrium (LD) among SNPs rapidly decreased at 100–200 kb (Supplementary Fig. 2).

To determine population structure, we used principal component analysis (PCA) on 12,816 fourfold degenerate (4DTv) sites and identified the first three principal components (PCs), which accounted for 57.6% of the data variance (Fig. 1b,c). We then used ADMIXTURE^24^, STRUCTURE^25^, fastSTRUCTURE^26^ and discriminant analysis of PC (DAPC)^27^ to perform ancestral component analyses on 57,930 pruned high-quality SNPs, optimizing for the number of population clusters. The results demonstrated that the investigated samples could be divided into four clusters: one wild cluster W1; and three cultivated clusters, that is, C1, C2 and C3 (Fig. 1d and Supplementary Figs. 3–5). The largest cluster, C3, contained cultivated accessions from Northwest China, the primary area for broomcorn millet farming. The C1 cluster consisted of cultivated accessions from Northeast and East China, and the C2 cluster included cultivated accessions from European and Central Asian countries (Fig. 1a). These findings indicate that the population structure of broomcorn millet is largely correlated with geographical location.

Further analysis revealed that the *π* of the cultivated and wild accessions of broomcorn millet (*π* = 0.00042 and *π* = 0.00067, respectively; Fig. 1e) were lower than those of rice (*π* = 0.0024 and *π* = 0.0030, respectively) and soybean (*π* = 0.0012 and *π* = 0.0029, respectively)^28,29^. The cultivated accessions retained 62.6% of the *π* in their wild relatives. In the phylogenetic tree, the wild population formed a cluster distinct from the three cultivated populations (Fig. 1f). The C3 cluster exhibited higher complexity and was closely related to the wild population, suggesting that the C3 cluster represents the gene pool domesticated from wild accessions. Additionally, a few accessions from Xinjiang and Gansu within the C3 cluster formed the basal lineage or were within the C2 branches (Fig. 1f), suggesting that the European and Central Asian accessions may have originated from Northwest Chinese accessions^30^. We also identified gene flow between cultivated and wild populations (Supplementary Fig. 6). In conclusion, these results suggest that broomcorn millet was domesticated in Northern China and its cultivation subsequently spread to the West from Northwest China.

### Pangenome analysis of broomcorn millet

**Fig. 2:**
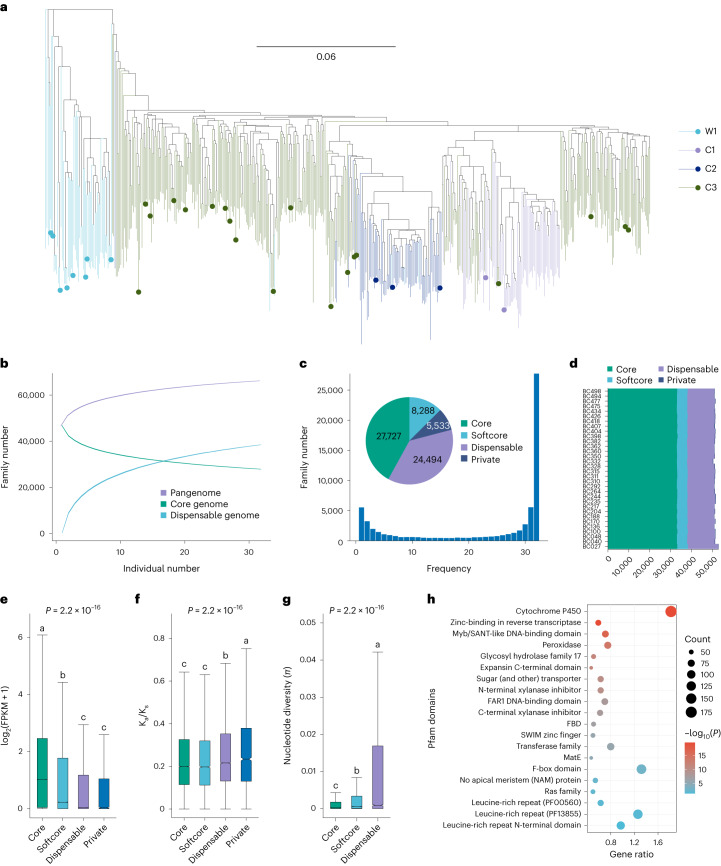
Pangenome analysis of 32 broomcorn millet accessions. **a**, Phylogeny of 516 accessions. The phylogenetic tree was built with SNP data using IQ-TREE. The 32 accessions selected for pangenome analysis are indicated by colored circles in the phylogenetic tree. **b**, Number of pan, core and dispensable gene families. Each number was randomly sampled 100 times. **c**, Composition of the pangenome. The histogram shows the frequency of gene families and the pie chart depicts the proportions of core, softcore, dispensable and private gene families. **d**, Compositions of the individual genomes. Each row represents an accession. **e**, Expressions of core, softcore, dispensable and private genes across 32 accessions. The numbers of core, softcore, dispensable and private genes in the box plot are 1,061,505, 158,766, 417,550 and 6,249, respectively. **f**, Comparison of K_a_/K_s_ values of core, softcore, dispensable and private genes. The numbers of gene pairs in the core, softcore, dispensable and private categories in the box plot are 26,024, 7,214, 15,889 and 2,446, respectively. **g**, Comparison of *π* of core, softcore and dispensable genes. The numbers of gene families in core, softcore and dispensable in the box plot are 27,609, 8,193 and 24,213, respectively. In **e**,**f**,**g**, the edges and centerlines of the boxes represent the interquartile range (IQR) and medians, with the whiskers extending to the most extreme points (1.5× IQR). Significance was tested using a Kruskal–Wallis test; multiple comparisons were analyzed using a Nemenyi test. The different lowercase letters above the box plots represent significant differences (*P* ≤ 0.05). **h**, Top 20 Pfam domains enriched in dispensable genes. The enrichment test was performed based on a hypergeometric distribution. *P* values were false discovery rate (FDR)-adjusted. Source data

We selected 32 accessions, including 24 cultivated and eight wild ones, representing all major lineages to construct the broomcorn millet pangenome (Fig. 2a). PacBio HiFi reads (35×) were assembled with hifiasm^31^ (Supplementary Table 3) and resulted in contigs with N50 ranging from 5.16 to 27.25 Mb (Supplementary Table 3). Finally, we generated 32 chromosome-scale assemblies by anchoring the contigs with Longmi4 (Supplementary Tables 3–5 and Supplementary Fig. 7). Quality assessment revealed 96.0% Benchmarking Universal Single-Copy Orthologs (BUSCO) completeness^32^ and a 13.5 long terminal repeat (LTR) assembly index (LAI) score^33^ (Supplementary Table 3), suggesting that the genome sequences are of high quality and are highly contiguous.

We further identified an average of 58.1% repetitive sequences per genome (Supplementary Table 6), with larger genomes exhibiting more transposable elements (TEs) (*R* = 0.92, *P* = 8.11 × 10^−14^; Supplementary Fig. 8). Then, using MAKER2 (ref. ^34^), we annotated an average of 59,332 protein-coding genes per genome, with 95.4% BUSCO completeness (Supplementary Table 7). We identified 27,727 core, 8,288 softcore, 24,494 dispensable and 5,533 private gene families across the 32 genomes (Fig. 2b–d). Core genes showed higher expressions but lower *π*, and nonsynonymous to synonymous substitution ratios (K_a_/K_s_), than dispensable and private genes (Fig. 2e–g). Moreover, core genes were enriched with domains related to the basic biological processes, such as RNA recognition motifs (*P* = 1.67 × 10^−8^) and helicases (*P* = 1.05 × 10^−5^) (Supplementary Table 8). In contrast, dispensable genes were enriched with domains related to enzyme activity and stress resistance, such as leucine-rich repeats (*P* ≤ 0.05) (Fig. 2h and Supplementary Table 9). Private genes accounted for 8.4% of gene families in broomcorn millet; however, they contained only 0.4% of protein-coding genes in each accession (Fig. 2c,d). These private gene families may represent lineage-specific genes, and their proportions vary among different species (Supplementary Table 10)^35–37^. Taken together, these results suggest that the dispensable genome of broomcorn millet is enriched with stress resistance genes^35,37^ that may contribute substantially to their genomic diversity.

### Structural variations in broomcorn millet

**Fig. 3:**
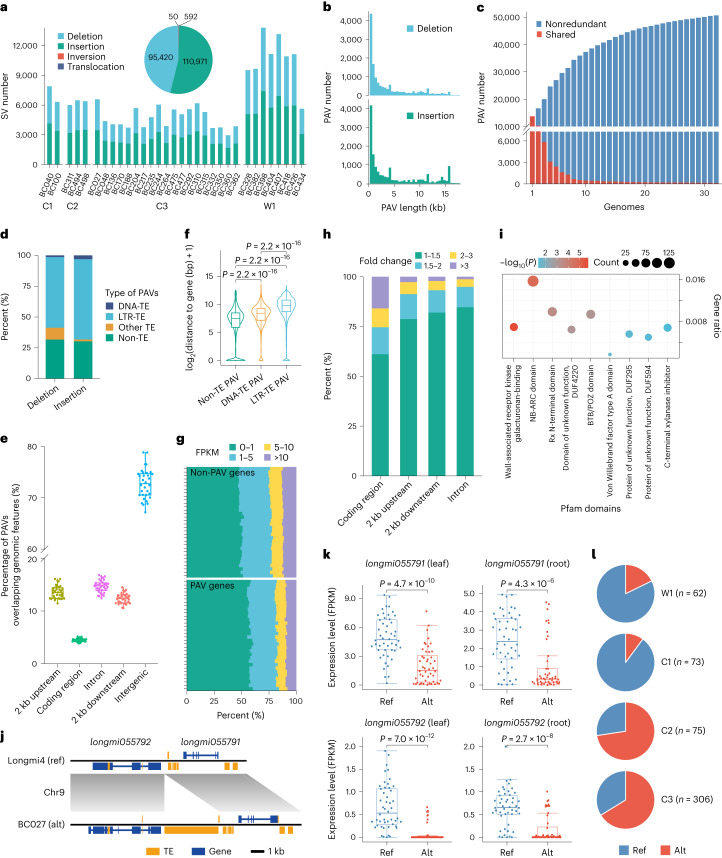
SVs in the genomes of 32 broomcorn millet accessions. **a**, Number of SVs (deletions, insertions, inversions and translocations) in each accession. The proportions of nonredundant SVs are shown in the pie chart. **b**, Histogram showing the length of deletions and insertions (PAVs). **c**, Number of shared and nonredundant PAVs. The accessions are displayed in the following order: BC407, BC404, BC426, BC418, BC382, BC328, BC040, BC027, BC494, BC100, BC498, BC310, BC244, BC311, BC204, BC434, BC292, BC475, BC315, BC477, BC235, BC048, BC136, BC170, BC264, BC362, BC217, BC350, BC188, BC332 and BC360. **d**, Percentage of TE-derived and non-TE PAVs in the broomcorn millet genome. **e**, Percentage of PAVs in the various genomic features. The number of samples in each feature is 32. Each dot represents one accession. **f**, Distance of PAVs to their closest protein-coding genes. The numbers of genes in non-TE PAVs, LTR-TE PAVs and DNA-TEs are 16,001, 30,499 and 1,114, respectively. A two-sided Wilcoxon test was used to determine the significant levels. **g**, Comparison of gene expressions between PAV and non-PAV genes in the leaf tissues of 32 accessions. **h**, Fold change in gene expression between accessions with and without PAVs in the coding region, intron, 2 kb upstream of the start codon and 2 kb downstream of the stop codon of protein-coding genes. The fold change represents the expression change between genes in accessions with or without PAVs. **i**, Pfam enrichment of PAV genes. The enrichment test was performed based on a hypergeometric distribution. *P* values were FDR-adjusted. **j**, A 4.6-kb PAV associated with the disease resistance genes *longmi055791* and *longmi055792*. **k**, Expression of *longmi055791* and *longmi055792* in accessions with (Alt) or without PAV (Ref). A two-sided Wilcoxon test was used to determine the significant levels. The numbers of samples are 16 for Alt and 16 for Ref accessions; each accession consists of three biologically independent plants. **l**, Distribution of the 4.6-kb PAV in the broomcorn millet populations. The number in parentheses represents the number of individuals with unambiguous PAV genotypes in each population. In **e**,**f**,**k**, the edges and centerlines of the boxes represent the IQR and medians, with the whiskers extending to the most extreme points (1.5× IQR). Source data

To further explore genomic diversity in broomcorn millet, we used an assembly-based method to identify the structural variations (SVs) (>50 bp) in the genomes. We identified 207,033 SVs (Supplementary Table 11 and Fig. 3a) with an accuracy of 87.1% (135 of 155) (Supplementary Tables 12 and 13 and Supplementary Figs. 9 and 10). Subsequently, we merged the SVs from all accessions into 50,689 nonredundant SVs and analyzed the 50,515 presence or absence variants (PAVs) (26,195 deletions, 24,320 insertions) in the rest of the study (Fig. 3b,c and Supplementary Fig. 11). We found that 59.4% (29,998 of 50,515) of PAVs were present in only one or two accessions (Supplementary Fig. 12). This is consistent with low-frequency PAVs in rice and soybean^35,37^, suggesting that they represent the newly emerged or deleterious mutations subjected to purifying selection^38^. Besides, unlike SNPs, PAVs displayed no decrease in density in the pericentromeric regions (Supplementary Fig. 1b). Finally, we constructed a graph-based pangenome using 50,515 PAVs and Longmi4 with the vg toolkit^39^ and genotyped the PAVs across 516 accessions (Supplementary Figs. 13 and 14). This graph-based pangenome provides a foundation for analyzing the effects of PAVs on the phenotypic variations in broomcorn millet.

We classified the PAVs that overlapped 90% with TEs as TE-derived PAVs; the remaining PAVs were classified as non-TE PAVs. TE-derived PAVs constituted the majority (68.3%) of all PAVs (Fig. 3d and Supplementary Fig. 15a–c). We further annotated PAVs based on their location relative to the protein-coding genes and found that 32.9% overlapped with the genic regions (Fig. 3e and Supplementary Table 11). Of all non-TE PAVs, 51.7% were located in the genic regions (8,157 genes), while only 13.2% of the TE-derived PAVs were associated with the genic regions (4,458 genes). In addition, the DNA-TE PAVs were closer to the genic regions than the LTR-TE PAVs (10.5 kb versus 35.3 kb; Fig. 3f). To understand how PAVs affect gene function, we compared the expression levels of genes with PAV-affected regions (PAV genes) and those without PAV (non-PAV genes) in each accession, explicitly focusing on genes that shared synteny between broomcorn millet and its diploid relative *Panicum hallii*. We found that the expression levels of PAV genes were significantly lower than those of non-PAV genes (5.03 versus 6.42, *P* = 2.2 × 10^−16^ in leaves and 4.55 versus 6.45, *P* = 2.2 × 10^−16^ in roots) (Supplementary Fig. 16a). Besides, PAV genes had more silenced genes (fragments per kilobase of transcript per million mapped reads (FPKM) < 1) than non-PAV genes (*P* = 2.2 × 10^−16^ in leaves; *P* = 2.2 × 10^−16^ in roots; Fig. 3g and Supplementary Fig. 16b,c), indicating that PAVs were associated with reduced gene expression in both leaves and roots. Additionally, TE-derived PAVs located in the coding regions and upstream of genes were more likely to affect gene expression than those located in introns and downstream of genes (Fig. 3h, Supplementary Fig. 17 and Supplementary Table 14). Thus, our findings suggest that PAVs influence gene expression by altering the coding and *cis*-regulatory regions.

Furthermore, we identified 648 PAV genes with significantly altered expression levels in the leaves and roots (Supplementary Fig. 18). These differentially expressed PAV genes were enriched with resistance-related domains, such as NB-ARC (*P* = 0.002) and Rx N-terminal domains (*P* = 0.007), which were also PAV gene-enriched Pfam domains (Fig. 3i). We found that resistance genes were located in repeat-rich regions and had a higher frequency of surrounding PAVs than the genome average (Supplementary Fig. 19), suggesting that PAVs are associated with the evolution of resistance genes in broomcorn millet. For instance, in BC027, we found a 4.6-kb insertion between two resistance genes, *longmi055791*, encoding a homologous protein of ENHANCED DISEASE RESISTANCE 2 (ref. ^40^), and *longmi055792*, encoding an NBS-LRR gene (Fig. 3j). The insertion is associated with decreased expression of both genes (Fig. 3k) and its allele frequency is higher in C2 (73.3%) and C3 (66.0%) populations than in W1 (17.8%) (Fig. 3l). These results suggest that this mutation might have facilitated the adaptation of broomcorn millet to Northwest China (C3) and Europe (C2).

### Artificial selection during broomcorn millet domestication

**Fig. 4:**
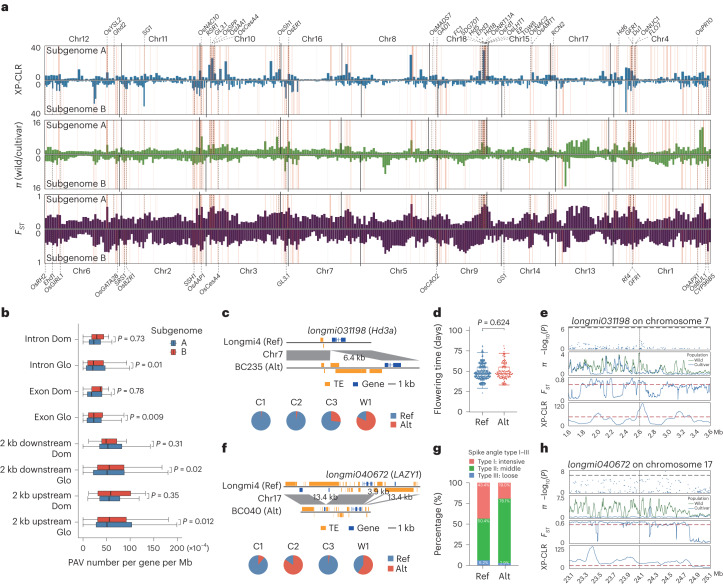
Genomic regions under artificial selection during broomcorn millet domestication. **a**, Genome-wide identification of artificial selection regions in domesticated broomcorn millet. From top to bottom, the blue, green and purple peaks represent signals of XP-CLR, *π* and *F*_*ST,*_ respectively, in the 20-kb genomic window. Chromosomes of subgenome A were plotted upward in the following order: Chr12, Chr11, Chr10, Chr16, Chr8, Chr18, Chr15, Chr17, Chr4; those of subgenome B were plotted downward in the following order: Chr6, Chr2, Chr3, Chr7, Chr5, Chr9, Chr14, Chr13, Chr1. The red lines represent artificial selection regions identified in this study. Locations of known genes in rice are indicated by their gene symbols. **b**, PAV density in genic features. The densities of PAVs in the artificial selection regions (Dom, *n* = 59) and the whole-genome regions (Glo, *n* = 772) are shown. Significant levels were determined using a two-sided Student’s *t*-test. **c**, Comparison of BC235 and Longmi4 sequences with a 6.4-kb insertion at the promoter of *longmi031198* (ortholog of *Hd3a*). The pie charts show the PAV frequencies in the C1, C2, C3 and W1 populations. The allele frequency for the Hap without the insertion is 19.4% in the wild versus 98.6% in C1, 97.3% in C2 and 72.9% in C3. **d**, Flowering time in accessions with the Ref and Alt alleles of *longmi031198*. The number of samples in the Ref and Alt alleles are 375 and 136, respectively. The significance level was determined using a two-sided Wilcoxon test. **e**, GWAS signal, *π*, *F*_*ST*_ and XP-CLR in the 1-Mb region of *longmi031198*. The black lines represent the SNP-GWAS significance threshold, which was set at 0.05/total number of SNPs (*P* = 2.64 × 10^−8^ or −log_10_(*P*) = 7.58). **f**, Comparisons of BC040 and Longmi4 sequences with three closely located deletions (13.4, 3.9 and 13.4 kb) at the *LAZY1* locus. The pie charts show the PAV frequencies in the C1, C2, C3 and W1 populations. **g**, Percentage of angle types (I–III) between the panicle branches and spindle of accessions with Ref and Alt alleles of *longmi040672*. **h**, GWAS signal, *π*, *F*_*ST*_ and XP-CLR in the 1-Mb region of *longmi040672.* The black lines represent the SNP-GWAS significance threshold, which was set at 0.05/total number of SNPs (*P* = 2.64 × 10^−8^ or −log_10_(*P*) = 7.58). In **b**,**d**, the edges and the centerlines of the boxes represent the IQR and medians, with the whiskers extending to the most extreme points (1.5× IQR). Source data

We used a complementary method by integrating a cross-population composite likelihood ratio (XP-CLR)^41^, *π*_wild_/*π*_cultivar_ ratio and fixation index (*F*_*ST*_) to detect signals of artificial selection. We compared all cultivated accessions to their wild counterparts and identified 524 genomic regions as targets of artificial selection, covering 30.2 Mb sequences and 3,910 protein-coding genes (Fig. 4a and Supplementary Table 15). These regions overlapped with several known genes linked to domestication and adaptation traits, such as grain yield (*GL3.1*, *SG1* and *GS1*) and flowering time (*Ghd2, Ehd1* and *Hd5*) (Fig. 4a). We found that three cultivated populations (C1, C2 and C3) exhibited distinct selection patterns compared to the wild population (Supplementary Fig. 20). The genes overlapping with the selective regions were enriched in functions related to resistance, such as pathogenesis-related protein 1 and MYC2 in C1, and abscisic acid biosynthesis and calcium-dependent protein kinase in C3 (*P* < 0.05; Supplementary Table 16). These results suggest that each cultivated population developed resistance mechanisms against pathogens, herbivorous insects or drought to adapt to the local environment.

Broomcorn millet is an allopolyploid species containing two subgenomes (A and B)^42^. We found that the selective regions were more abundant and contained more protein-coding genes in subgenome A than in subgenome B (287 versus 237 for selective regions and 2,387 versus 1,523 for selected genes; Supplementary Table 15). We also observed that the protein-coding genes in subgenome B had more PAVs than those in subgenome A (*t*-test, *P* = 0.012 for 2 kb upstream, *P* = 0.020 for 2 kb downstream; *P* = 0.009 for exon and *P* = 0.010 for intron) (Fig. 4b) and showed more differences in expression across tissues (fold change ≥ 1.5; *z*-test *P* = 5.95 × 10^−3^ in leaves and *P* = 2.56 × 10^−3^ in roots; Supplementary Fig. 21). Furthermore, we analyzed gene loss and pseudogenization events associated with PAVs to understand how PAVs affect gene fractionation in the allopolyploid genome. We identified 1,321 genes deleted or pseudogenized by PAVs (Supplementary Table 17 and Supplementary Fig. 22a,b) and found that subgenome B experienced more gene loss than subgenome A (Supplementary Fig. 22c). This result is consistent with the finding that the TE-rich subgenome B underwent biased gene loss^42^, indicating that PAVs facilitated gene fractionation. We also identified 242 gene losses, which were present at a lower frequency in the wild population than in the cultivated population (Supplementary Fig. 22d,e). These results suggest that the ongoing rediploidization in the broomcorn millet genome may have affected gene function, contributing to its domestication.

To better understand how genomic variations affect the function of genes during domestication, we identified 1,099 PAVs in 225 regions under selection, including 39.9% (438 of 1,099) TE-derived PAVs. Among these, 503 PAVs overlapped with the genic regions. We also found 5,663 PAVs with significantly altered allele frequency between wild and cultivated populations during domestication (Supplementary Fig. 15d,e). Integrating PAV-affected genes from the above analyses, we identified 4,930 genes putatively associated with broomcorn millet domestication (Supplementary Table 18). A 6.4-kb TE insertion was identified in the upstream region of *longmi031198* (Fig. 4c), an ortholog of the rice florigen gene *Hd3a*^43^. This mutation (Alt) showed no significant association with the flowering phenotype (Fig. 4d); however, the cultivated population showed an increased allele frequency for the haplotype (Hap) without the insertion (Ref) (Fig. 4c,e). Three closely located deletions (13.4, 3.9 and 13.4 kb) around *longmi040672*, an ortholog of *LAZY1* (ref. ^44^), were also identified (Fig. 4f). The Hap with the deletion (Alt) was associated with a larger angle between the spike and main stem (Fig. 4g) and was selected against during domestication (Fig. 4h and Supplementary Fig. 23). These results suggest that PAVs, especially TE-derived PAVs, may have had an important role in the domestication of broomcorn millet.

### Genomic variations associated with domestication

**Fig. 5:**
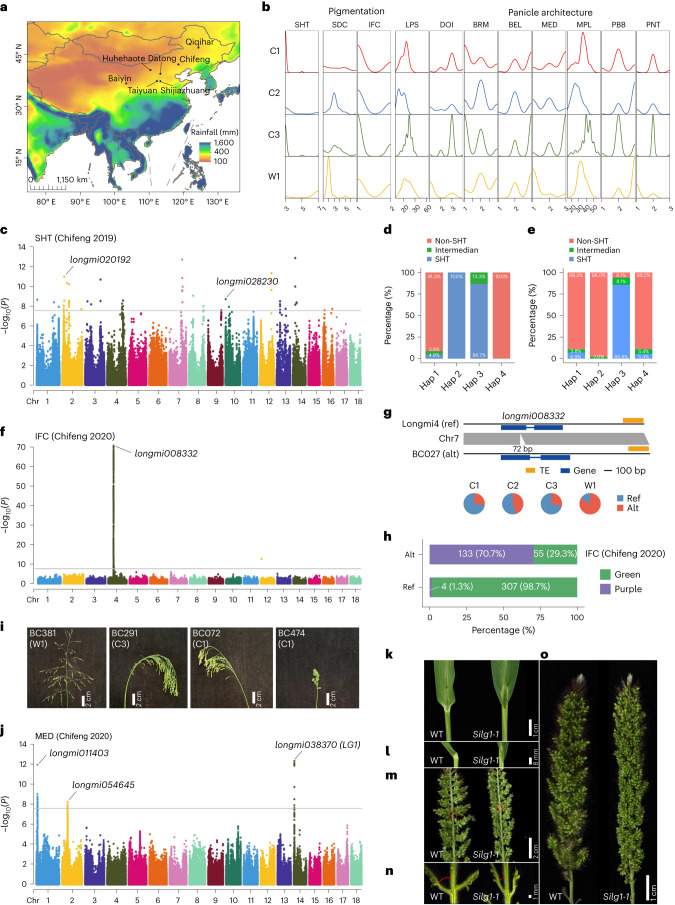
GWAS of genomic variations associated with domestication and agronomically important traits in broomcorn millet. **a**, Location of seven sites used for phenotype evaluation. The colors represent the yearly rainfall. The geographical map was obtained from ArcGIS (https://www.arcgis.com/index.html). The yearly rainfall data were obtained from WorldClim (https://www.worldclim.org/). **b**, Distribution of SHT, seed and inflorescence (colors), and panicle architecture traits in broomcorn millet. SHT, SDC, IFC, length of panicle stem (LPS), DOI, the branch of the ear of grain and main shaft drift angle (BRM), the branch of ear length (BEL), MED, main panicle length (MPL), projection on branch base (PBB) and PNT are shown. Details of the traits are described in Supplementary Table 19. **c**, Manhattan plot of the GWAS of seed SHT based on the Chifeng 2019 phenotype. **d**,**e**, SHT phenotype of four Haps of *longmi020192* (**d**) and *longmi028230* (**e**). In **d**, the numbers of accessions with the Hap 1, 2, 3 and 4 Haps were 446, 26, 15 and 12, respectively. In **e**, the numbers of accessions with the Hap 1, 2, 3 and 4 Haps were 318, 99, 49 and 37, respectively. **f**, Manhattan plot of GWAS of IFC based on the phenotype collected from Chifeng in 2020. **g**, Comparisons of BC027 and Longmi4 sequences with a 72-bp insertion at the *longmi008332* locus. **h**, Distribution of IFC in Ref and Alt alleles. **i**, Panicles of the representative accessions of wild and cultivated broomcorn millet. **j**, Manhattan plot of the GWAS of MED based on the phenotype collected from Chifeng in 2020. **k**–**o**, Phenotypic analyses of ligule (**k**,**l**) and panicle traits (**m**–**o**) of the *lg-1* mutant and WT plants. The horizontal lines in **c**,**f**,**j** depict the significance threshold (*P* = 2.64 × 10^−8^ or −log_10_(*P*) = 7.58), which was set at 0.05/*n* (*n* represents the total number of SNPs). Source data

Furthermore, to link genomic and phenotypic variations in broomcorn millet, we measured 43 traits for 516 accessions at seven locations over 2 years (Fig. 5a, Supplementary Fig. 24 and Supplementary Table 19) and conducted genome-wide association studies (GWAS) based on 1,890,542 SNPs and 19,492 PAVs. The SNP-GWAS identified 139 loci significantly associated with 31 traits, including many agronomically important traits, such as seed dimension and plant architecture, as well as those associated with domestication syndrome, such as seed shattering (SHT) and panicle type (PNT) (Supplementary Table 20). Meanwhile, the PAV-GWAS revealed 70 PAVs associated with 17 traits (Supplementary Table 21). The association signals identified by the PAV-GWAS analysis were consistent with those identified by the SNP-GWAS. The PAV-GWAS only identified a few signals compared to those identified by the SNP-GWAS (Supplementary Fig. 25). However, PAV-GWAS has the potential to identify causal mutations underlying phenotypic variations (Supplementary Fig. 25), making it a complement to the SNP-GWAS in identifying mutations associated with phenotypes^45^. We provide details for the following key traits: seed SHT; inflorescence and seed color; and panicle architecture. They represent domestication syndrome and are crucial for broomcorn millet improvement (Fig. 5b).

#### Seed SHT

Loss of seed SHT was a crucial step in cereal crop domestication^46^. Phenotypic analysis of the present study indicated that cultivated populations had lower SHT levels than wild accessions (Fig. 5b), suggesting intense selection for the non-SHT phenotype during domestication. To uncover the genetic variations associated with the non-SHT phenotype in cultivated accessions, we examined the homologous genes of 15 known SHT genes of cereal crops (Supplementary Table 22). We found that *longmi009317*, the ortholog of *OsSh1*, which controls seed SHT in rice and sorghum^47^, and a related homolog, *longmi003952*, underwent gene loss or pseudogenization in broomcorn millet (Supplementary Fig. 26). A 10.3-kb deletion in *longmi009317* was responsible for the absence of this gene in several C1 and C3 accessions (Supplementary Fig. 26b). Similarly, a 3.2-kb deletion in *longmi003952* led to the loss of six exons (Supplementary Fig. 26f). The frequency of the truncated gene *longmi003952* was higher in C1 (89.0%), C2 (38.7%) and C3 (44.4%) than in W1 (3.2%) (Supplementary Fig. 26f). However, comparing the phenotypes of the accessions carrying the deletion with those carrying wild-type (WT) alleles showed only slight differences in seed SHT (Supplementary Fig. 26h), which implies that the function of the mutated genes was compensated by their homoeologous counterparts (Supplementary Fig. 26i–k). Furthermore, we detected a PAV that truncated *longmi058828*, the ortholog of *OsCAD2* (ref. ^48^); this mutation was associated with easy SHT (Supplementary Fig. 27a–d). The frequency of the truncated alleles (Ref) in the wild population was greater than in the cultivated populations (Supplementary Fig. 27b). We also identified *longmi012879*, the ortholog of *SSH1*/*OsSNB*^49^, in a selective region (Supplementary Fig. 27e). Its Hap 4 was significantly associated with seed SHT in wild accessions (Supplementary Fig. 27f,g). These observations indicate that multiple genomic variations associated with the non-SHT phenotype may have been selected during broomcorn millet domestication.

To identify further genomic variations controlling SHT in broomcorn millet, we analyzed the GWAS data and identified 58 SNPs from 13 chromosomal locations significantly associated with SHT (Supplementary Fig. 28a and Supplementary Table 20). We found two genes, *longmi020192*, encoding pectinesterase (PE) and *longmi028230*, encoding the pectinesterase inhibitor (PEI), in the identified selective sweeps (Fig. 5c and Supplementary Fig. 28b,c,f,g). PE is responsible for pectin degradation in the middle lamella, while PEIs can inhibit the de-esterification of pectin methylesterases. Genes encoding PE have been associated with the abscission of oil palm fruits and bean leaves^50,51^, implying similar functions in the abscission zones of broomcorn millet. We identified two nonsynonymous SNPs in the coding regions of *longmi020192* and *longmi028230*. In addition, the Haps carrying these nonsynonymous mutations were highly correlated with seed SHT in wild accessions (Fig. 5d,e and Supplementary Fig. 28d,e,h,i). These findings suggest that *longmi020192* and *longmi028230* have undergone selection for the non-SHT phenotype during broomcorn millet domestication.

#### Inflorescence and seed color

Inflorescence and seed color are traits associated with plant adaptation, stress response and nutrition content^52,53^. Morphological surveys revealed that green inflorescence and dark-colored seed were preferred in cultivated populations of broomcorn millet (Fig. 5b), indicating the selection of these traits. The SNP-GWAS identified 1,211 SNPs on chromosome 4 associated with inflorescence color (IFC) (Fig. 5f). Two SNPs were found in the regulatory regions of *longmi008332*, encoding a glutathione *S*-transferase (Fig. 5f and Supplementary Fig. 29a), associated with anthocyanin accumulation in plants^54^. These two SNPs formed two major Haps, with most Hap 2 accessions having purple inflorescence and most Hap 1 accessions exhibiting green inflorescence (Supplementary Fig. 29b–f). Moreover, the PAV-GWAS identified a 72-bp insertion in *longmi008332* associated with purple inflorescence (Fig. 5g,h and Supplementary Fig. 25b). All accessions without the insertion (308 of 308) had Hap 1, while 76.6% (128 of 167) of the accessions with this insertion exhibited Hap 2 (Supplementary Fig. 29g), suggesting that the 72-bp insertion was the mutation responsible for purple inflorescence in broomcorn millet.

Several loci associated with seed color (SDC) were detected on chromosomes 5, 6, 9, 11 and 14 in the SNP-GWAS (Supplementary Fig. 30a). Among these, 483 associated SNPs were found on chromosome 9, centered around a tryptophan decarboxylase (*TDC*) gene cluster (*longmi004409*, *longmi004412* and *longmi004413*) (Supplementary Fig. 30a,b). *TDC* is a gene associated with serotonin biosynthesis; its upregulation leads to dark brown seeds or leaves^55^. Hap analysis revealed that Hap 3, 4 and 5, associated with dark seed coats, were present primarily in cultivated accessions (Supplementary Fig. 30c–f). Furthermore, an SNP caused a premature stop codon in *longmi057520* and was highly associated with dark seeds (Supplementary Fig. 31a–e). *Longmi057520* is homologous to *GH2*, synthesizing the coniferyl and sinapyl alcohol precursors in rice^56^. The *GH2* mutant seeds were golden yellow, while the WT seeds were light yellow^56^. Therefore, the premature stop codon in *longmi057520* probably led to dark seed in cultivated broomcorn millet. In addition, *longmi057520* was located in a selective sweep (Supplementary Fig. 31f,g). These observations suggest that SDC is a complex trait controlled by multiple genetic factors that were reformed during broomcorn millet domestication, favoring dark SDCs.

#### Panicle architecture

Panicle shape is a crucial determinant of grain yield and is a focus of crop domestication and improvement^57,58^. In broomcorn millet, wild accessions have open panicles, while cultivated accessions have closed panicles (Fig. 5b,i), leading to high yields. We conducted the SNP-GWAS analysis on eight panicle shape-related traits (Fig. 5b) and identified 55 genes associated with four panicle-related traits on chromosome 14 (Fig. 5j and Supplementary Fig. 32a,b). Among them, *longmi038370* encodes an SBP-domain protein, an ortholog of *LG1* that controls leaf angle, tassel branch number and tassel branch angle in maize^59^, and inflorescence architecture in rice^57,58^. Hap analysis revealed that Hap 1 and 5 were strongly associated with lower inflorescence density (DOI) and larger branches of grain ears and main shafts (Supplementary Fig. 32c–j). Moreover, *longmi038370* was located in a selective sweep (Supplementary Fig. 32k,l), suggesting it was under selection during broomcorn millet domestication. To validate the function of *longmi038370*, we generated three CRISPR–Cas9 mutants of *Seita.3G022100.1* (*SiLG1*), its orthologous gene in foxtail millet (Supplementary Fig. 33a). The *Silg1-1* mutant showed loss of ligule (Fig. 5k,l), a smaller angle between the panicle branch and main stem (Fig. 5m,n and Supplementary Fig. 33b) and compact panicles (Fig. 5o). Thus, we concluded that *longmi038370* controls panicle shape in broomcorn millet.

In addition, we identified loci on chromosomes 1 and 2 associated with the main shaft of ear direction (MED) (Fig. 5j), containing a candidate gene, *longmi054645*, encoding a no apical meristem protein (Supplementary Fig. 34a). Hap analysis revealed that Hap 3 was strongly associated with low MED in wild accessions (Supplementary Fig. 34b–e). Another candidate gene, *longmi011403*, encoding a calcium-dependent phosphotriesterase protein, was also identified on chromosome 1 (Supplementary Fig. 34f). Hap 2 of *longmi011403* was exclusively present in wild accessions; it was associated with lower DOI and open panicles (Supplementary Fig. 34g–l), suggesting that it is pleiotropic and controls multiple panicle traits in broomcorn millet.

## Discussion

Broomcorn millet is a promising alternative crop for semiarid regions^6,7,17^. At the start of this study, there were only 14 cultivars in the United States^17^ and 222 cultivars in the National Crop Genebank of China, indicating the urgent need for developing a genomics-assisted breeding system in broomcorn millet. We constructed a graph-based pangenome and conducted a GWAS in the population. These data helped us elucidate the domestication history of broomcorn millet and identify genomic signatures underlying domestication and agronomically important traits in broomcorn millet.

Our study clarifies the domestication history of broomcorn millet and directions for future research to resolve its spread routes, which can reveal the origins of agriculture, languages and human societies across the globe^60^. The genomic analyses, corroborated by archaeological evidence from Northern China (8,700–11,500 BP)^9,61^, suggest that broomcorn millet was domesticated in Northern China. Xinjiang, a major agricultural and cultural hub between East and West Eurasian countries^62^, may have served as an exchange hub for the spread of broomcorn millet. This is supported by evidence of shared variants in ancient DNA from the Xiaohe cemetery (3,400–4,000 BP)^63^ and current European accessions. Further studies using additional samples from Gansu, Xinjiang and Eastern Europe may help clarify the spreading routes and their relevant timings. Moreover, researchers found that foxtail millet was domesticated in Northern China slightly later than broomcorn millet, indicating that it may have spread across Eurasia via different routes or at different periods over thousands of years^8,9,64,65^. A study comparing both millets will help reveal when, where and how these crops have spread and adapted across Eurasia. However, these questions cannot be resolved entirely using archaeological remains because of limitations and challenges in processing these datasets^30,64^.

Our study also sheds light on the effects of polyploidy on the domestication of broomcorn millet. Recent studies revealed mechanisms on polyploid evolution, such as homoeologous exchanges, selection on coexpression networks and enhanced adaptive abilities driven by gene fractionation^66–68^. Our study revealed that TE-derived PAVs contributed to 68.3% of total PAVs in broomcorn millet. Although most PAVs were deleterious, the polyploid genomes probably buffered these variants by compensating their functions with homoeologous genes. This is evidenced by the observation of deletion or pseudogenization of three homologs of known SHT genes in the wild population of broomcorn millet. Typically, no obvious subgenome dominance in gene expression is detected in the tetraploid broomcorn millet^42^. However, subgenome B contains more TEs and has experienced an excess of gene loss^42^. We found that artificial selection favored subgenome A. Researchers argued that, as in hexaploid wheat, broomcorn millet’s subgenome A probably contains more functional genes regulated within subgenome-specific chromatin territories^69^. Thus, the present study’s findings with the earlier reports indicate that artificial selection may have driven biased gene expression in one subgenome over the other, leading to unbalanced gene expression between two subgenomes in specific regulatory modules. However, this bias needs to be explored further.

In conclusion, our study has generated a comprehensive dataset that integrates genomic and phenotypic variations in broomcorn millet. The genomic resources described in this study will serve as a foundation for studying the genetic basis of other agronomically important traits, such as nutrient content, salt and drought tolerance, disease and pest resistance in broomcorn millet, and building a genomics-assisted breeding system in broomcorn millet.

## Methods

### Plant materials, growth conditions and field phenotyping

A total of 516 broomcorn millet accessions were obtained from the National Crop Genebank of China at the Institute of Crop Sciences, Chinese Academy of Agricultural Sciences, Beijing. This diverse collection included 415 landraces, 38 breeding lines and 63 wild accessions, collected from 16 provinces across China and countries such as Afghanistan, Pakistan, Mexico, South Korea, Japan, Russia, France and Belgium, among others. Hence, it covered almost all areas of broomcorn millet cultivation, offering a comprehensive view of the crop’s genetic diversity.

To evaluate the phenotype, we planted these accessions at seven sites across China (Fig. 5a), representing diverse environmental conditions, including regions in Heilongjiang (Qiqihar: 47.05° N, 124.33° E), Inner Mongolia (Chifeng: 42.15° N, 118.52° E; Huhehaote: 40.53° N, 110.40° E), Shanxi (Taiyuan: 37.31° N, 112.29° E; Datong: 39.44° N, 113.30° E), Hebei (Shijiazhuang: 37.27° N, 113.30° E) and Gansu (Baiyin: 36.55° N, 104.17° E). Good-quality and plump seeds of uniform size (80 per accession) were sown in the fields in 2019 and 2020. We evaluated 43 phenotypic traits using a quantitative and descriptive method published for descriptors and data standards^70^, maintaining three individual plants per accession. Seed dimension traits, such as seed width, length and weight, were analyzed using the SC-G software (Hangzhou Wanshen Detection Technology).

### Short-read sequencing, Hi-C sequencing and data processing

Genomic DNA was extracted from the mature leaves of 516 accessions and used to construct 150-bp paired-end sequencing libraries with an insert size of approximately 350 bp, sequenced on an MGISEQ-2000 platform (MGI Tech). Raw reads were filtered with Trimmomatic (v.0.39) to remove low-quality bases and sequencing adapters^71^ and the clean reads were aligned to the Longmi4 reference genome using Burrows–Wheeler Aligner (BWA)-MEM in SpeedSeq (v.0.0.2)^72,73^ with default parameters. Genomic variations, including SNPs and indels, were identified with the Genome Analysis Toolkit UnifiedGenotyper (v.3.8)^74^ and filtered using the following parameters: QD < 2.0; MQ < 40.0; FS > 60.0; AF < 0.05; HaplotypeScore > 13.0; MappingQualityRankSum < −12.5; ReadPosRankSum < −8.0; QUAL < 30.0||DP < 6||DP > 5,000||HRun > 5; MQ0 > = 4 && ((MQ0/(1.0 × DP)) > 0.1) for SNPs and QD < 2.0; ReadPosRankSum < −20.0; FS > 200.0; MQ0 > = 4 && ((MQ0/(1.0 × DP)) > 0.1); QUAL < 30.0||DP < 6||DP > 5,000||HRun > 5 for indels. Finally, clustered SNPs were filtered using the following settings: --clusterSize 3 --clusterWindowSize 10.

The Hi-C libraries were constructed from the seedlings of BC170 and BC418. The seedlings were cut and cross-linked with 2% formaldehyde via vacuum infiltration; glycine was added to the mixture to stop the cross-linking step. Nuclei were purified, digested with 100 units of DpnII and end-labeled via biotinylation with biotin-14-dATP. Ligated DNA was sheared into 300–600-bp fragments, which were end-repaired, A-tailed and purified. Hi-C libraries were quantified and sequenced on a DNBSEQ-T7 platform (MGI Tech). High-quality Hi-C reads were then mapped to the genome with the BWA using the CPU version of Juicer (v.1.6)^75^. After removing multi-mapped and duplicated reads, a Hi-C contact map was generated with Juicer and visualized using the Juicebox Assembly Tools (v.1.11.08)^76^. The Hi-C interaction map was used to evaluate the quality of genome assembly and SVs identified in BC170 and BC418.

### Phylogeny and population structure

To determine the phylogenetic relationships among the 516 broomcorn millet accessions, we first obtained 12,816 4DTv sites from the annotated SNP VCF file using ANNOVAR (v.2020-06-08)^77^ and then processed them using the script calc_4dTv_in_eff_vcf.py. We then used these 4DTv sites to build a maximum likelihood phylogenetic tree in IQ-TREE (v.2.1.4-beta)^78^ using the GTR + R10 model. We also conducted PCA with the 4DTv sites on the 516 broomcorn millet accessions using PLINK (v.1.90b6.18)^79^. We calculated the LD between two SNPs using PopLDDecay (v.3.41)^80^ with the following parameters: MaxDist = 500, minor allele frequency (MAF) = 0.01 and Het = 0.8.

Population structure analysis was analyzed using ADMIXTURE (v.1.3.0)^24^ with the number of clusters (K) ranging from 2 to 15 based on 57,930 pruned SNPs obtained using PLINK with the parameters --indep-pairwise 50 5 0.2. Then, discriminant analysis of principal components (DAPC)^27^ was conducted using adegenet (v.2.1.8)^81^ to determine the optimal K in the broomcorn millet population. In the find.clusters() function, we used 300 PCs, which accounted for approximately 90% of the total genetic variability, to identify the cluster number. The Bayesian information criterion curve indicated that 4–9 clusters were reasonable to summarize the data. We also used fastSTRUCTURE (v.1.0)^26^ and STRUCTURE (v.2.3.4)^25^ to determine the optimal number of clusters. The marginal likelihood of fastSTRUCTURE showed a similar curve with the ADMIXTURE and DAPC analyses, while STRUCTURE identified K = 2 and K = 4 as the optimal number of clusters. We then compared the clusters identified with ADMIXTURE (W1, C1, C2 and C3) with those identified with DAPC, fastSTRUCTURE and STRUCTURE. The results showed that the four clusters identified with ADMIXTURE, DAPC, fastSTRUCTURE and STRUCTURE were consistent, except for a few individuals in the C1 population that were clustered with C3 in the DAPC clusters. Based on these observations, we divided the population into four clusters (W1, C1, C2 and C3) to summarize the population structure of broomcorn millet.

### Identification of selective sweeps

The selective sweeps under artificial selection during domestication and improvement were detected by combining the XP-CLR (v.1.0)^41^, *π*_wild_/*π*_cultivar_ and the *F*_*ST*_. The XP-CLR analysis was run with the window size, window step and maximum SNPs set to 20 kb, 2 kb, and 300, respectively. The top 5% of the scores was used as a threshold for significance and smoothed using 100-kb windows with 10-kb step sizes for each chromosome. Meanwhile, the *π* and *F*_*ST*_ values were calculated using VCFtools (v.0.1.13)^82^ with a 20-kb sliding window and a 2-kb step. We then identified the overlaps among the selective sweeps detected by the XP-CLR, *π*_wild_/*π*_cultivar_ and *F*_*ST*_ using BEDTools (v.2.29.1)^83^.

### Long-read sequencing, assembly and quality assessment

To create the pangenome of broomcorn millet, 32 representative accessions were selected for de novo assembly based on phylogenetic relationship and geographical distribution. Genomic DNA was extracted from the seedlings of these accessions and used to construct PacBio HiFi SMRTbell libraries using the SMRTbell Express Template Prep Kit 2.0 (Pacific Biosciences). The libraries were sequenced on a PacBio Sequel II platform using the circular consensus sequencing mode available through the SMRT Link to generate HiFi long reads. Raw contigs were generated from HiFi long reads using Hifiasm (v.0.14.2-r315)^31^ with the following parameters: -l2 -u. Then, to create chromosome-level assemblies, the contigs from each accession were aligned against the Longmi4 genome, and anchored and oriented according to the alignments into the chromosomes using RagTag (v.2.0.1) (https://github.com/malonge/RagTag).

Furthermore, to evaluate assembly quality, we conducted several analyses. First, we assessed the gene completeness with BUSCO (v.4.0.6)^32^ using the embryophyta_odb10 database and repeat completeness based on the LAI^33^ using LTR_retriever (v.2.9.0)^84^. Then, we measured *k*-mer completeness, base pair quality value and false duplication using Merqury (v.1.3)^85^. We also identified and evaluated large SVs between the assembly and the Longmi4 genome. HiFi long reads were mapped to the breakpoints of these SVs using Minimap2 (v.2.24-r1122)^86^ and manually inspected in the Integrative Genomics Viewer (v.2.9.2)^87^. The assemblies were evaluated based on the HiFi read alignments of 32 accessions at 304 loci (3–17 kb) with genomic differences from Longmi4. Finally, the BC170 and BC418 Hi-C reads were aligned to the corresponding genomes to manually inspect for large SVs of BC170 and BC418, using Juicebox (v.1.11.08)^76^. We evaluated the assemblies using Hi-C chromatin maps of these two accessions (BC170 and BC418) at 22 loci (197–6,114 kb) with genomic differences from Longmi4.

### Gene and transposable element annotation

The protein-coding genes in each genome were annotated using the MAKER2 pipeline (v.2.31.11)^34^, which uses ab initio prediction, transcriptome evidence and homologous protein evidence. Specifically, AUGUSTUS (v.3.4.0)^88^ and SNAP (v.2006-07-28)^89^ were used for ab initio gene prediction based on a generalized Hidden Markov Model using a high-confidence gene set from full-length transcriptome data (BioProject ID: PRJNA872304). The transcriptome evidence included RNA sequencing (RNA-seq) datasets from the leaf tissues of each accession as well as inflorescence, leaf blade, leaf sheath, root, mature seed, seedling, shoot and stem of the Pm_0390 cultivar (BioProject ID: PRJNA431485). The raw reads were processed with Trimmomatic (v.0.39)^71^ to remove adapters and low-quality reads and mapped to the corresponding genome using HISAT2 (v.2.1.0)^90^ with the following parameters: --min-intronlen 20 --max-intronlen 15,000. The full-length transcript sequences of each genome were assembled using StringTie2 (v.2.1.7)^91^ with default parameters. The homologous protein evidence was obtained from *P. miliaceum* (Longmi4), *P. hallii*, foxtail millet, *Sorghum bicolor*, *Brachypodium distachyon* and *Arabidopsis thaliana*, and the UniProt proteins of Embryophyta. Protein-coding genes were functionally annotated using InterProScan (v.5.52-86.0)^92^. Finally, repetitive sequences in each genome were identified and classified using RepeatModeler (v.2.0.1)^93^ and annotated with RepeatMasker (v.4.0.9) (http://www.repeatmasker.org) with the following parameters: -e rmblast -div 40 -norna.

### Gene-based pangenome analyses

A gene-based pangenome of 32 broomcorn millet accessions was constructed according to the gene family clustering strategy. First, protein sequences with 100% similarity in each genome were removed using Cd-hit (v.4.8.1)^94^ with the following parameters: -c 1 -aS 1. Then, nonredundant protein sequences were clustered into gene families using OrthoFinder (v.2.5.4)^95^. The resulting gene families were classified into core, softcore, dispensable private genes based on their presence in each of the 32 genomes. Gene families in all 32 genomes were defined as core genes, those in 30–31 as softcore genes, those in 2–29 as dispensable genes and those in only one genome as private genes. The ratio of nonsynonymous to synonymous substitution (K_a_/K_s_) for each gene of the pangenome was calculated with the KaKs_Calculator (v.2.0)^96^ using foxtail millet *Setaria italica* as an outgroup based on multiple sequence alignments performed with ParaAT (v2.0)^97^. The *π* for each gene of the pangenome was calculated using in-house Perl scripts based on multiple sequence alignments performed with MAFFT (v.7.475)^98^. The following formula was used to calculate *π*: *π* = D/L/(N × (N−1)/2), where D represents the number of differential sites, L represents the length of the conserved alignment and N represents the number of sequences.

### SV identification and quality assessment

We used a reference-based alignment method called PoPASSYSV (https://github.com/yiliao1022/PoPASSYSV) to perform SV calling on 32 genome assemblies. We aligned each query genome against the Longmi4 genome reciprocally using Minimap2 (v.2.17)^86^. We then used the CHAIN/NET/NETSYNTENY tools (https://github.com/ucscGenomeBrowser/kent) to filter out nonorthologous and nonsyntenic alignments, which are not represented in a single coverage for either the reference or the query. The resulting netsyntenic format files obtained from each pairwise comparison were used to call the five subtypes of SVs, including insertion, deletion, inversion, tandem duplication and complex types, using the PairwiseCalling.pl function within the PoPASSYSV toolkit. To filter out any false positives in SV identification, we excluded deletions and insertions that overlapped with the sequencing gaps or centromere repeats, and inversions that overlapped with the gaps within the 10-bp range. Finally, the SVs identified from 32 assemblies were merged to create the consensus SVs for the broomcorn millet population. All deletions and inversions with an overlapping ratio greater than 90% were merged, while insertions with a distance of less than 10 bp and an identity greater than 80% were merged. The merged deletions and insertions (PAVs) were then genotyped across the 516 accessions based on short-read data using Paragraph (v.2.3)^99^ and the vg toolkit (v.1.43.0)^39^.

SV quality was assessed using two approaches based on HiFi read alignment and Hi-C chromatin maps in BC170 and BC418 (Supplementary Tables 12 and 13). To evaluate the PAVs missing in the primary assemblies, alternate contigs from 32 accessions, with sizes ranging from 13 Mb to 1.3 Gb, were used for PAV calling (Supplementary Table 3). A total of 21,256 nonredundant PAVs were identified from these alternate contigs, including 11,047 deletions and 10,209 insertions; 4,911 PAVs (23.1%) were absent in the primary assembly dataset. The accuracy of these PAVs was further evaluated by manually inspecting the PacBio long reads mapped at the breakpoints. The analysis revealed a lower accuracy rate of 51.7% (31 of 60), which is lower compared to those in the primary assemblies (83.3%) (Supplementary Table 12). Therefore, despite providing 9.7% (4,911 of 50,515) more total PAVs, these alternate PAVs were not included in the final analysis because of the false positive SV calls.

### Pangenome graph construction and PAV genotyping

We constructed a graph-based pangenome using 50,515 PAVs and Longmi4 with the vg toolkit and genotyped the PAVs across 516 accessions using short reads. The short reads were first mapped to the pangenome graph using the giraffe function; then, the read support was computed applying the pack function. PAVs were genotyped across each accession using the call function. The precision, recall and F1 score of the PAVs were computed as 0.64, 0.66 and 0.65, respectively. After removing the PAVs containing 90% repeat sequences, the precision, recall and F1 score of all PAVs were computed as 0.69, 0.71 and 0.70, respectively. The genotyping rate of the PAVs was 79.9% (ranging from 69.8% to 85.0%), and the average depth of short reads for the 516 accessions was around 17× (ranging from 9.9× to 28.7×). As 90% of 516 accessions with read coverage ranging from 12.6× to 22.1×, their genotyping rate ranged from 78.6% to 83.2%.

### RNA-seq and differential gene expression analysis

Leaf and root tissues were collected from eight wild and 24 cultivated accessions, maintaining three biological independent experiments per accession. Total mRNA was extracted from these samples using TRIzol reagent (Thermo Fisher Scientific); RNA-seq libraries were prepared for paired-end sequencing on an MGISEQ-2000 platform. Raw reads were filtered using Trimmomatic (v.0.39)^71^ and clean reads were mapped to the Longmi4 genome using subjunc (v.2.0.1)^100^. Read counts and FPKM values were calculated using featureCounts (v.2.0.1)^101^ and the differential expression of the genes between wild and domesticated accessions was analyzed using DESeq2 (v.1.32.0)^102^. Genes with an adjusted *P* ≤ 0.05 and absolute(fold change) ≥ 1.5 were defined as the population’s differentially expressed genes (pop-DEGs). Gene Ontology (GO) and Pfam annotation of pop-DEGs were performed with InterProScan (v.5.52-86.0)^92^, while Kyoto Encyclopedia of Genes and Genomes (KEGG) annotation^103^ was carried out with BlastKOALA (v.2.2)^104^ and KofamKOALA^105^. Finally, the significantly enriched GO terms and KEGG categories were identified using a hypergeometric enrichment test in the R package clusterProfiler (v.4.2.2)^106^, with a *P* ≤ 0.05 as the threshold for significance.

### SNP-based and PAV-based GWAS

We conducted an SNP-GWAS analysis on 43 phenotypes by using 1,890,542 SNPs with an MAF ≥ 0.05 and a missing rate ≤ 0.1. The missing SNP data were imputed with Beagle (v.4.1)^107^. Then, EMMAX (v.beta-07Mar2010)^108^ was used for the association analysis incorporating a Balding–Nichols kinship matrix. The uniform threshold was set at 0.05/*n* (*n* represents the total number of SNPs) for the SNP-GWAS and the significance threshold was approximately *P* = 10^−8^. In addition, we conducted a PAV-GWAS analysis using 19,492 PAVs with an MAF ≥ 0.05 and a missing rate ≤ 0.5. The PAV-GWAS threshold was set at 0.05/*n* (*n* represents the total number of PAVs); the significance threshold was approximately *P* = 10^−6^. The associations were considered to be reliable only if they occurred at the same location for at least 2 years or in multiple locations.

Finally, we searched for significantly associated SNPs within 200 kb upstream and downstream regions to detect the potential regions of interest in the GWAS analysis. If significant SNPs were detected, we extended the search to the next 200-kb interval until no more significant SNPs were found. The boundaries of the candidate regions were defined based on the last significantly associated SNP in that region. To further identify the potential candidate genes within the candidate regions, we analyzed the Haps of the protein-coding genes using CandiHap (v.1.0.1) (https://github.com/guokai8/CandiHap). The genes with Haps significantly correlated with the phenotypes were considered as potential candidate genes.

### Functional verification of *longmi038370*

To validate the function of *longmi038370*, we knocked out its orthologous gene *SiLG1* (*Seita.3G022100*) in foxtail millet using CRISPR–Cas9 genome editing. Single-guide RNAs (sgRNAs) were designed according to the sequence of foxtail millet *SiLG1*, using targetDesign (http://skl.scau.edu.cn/targetdesign/). The sgRNA target to the third exon of *SiLG1* was selected (Supplementary Fig. 33a) and the pYLCRISPRCas9-MH vector was used for genome-editing^109^. The primers used for vector construction were SiLG1-gR:AAGAAGCTGTGGATCCCAAGgttttagagctagaaat and SiLG1-OsU6a:CTTGGGATCCACAGCTTCTTcggcagccaagccagca. The CRISPR *Silg1* mutants were generated by editing *SiLG1* in foxtail millet (Ci846) through *Agrobacterium tumefaciens*-mediated transformation. Three independent CRISPR mutants were obtained and verified using Sanger sequencing. Ligule and panicle traits, such as BRM were measured in three mutations using five plants.

### Geographical map generation

Information about the geographical location of the world sampled accessions in this study was generated using the ggmap package in R (v.4.1.0) and ArcGIS (v.10.2) (https://www.arcgis.com/). Monthly climate data for minimum, mean and maximum precipitation were retrieved from WorldClim^110^.

### Statistics

Statistical analyses and plotting were performed in R (v.4.1.0) using built-in functions and third-party R packages including tidyverse (v.1.3.1), ggplot2 (v.3.4.3), ggpubr (v.0.4.0) and agricolae (v.1.3-5). A two-tailed Wilcoxon rank-sum test was used to compare the difference of expression or phenotype between two groups with the R built-in function wilcox.test. A one-way analysis of variance was used to determine differences among groups. Pairwise comparisons were conducted using the least significant difference (LSD) method with Bonferroni correction for multiple comparisons using the function LSD.test in the third-party R package agricolae (v.1.3-5). Pearson correlation coefficients (*R*) and *P* values were calculated with the R function cor.test; fitted curves and 95% confidence intervals for linear regression were also calculated.

### Reporting summary

Further information on research design is available in the Nature Portfolio Reporting Summary linked to this article.

## Online content

Any methods, additional references, Nature Portfolio reporting summaries, source data, extended data, supplementary information, acknowledgements, peer review information; details of author contributions and competing interests; and statements of data and code availability are available at 10.1038/s41588-023-01571-z.

### Supplementary information

Supplementary Information Supplementary Figs. 1–34.

Reporting Summary

Supplementary Tables 1–22

### Source data

Source Data Fig. 1 Statistical source data for Fig. 1.

Source Data Fig. 2 Statistical source data for Fig. 2.

Source Data Fig. 3 Statistical source data for Fig. 3.

Source Data Fig. 4 Statistical source data for Fig. 4.

Source Data Fig. 5 Statistical source data for Fig. 5.

## Data Availability

The raw sequences of 516 accessions (BioProject ID: PRJNA603255), the PacBio HiFi reads and RNA-seq data of 32 accessions (BioProject ID: PRJNA847741) and the Hi-C sequences of BC170 and BC418 (BC170: SRR17710547, SRR17710548, SRR17710549 and SRR17710550; BC418: SRR17710545, SRR17710546, SRR17710553 and SRR17710554) have been deposited with the Sequence Read Archive. The assembled pangenome sequences and gene and transposable element annotations are available at *Zenodo* (10.5281/zenodo.6627574). The assembled pangenome sequences have also been deposited with the NCBI genome database; their accession numbers (JAVRMQ000000000–JAVRNV000000000) are listed in Supplementary Table 3. The phenotype data are available at *Zenodo* (10.5281/zenodo.7749727). All study data are included in the main article and supplementary materials. All broomcorn millet accessions are available at the National Crop Genebank of China. Source data are provided with this paper.

## Code availability

All codes and tools used in this study are described in the Methods. Codes are available at *Zenodo* (10.5281/zenodo.8373683)^111^.
