# Supplementary material for: Pangenome analysis reveals genomic variations associated with domestication traits in broomcorn millet
Source: Nat Genet. 2023 Nov 30;55(12):2243–54. doi: 10.1038/s41588-023-01571-z (PMC10703678; doi:10.1038/s41588-023-01571-z)
Supplement: Supplementary file 1 — Supplementary Figs. 1–34. [file 41588_2023_1571_MOESM1_ESM.pdf]

nature genetics

Article https://doi.org/10.1038/s41588-023-01571-z

Pangenome analysis reveals genomic variations associated with domestication traits in broomcorn millet

In the format provided by the authors and unedited

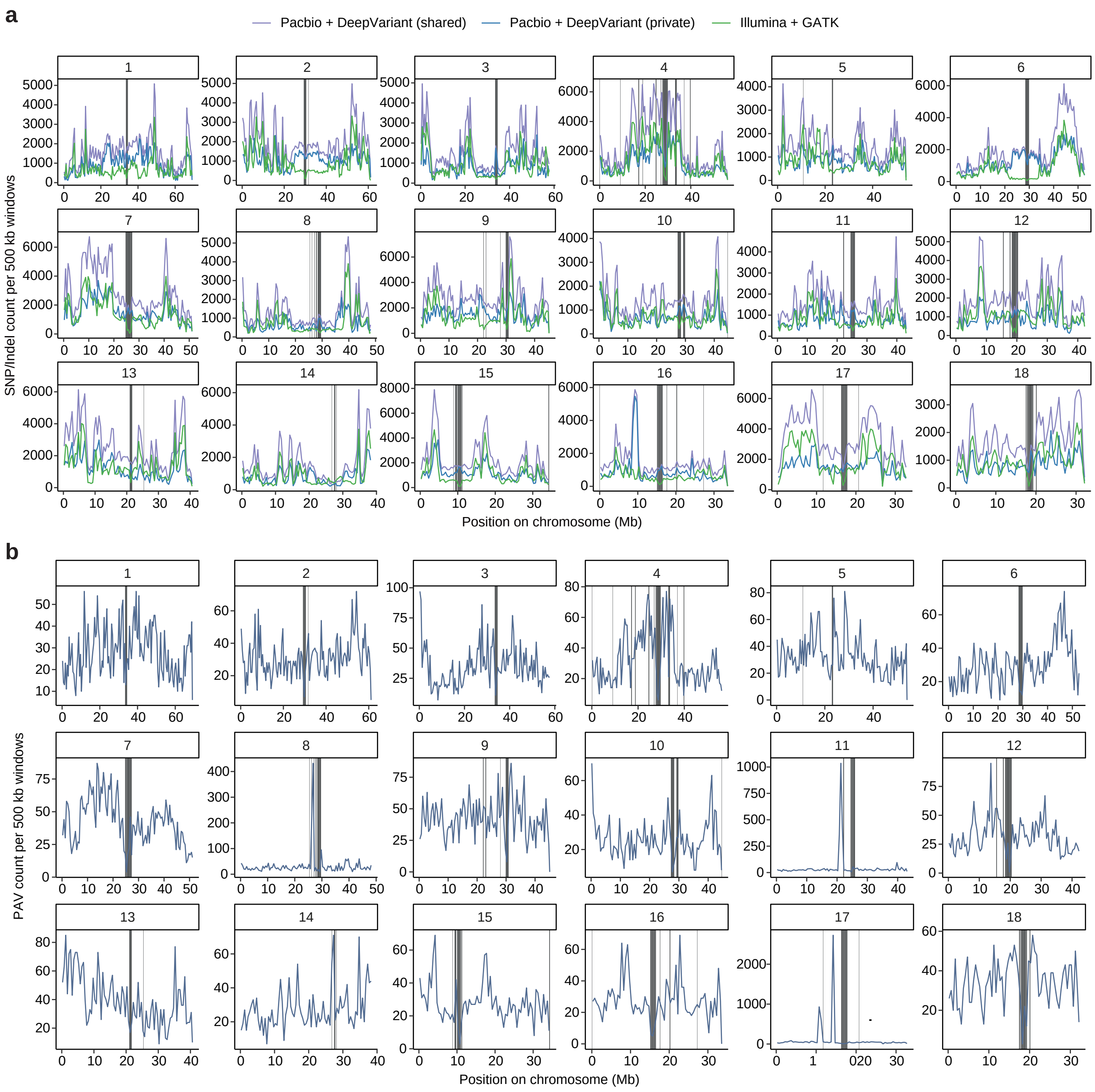

Supplementary Fig. 1. The density of SNPs and PAVs across the chromosomes of broomcorn millet. a, Distribution of SNPs detected by PacBio long-reads, Illumina short-reads, and SNPs specifically detected by PacBio long-reads across the 18 chromosomes of broomcorn millet; **b,** Distribution of PAVs detected by PacBio long-reads across the 18 chromosomes of broomcorn millet. Grey lines represent the centromere repeat regions.

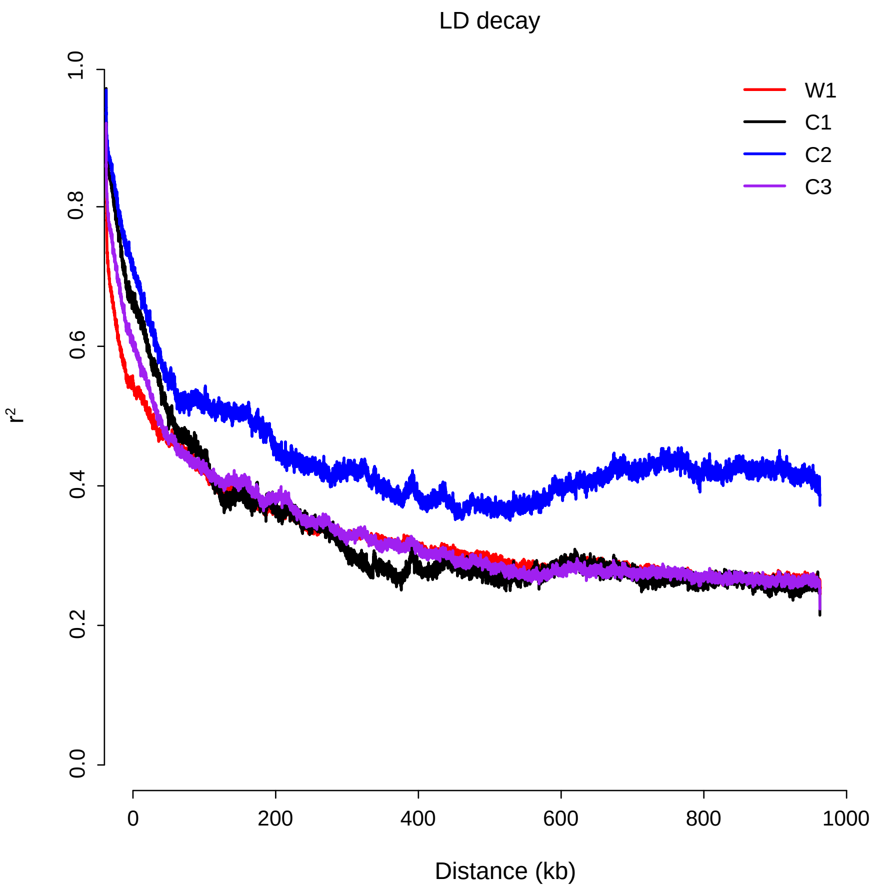

Supplementary Fig. 2. Linkage disequilibrium (LD) decay patterns of the broomcorn millet population. LD levels were estimated with PopLDdecay based all high-quality SNPs (1,890,542). Colors represent populations identified with ADMIXTURE.

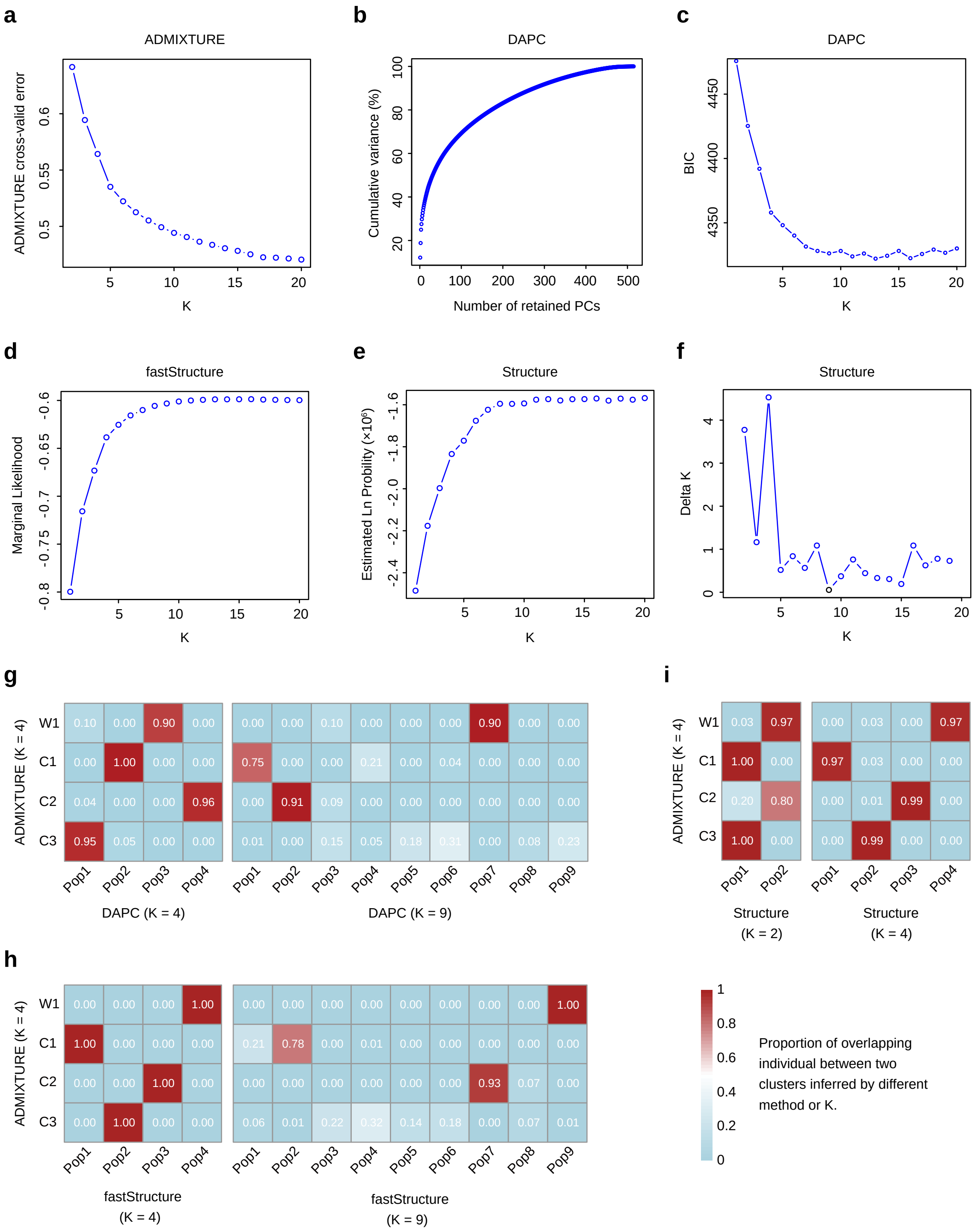

Supplementary Fig. 3. Inference of the number of clusters in the broomcorn millet population. a, Cross-valid error of ADMIXTURE from K = 1 to 20; **b,** Cumulative variance (%) of DAPC from K = 1 to 500; **c,** Bayesian Information Criterion (BIC) of DAPC from K = 1 to 20; **d,** Marginal likelihood of fastStructure from K = 1 to 20; **e,** Estimated log-normal probability of Structure from K = 1 to 20; **f,** Delta K of Structure from K = 1 to 20; **g,** Comparison of clusters identified by ADMIXTURE (K = 4) and DAPC (K = 4, K = 9); **h,** Comparison of clusters identified by ADMIXTURE (K = 4) and fastStructure (K = 4, K = 9); **i,** Comparison of clusters identified by ADMIXTURE (K = 4) and Structure (K = 2, K = 4).

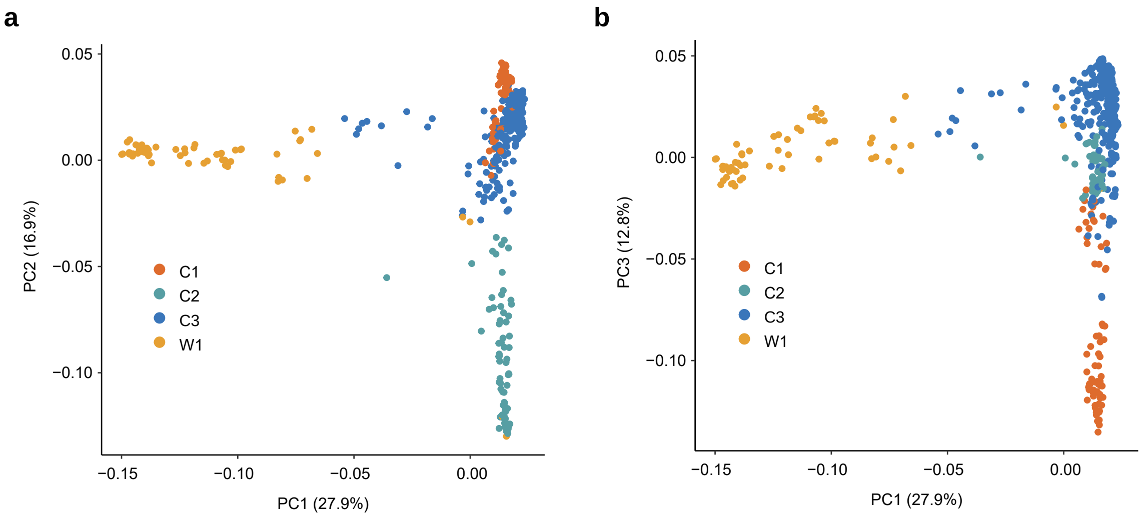

Supplementary Fig. 4. Principal component analysis (PCA) of 516 broomcorn millet accessions. a, PC1 vs. PC2; **b,** PC1 vs. PC3. First three PCs were plotted. Colors represent populations identified with ADMIXTURE.

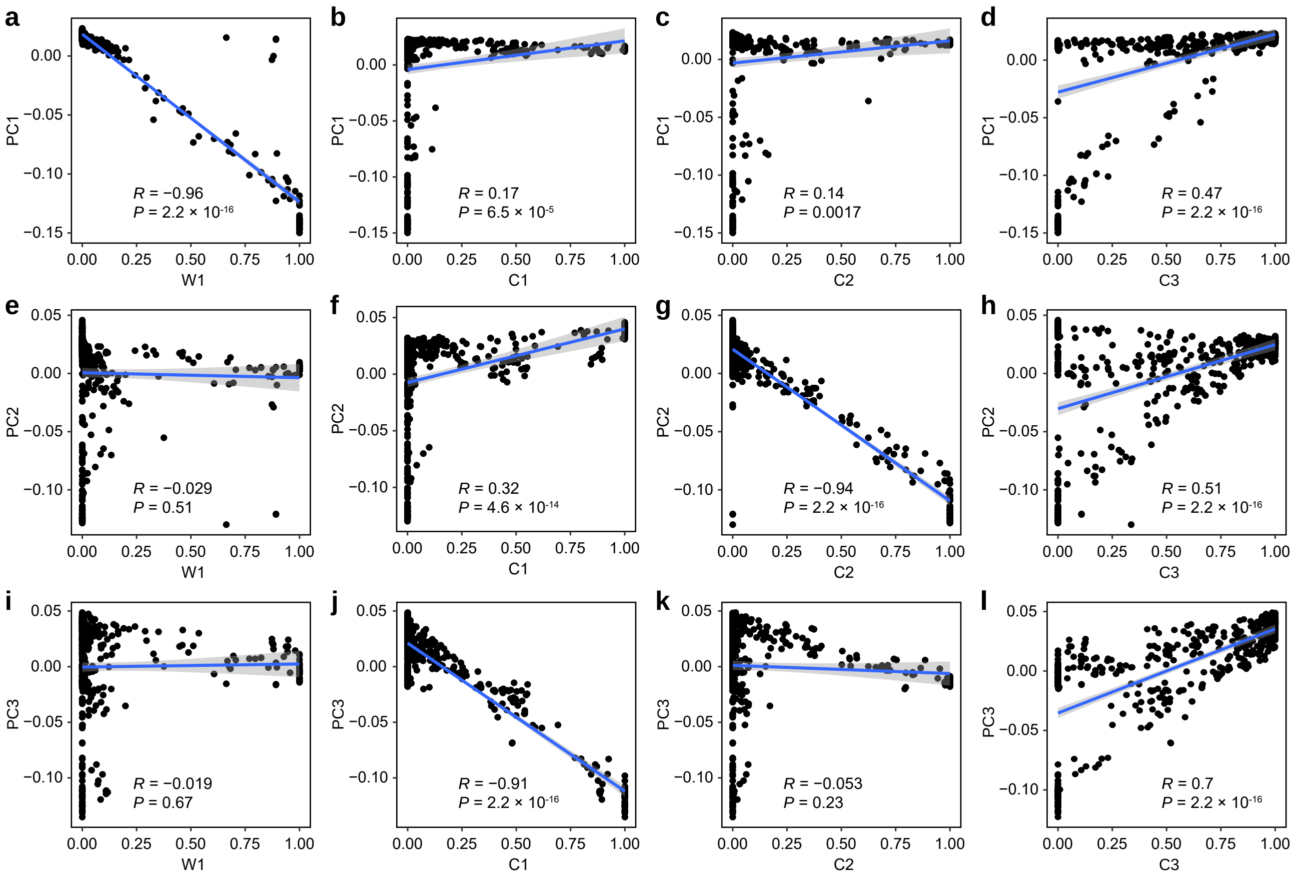

Supplementary Fig. 5. Correlation between the principal components and ADMIXTURE populations of broomcorn millet. a, PC1 vs. W1; **b,** PC1 vs. C1; **c,** PC1 vs. C2; **d,** PC1 vs. C3; **e,** PC2 vs. W1; **f,** PC2 vs. C1; **g,** PC2 vs. C2; **h,** PC2 vs. C3; **i,** PC3 vs. W1; **j,** PC3 vs. C1; **k,** PC3 vs. C2; **l,** PC3 vs. C3. Pearson correlation between each principal component and population is indicated in the figure. Pearson's correlation coefficient (*R*) and *P* value was calculated with R function cor.test. PC1, PC2, and PC3 are negatively correlated with W1, C2, and C1, respectively. Blue lines indicate fitted curves for linear regression. The grey shaded areas represent 95% confidence interval.

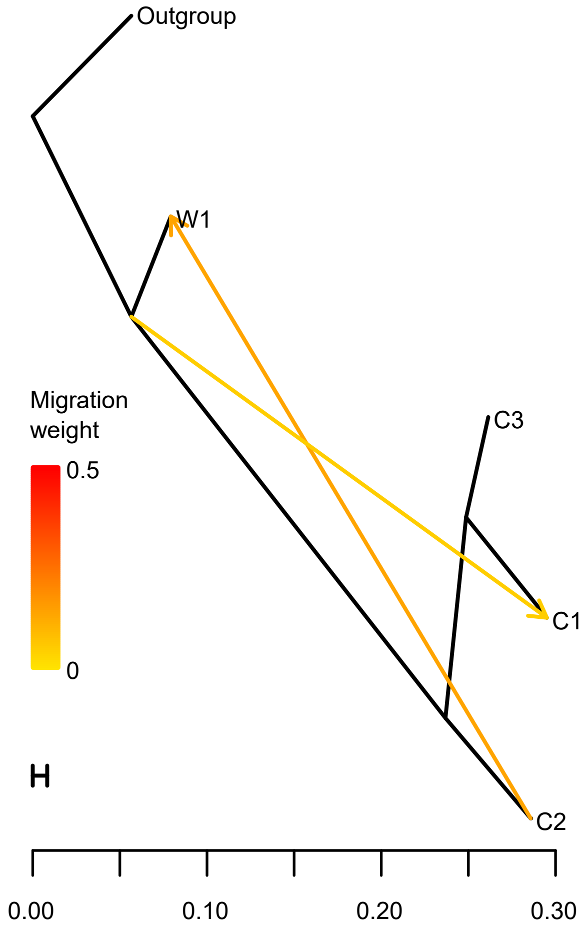

Supplementary Fig. 6. Broomcorn millet population tree with mixture events. Population tree was inferred with TreeMix allowing two migration events. Migrations are indicated by arrows. Colors represent migration weight.

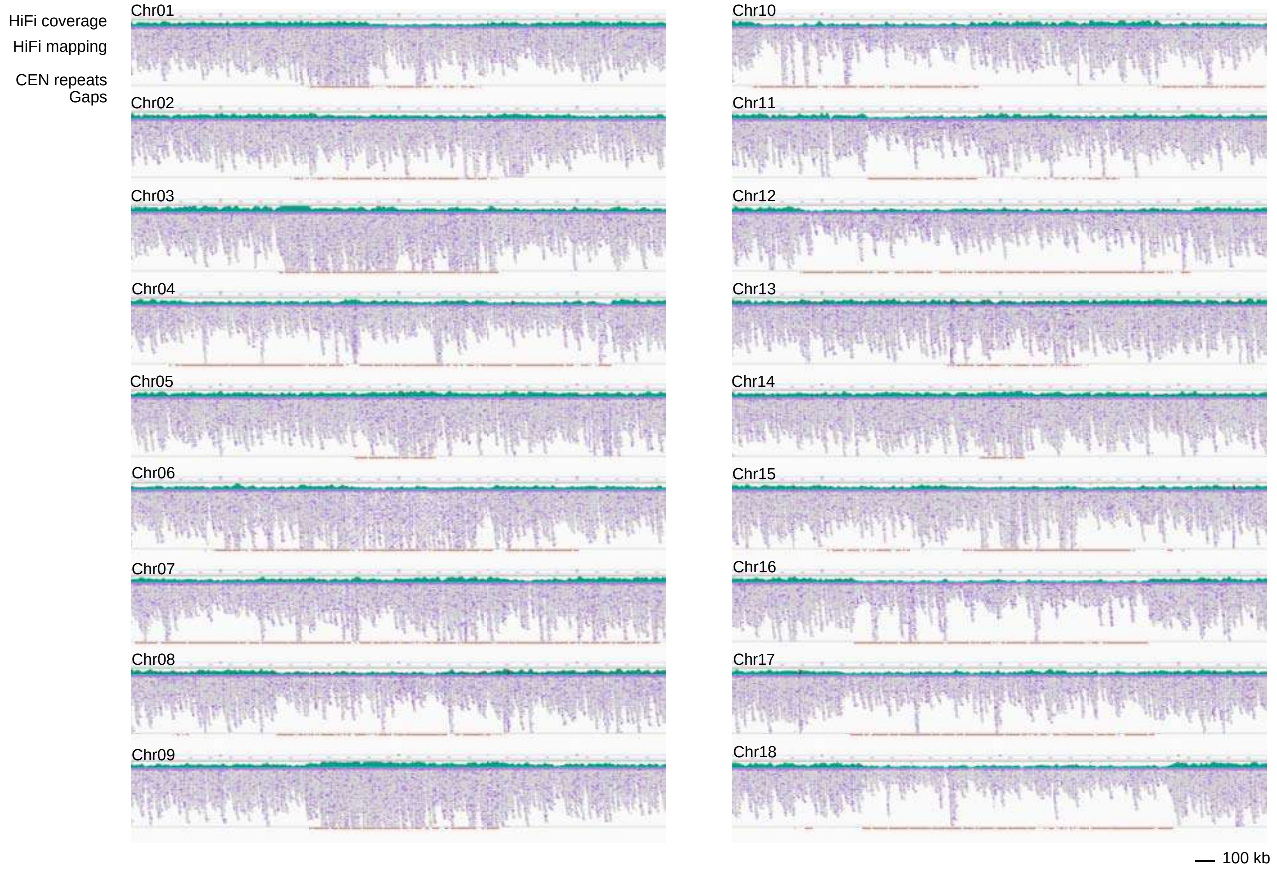

Supplementary Fig. 7. PacBio HiFi read alignment to the centromeric regions in BC475. Centromeric region in all 18 chromosomes of BC475 are shown. Tracks from top to bottom are HiFi read coverage, HiFi reads mapping, centromeric repeats, and gaps, respectively.

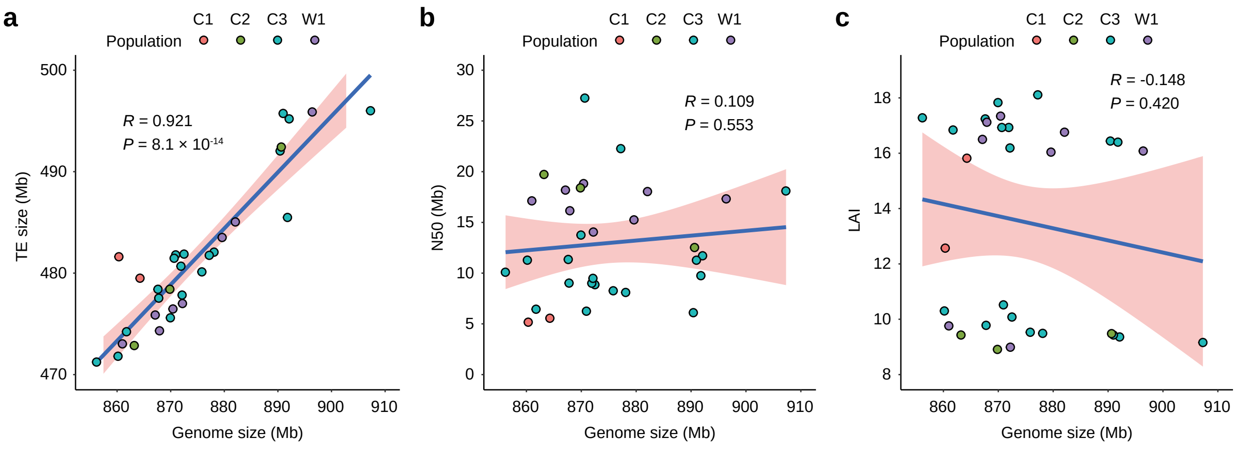

Supplementary Fig. 8. Correlation analyses of genome size with transposable element (TE) content, contig N50, and LTR Assembly Index (LAI). a, Genome size and TE size; **b,** Genome size and contig N50; **c,** Genome size and LAI. Pearson's correlation coefficient (*R*) and *P* value was calculated with R function cor.test. Thirty-two points in each subgraph represent 32 genomes. Blue lines indicate fitted curves for linear regression. The pink shaded areas represent 95% confidence interval.

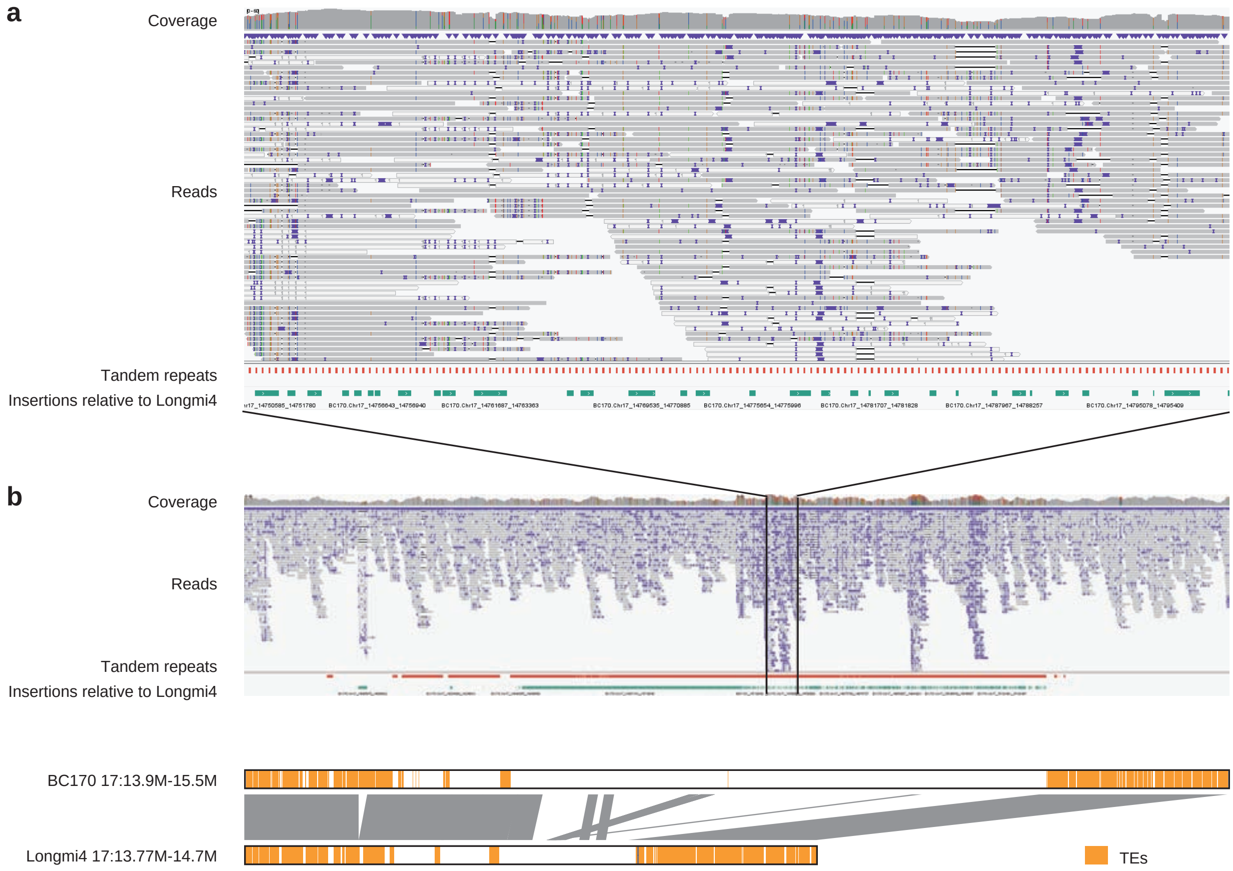

Supplementary Fig. 9. A region with tandem repeat cluster in BC170. a, A zoom-in of a tandem repeat region. IGV tracks from top to bottom are HiFi read coverage, HiFi reads mapping, tandem repeats, and insertions identified in BC170 relative to Longmi4, respectively; **b,** Sequence comparison between BC170 and Longmi4 showing incomplete assembly of Longmi4 in this region and a large tandem repeat cluster in BC170.

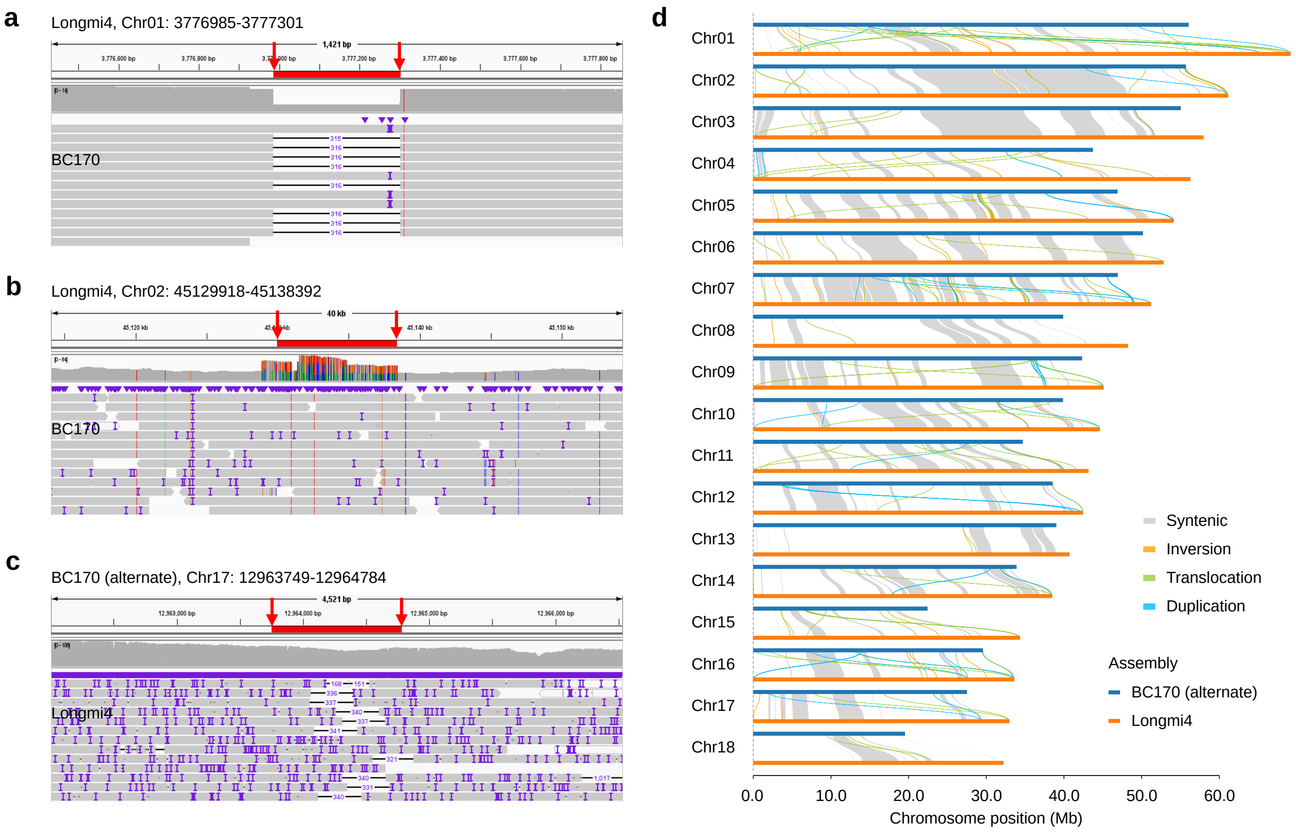

Supplementary Fig. 10. Manual inspection of PAVs in the alternate assembly using PacBio HiFi read alignments. **a,** Example of a confirmed deletion between Longmi4 and BC170 alternate assembly; **b,** Examples of an ambiguous call of deletion between Longmi4 and BC170 alternate assembly; **c,** Examples of an ambiguous call of insertion between Longmi4 and BC170 alternate assembly; In **a**-**c**, the red block represents identified PAVs; **d,** Synteny between Longmi4 and BC170 alternate assembly.

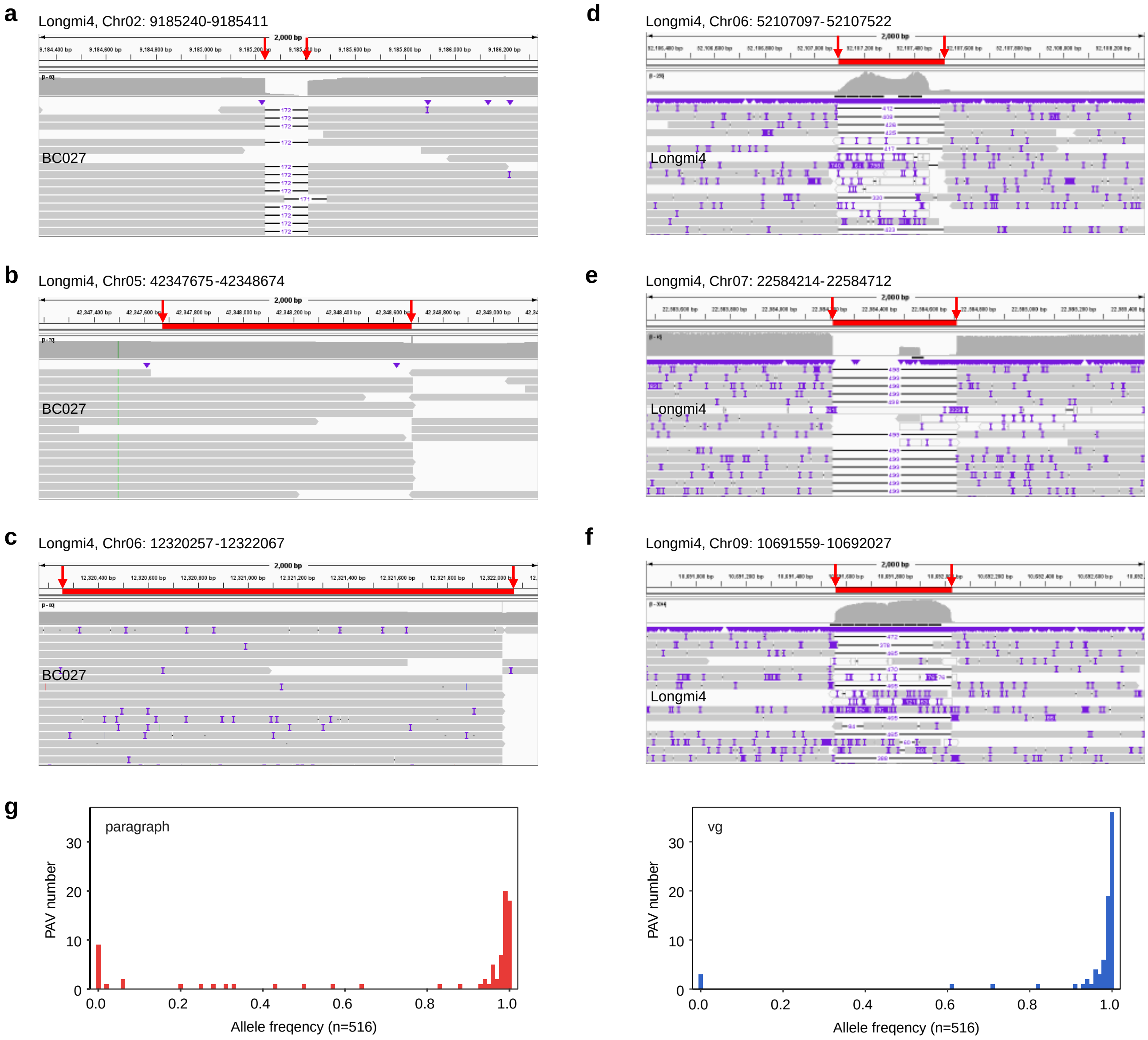

Supplementary Fig. 11. Analysis of the 79 shared PAVs in the broomcorn millet population. a, Read mapping at the junctions of a true PAV using PacBio HiFi reads of BC027; **b-c,** Read mapping at the junctions of false positive PAVs due to unknown causes using PacBio HiFi reads of BC027; **d-f,** Read mapping at the junctions of false positive PAVs due to assembly errors in Longmi4 using PacBio HiFi reads of BC027. We assigned these three PAVs as false positives because the sequence of Longmi4 was not well supported by Longmi4 long reads. It is also possible that these PAVs are real because they are heterozygous in Longmi4. Red bars indicate identified PAV regions. Red arrows indicate PAV junctions; **g,** The frequency of the 79 shared PAVs in 516 broomcorn millet accessions. Allele frequency of PAVs were genotyped by paragraph and the vg toolkit, respectively.

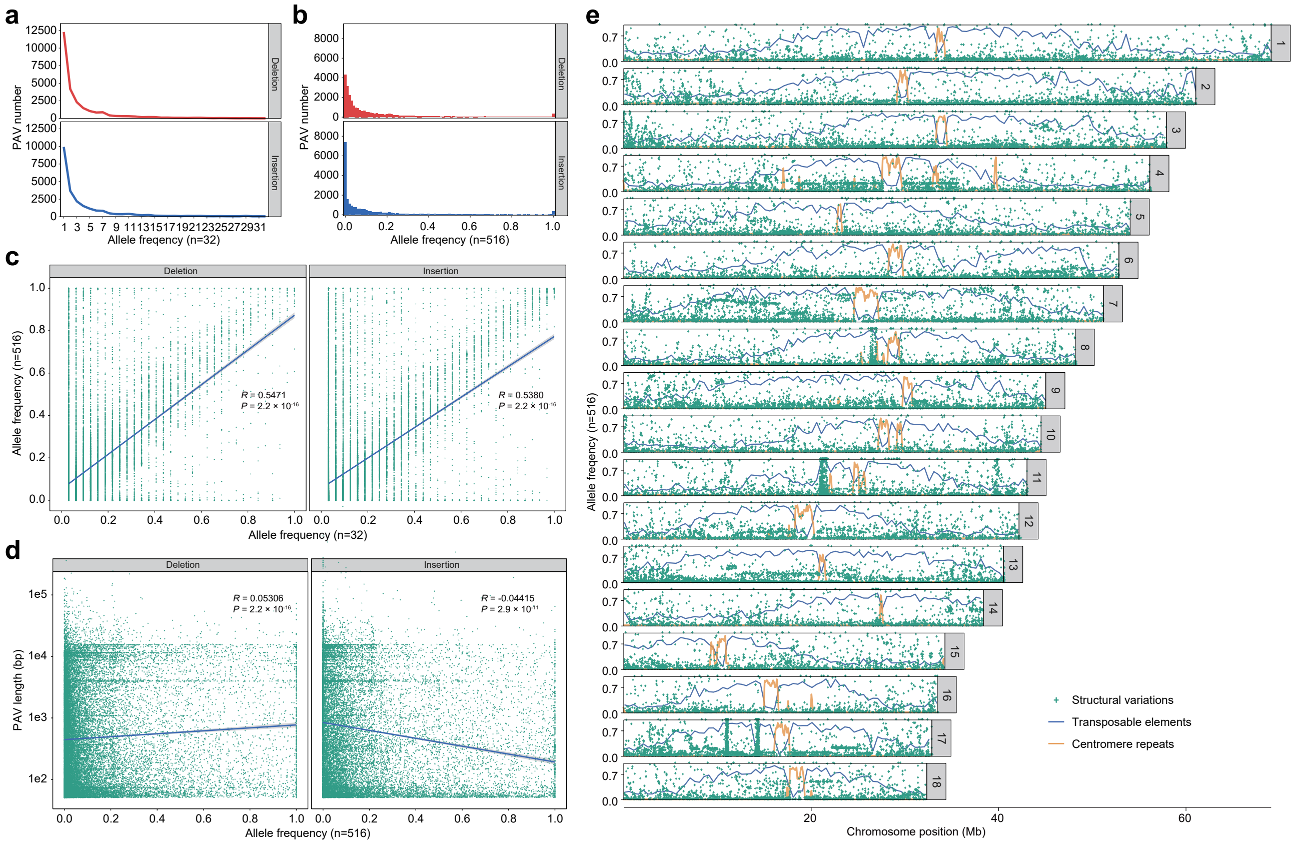

Supplementary Fig. 12. PAV frequency in the broomcorn millet population. a, The frequency of PAVs in 32 broomcorn millet accessions; **b,** The frequency of PAVs in 516 broomcorn millet accessions. PAVs were genotyped with the vg toolkit in 516 accessions; **c,** The correlation of allele frequency between PAVs in 32 (*x*-axis) and 516 accessions (*y*-axis); **d,** The correlation between PAV frequency and PAV length; **e,** Distribution of PAVs along chromosomes of Longmi4. In **c** and **d**, Pearson's correlation coefficient (*R*) and *P* value was calculated with R function cor.test based on 50,515 PAVs. Blue lines indicate fitted curves for linear regression.

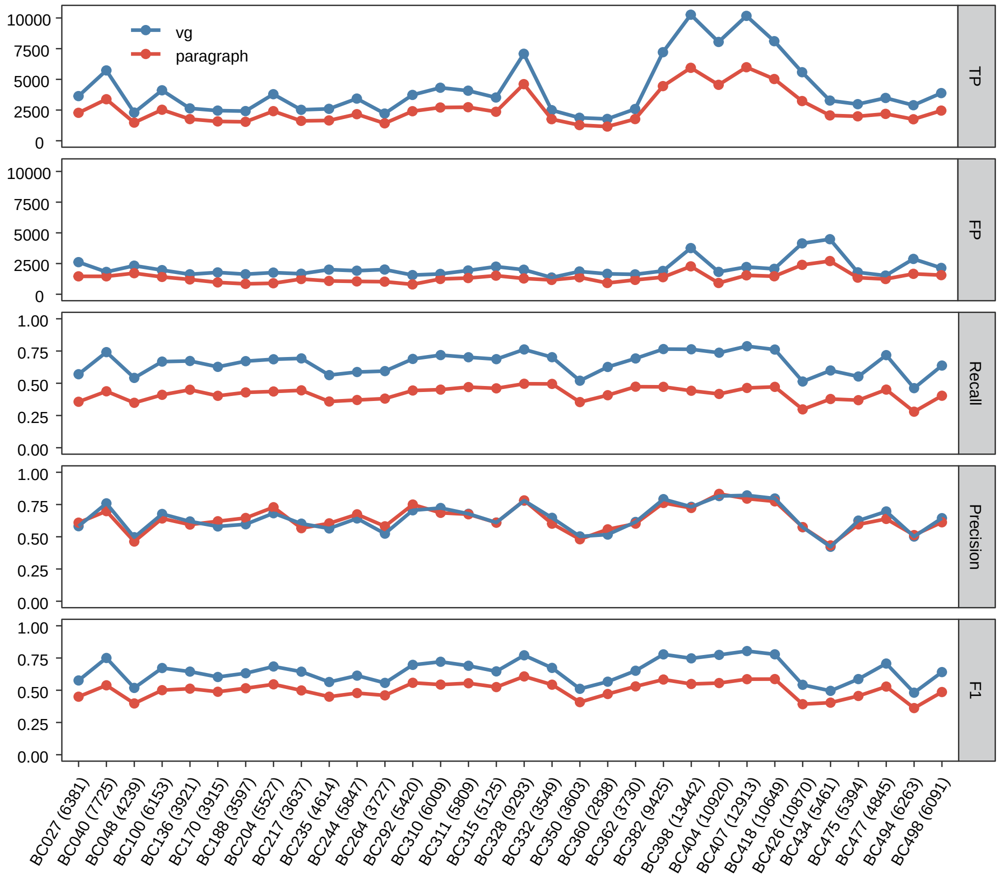

Supplementary Fig. 13. Performance comparison between the vg toolkit and paragraph in 32 broomcorn millet accessions. True positive (TP) was defined as number of PAVs identified using 32 assemblies (the number was in the parentheses, or REAL) and also identified with the vg toolkit using short reads in the same accession. False positive (FP) was number of PAVs not identified using 32 assemblies but were identified with the vg toolkit using short reads in the same accession. Recall was defined as the count of TPs divided by the count of REAL. Precision was defined as the count of TPs divided by the count of TPs plus FPs.

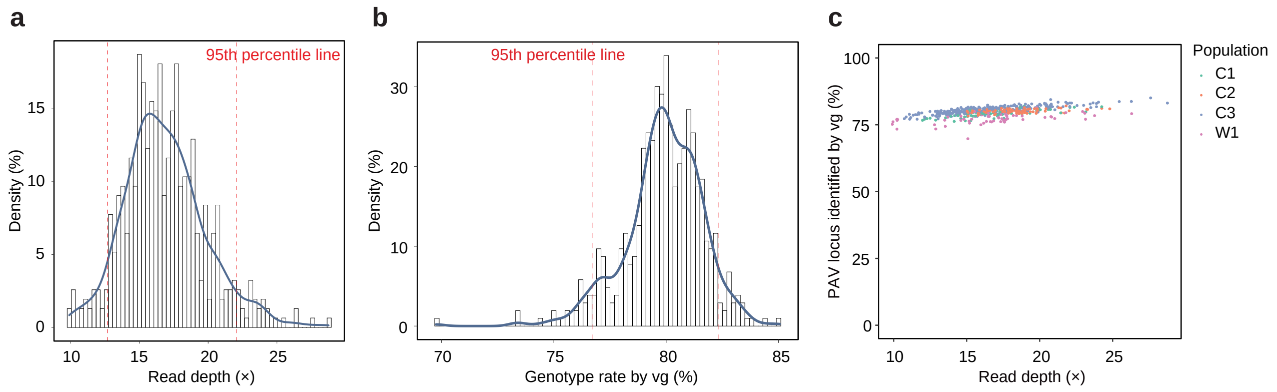

Supplementary Fig. 14. Distribution of read depth and PAV genotyping rate in 516 broomcorn millet accessions. a, Read depth of short reads in 516 broomcorn millet accessions; **b,** PAV genotyping rate by the vg toolkit in 516 broomcorn millet accessions; **c,** Scatter plot of PAV genotyping rate against read depth in 516 broomcorn millet accessions.

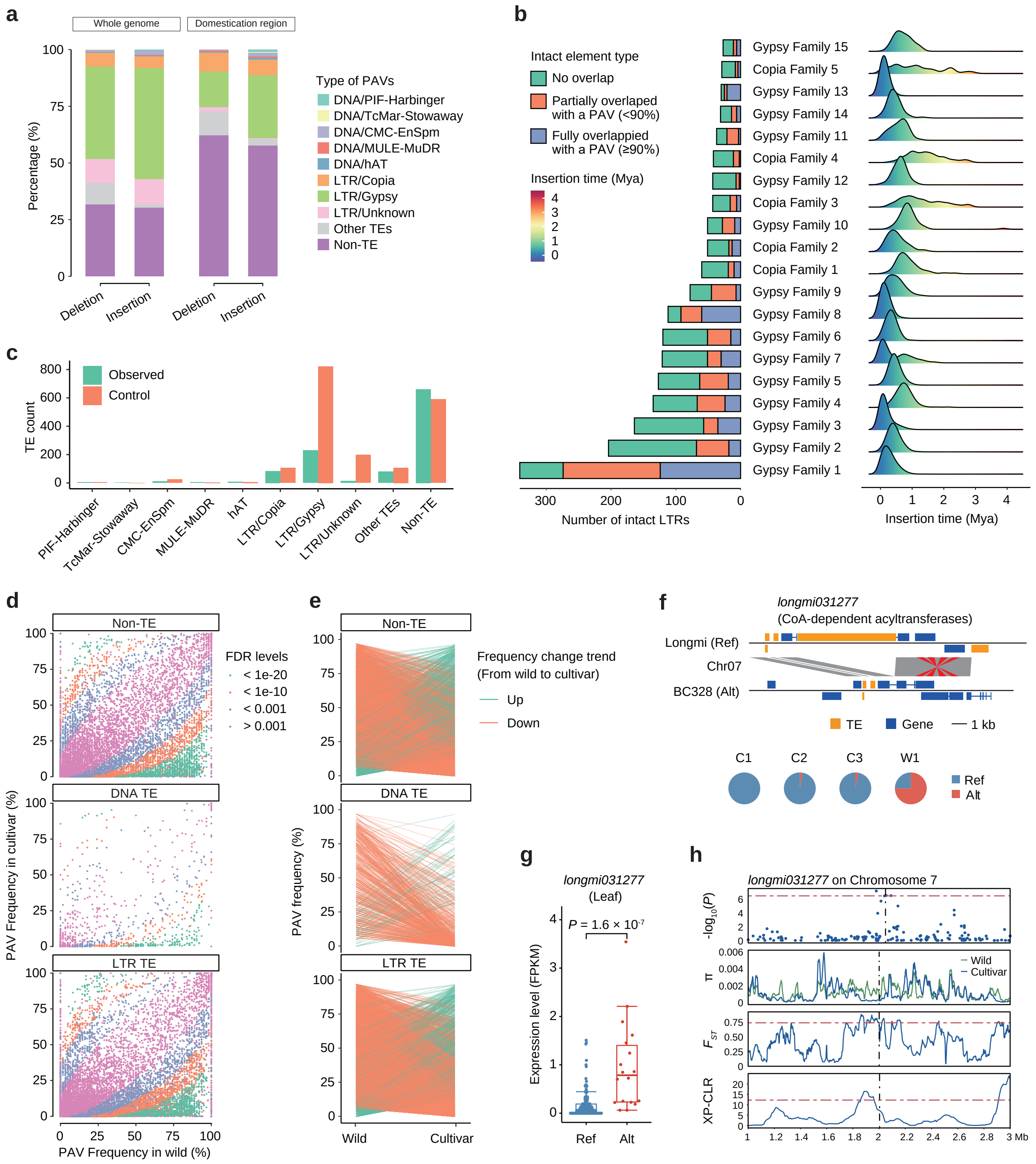

Supplementary Fig. 15. Landscape of TE-derived and nonTE PAVs in the broomcorn millet population. a, Distribution of TE-derived and nonTE PAVs in the broomcorn millet genome; **b,** Numbers and insertion time of the top 20 abundant intact LTR retrotransposon (LTR-RT) families. Intact LTR-RTs were identified with LTR_retriever in Longmi4 and 32 assembled genomes. The top 20 families were consistent between Longmi4 and 32 assembled genomes. Number of intact LTR-RTs of Longmi4 was shown on the left. Insertion time of the corresponding family in 32 assembled genomes was shown on the right. Number of LTR elements that were partially overlapped (< 90%) with a PAV or entirely overlapped (≥ 90%) with a PAV were showed in orange and purple; **c,** Distribution of observed and expected (control) PAVs in domestication regions. Expected numbers of each PAV category were calculated based on the ratio of domestication regions to the whole genome; **d,** Allele frequency of DNA-transposon derived (DNA-TE) PAVs, LTR retrotransposon-derived (LTR-TE) PAVs, and nonTE PAVs in cultivated and wild populations. Colors indicate different level of significance measured as false discovery rate (FDR); **e,** Change of allele frequency of significantly altered PAVs between the cultivated and wild populations (two-sided fisher exact test, FDR ≤ 0.001); **f,** Sequence comparison of BC328

and Longmi4 at the *longmi031277* locus. The haplotype with a TE insertion in *longmi031277* was Ref, while the haplotype without the insertion is Alt. The frequency of Alt allele in C1, C2, C3, and W1 populations was shown in pie plots; **g,** Gene expression of *longmi031277* of 6 Alt and 26 Ref accessions with 3 biological independent experiments in leaf tissues of broomcorn millet. Significant level was determined by two-sided Wilcoxon rank-sum test. Edges and centerlines of the boxes represent the interquartile ranges and the medians, with whiskers extending to most extreme points (1.5 × IQR); **h,** GWAS signal of grain weight, π, F_{ST}, and XP-CLR at the *longmi031277* locus.

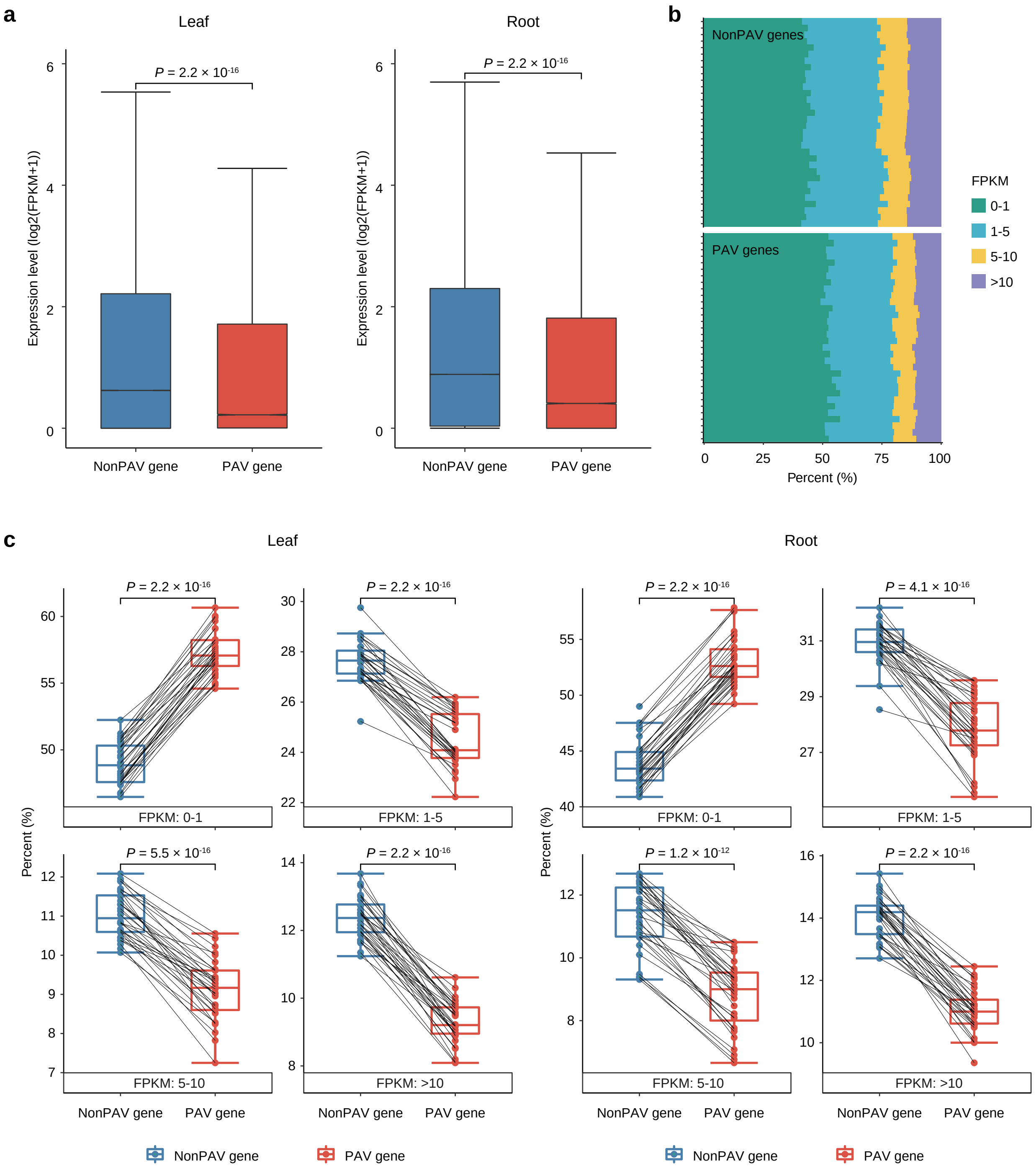

Supplementary Fig. 16. Comparison of PAV and nonPAV gene expression levels in 32 broomcorn millet accessions. a, Expressions of 72,513 PAV genes and 1,710,962 nonPAV genes of 32 accessions in leaf and root tissues; **b,** Comparison of gene expressions between PAV genes and nonPAV genes in root tissues of 32 accessions; **c,** Comparisons of proportion of PAV genes and nonPAV genes in each expression category in leaf and root tissues. Each point represents an accession. Gene expressions (FPKM) were indicated in each comparison, including 0-1, 1-5, 5-10, and >10. In **a** and **c**, significant level was determined by two-sided Wilcoxon rank-sum test. Edges and centerlines of the boxes represent the interquartile ranges and the medians, with whiskers extending to most extreme points (1.5 × IQR). In **c**, the black lines indicate the same accessions.

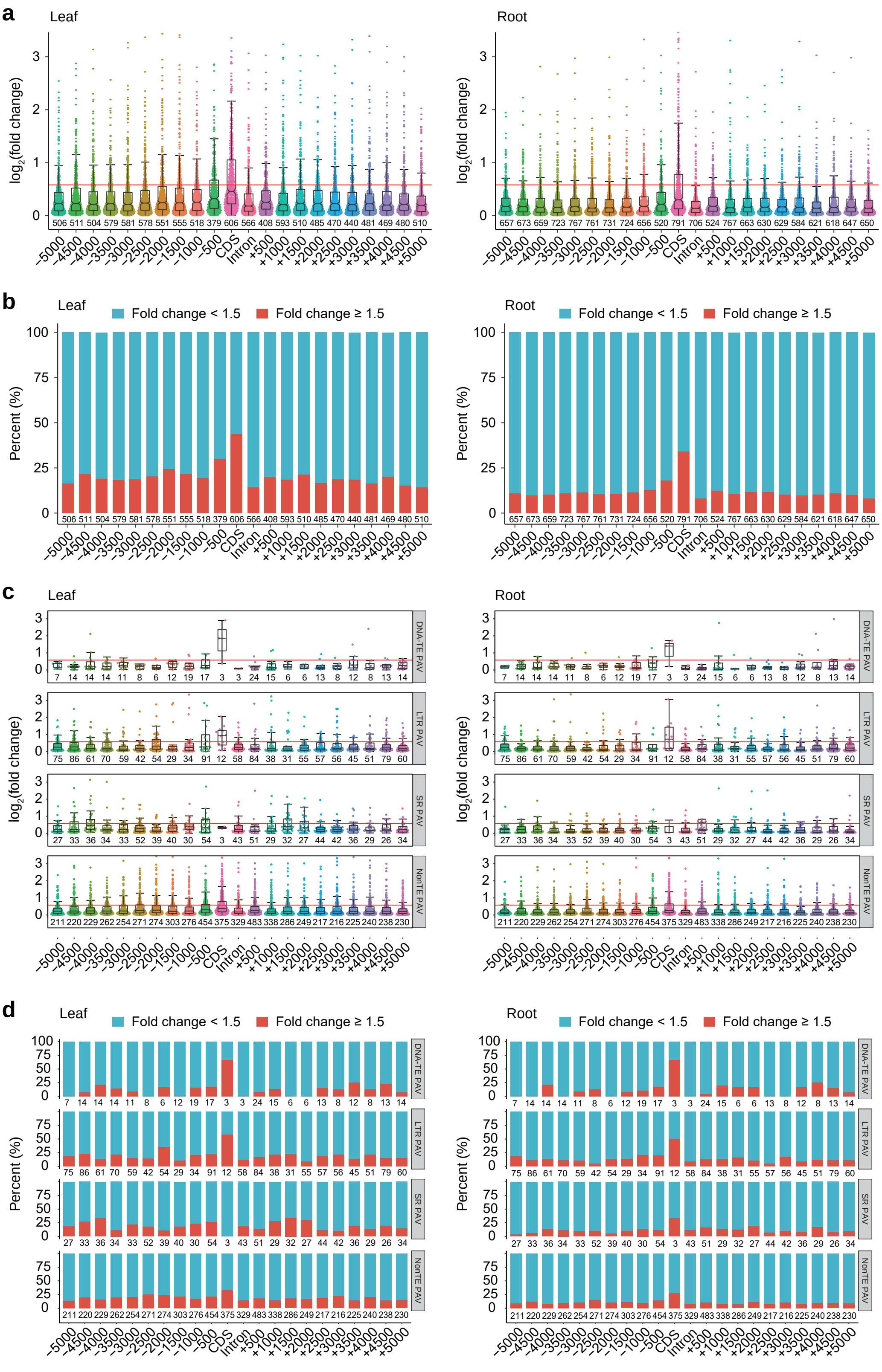

Supplementary Fig. 17. PAVs impact gene expression by affecting gene coding or regulatory sequences. a, Distribution of log_2(fold change) between genes with or without PAVs in leaf and root tissues; **b,** Composition of gene expression changes between genes with or without PAVs in leaf and root tissues; **c,** Distribution of log_2(fold change) between genes with or without different categories of PAVs in leaf and root tissues; **d,** Composition of gene expression changes between genes with or without different categories of PAVs in leaf and root tissues. "-" represents upstream of start codon; "+" represents downstream of stop codon. Number under each bar represents the number of genes. In **a** and **c**, edges and centerlines of the boxes represent the interquartile ranges and the medians, with whiskers extending to most extreme points (1.5 × IQR).

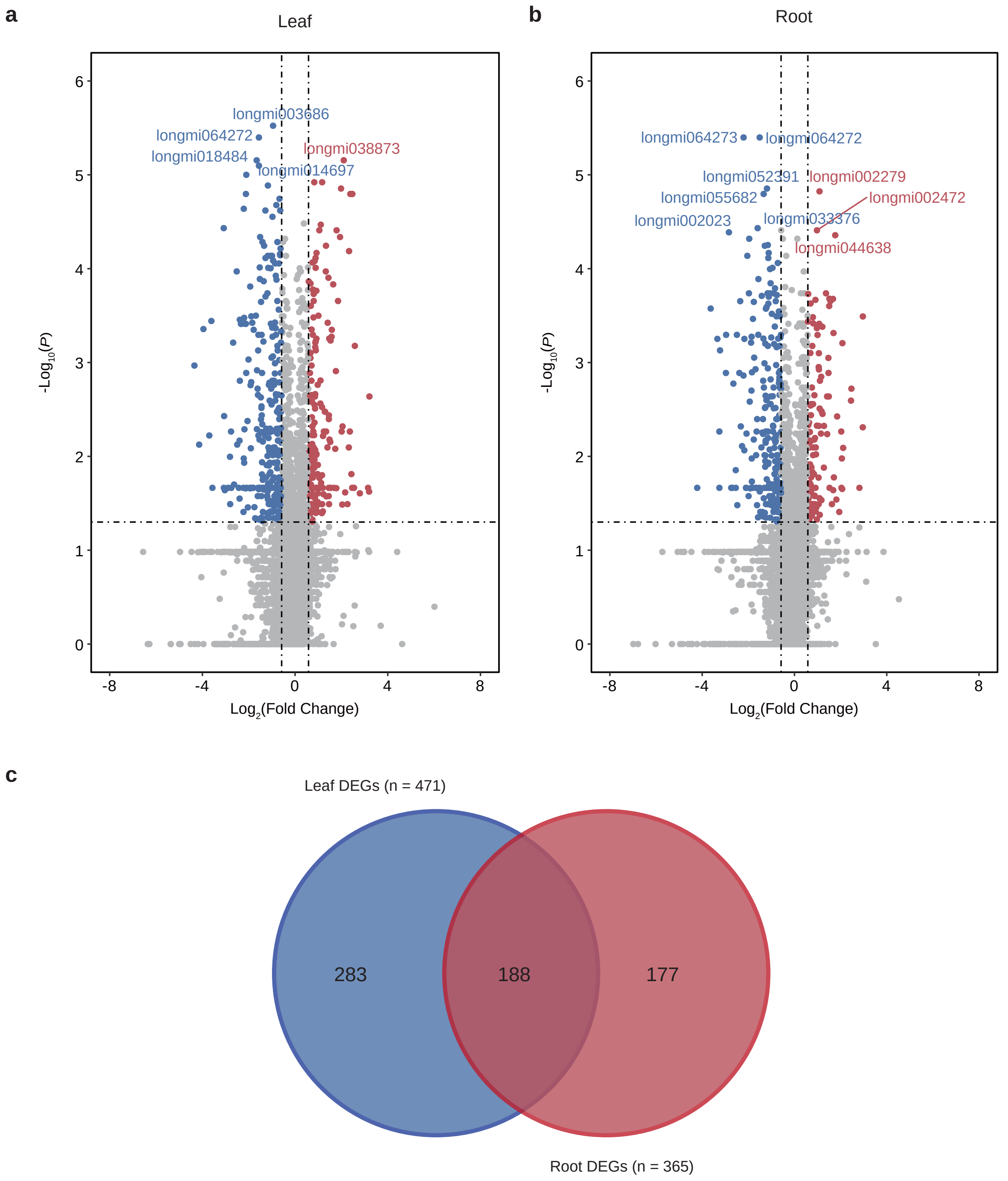

Supplementary Fig. 18. Differential expression analysis of PAV and nonPAV genes. a, Volcano plot of differential expression genes of leaf; **b,** Volcano plot of differential expression genes of root. Differential expressed significantly levels were determined by two-sided Wilcoxon rank-sum test between accessions with PAV and without PAV. Adjusted *P* value ≤ 0.05 and fold change ≥ 1.5 were used as threshold for significance; **c,** Number of differential expression genes in leaf and root tissues.

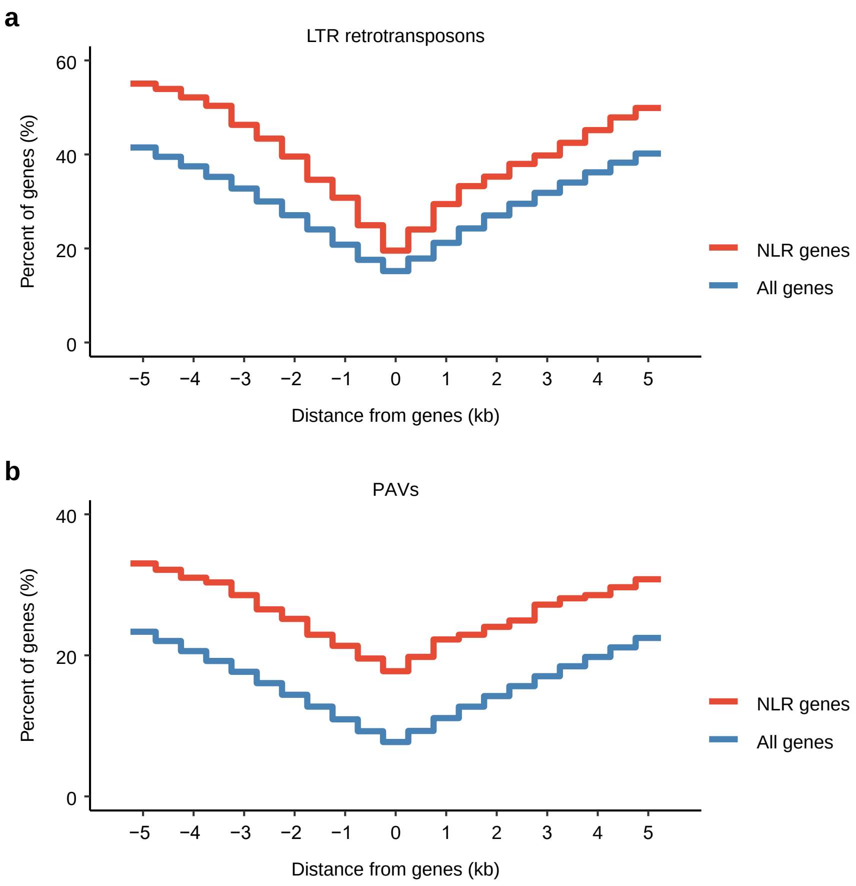

Supplementary Fig. 19. Distribution of LTR retrotransposons (a) and PAVs (b) at the nucleotide-binding domain and leucine-rich repeat (NLR) genes. On the *x*-axis, 0 represents LTRs or PAVs overlapping with gene bodies, while negative and positive values represent LTRs or PAVs locating at upstream of start codon or downstream of stop codon, respectively. The *y*-axis represents the percentage of genes overlapping with LTRs or PAVs. NLR genes refers to genes that contain the nucleotide-binding domain (PF00931).

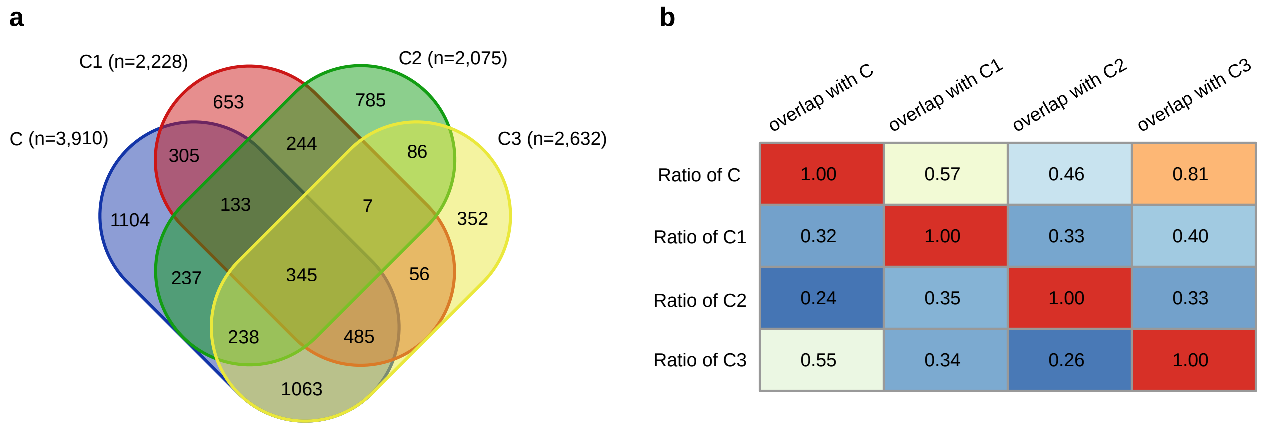

Supplementary Fig. 20. Protein-coding genes in the selective regions of three cultivated populations. a, Venn diagram of domestication genes of C1, C2, and C3 populations and combined cultivated population (C); **b,** Proportion of overlapped domestication genes in each population.

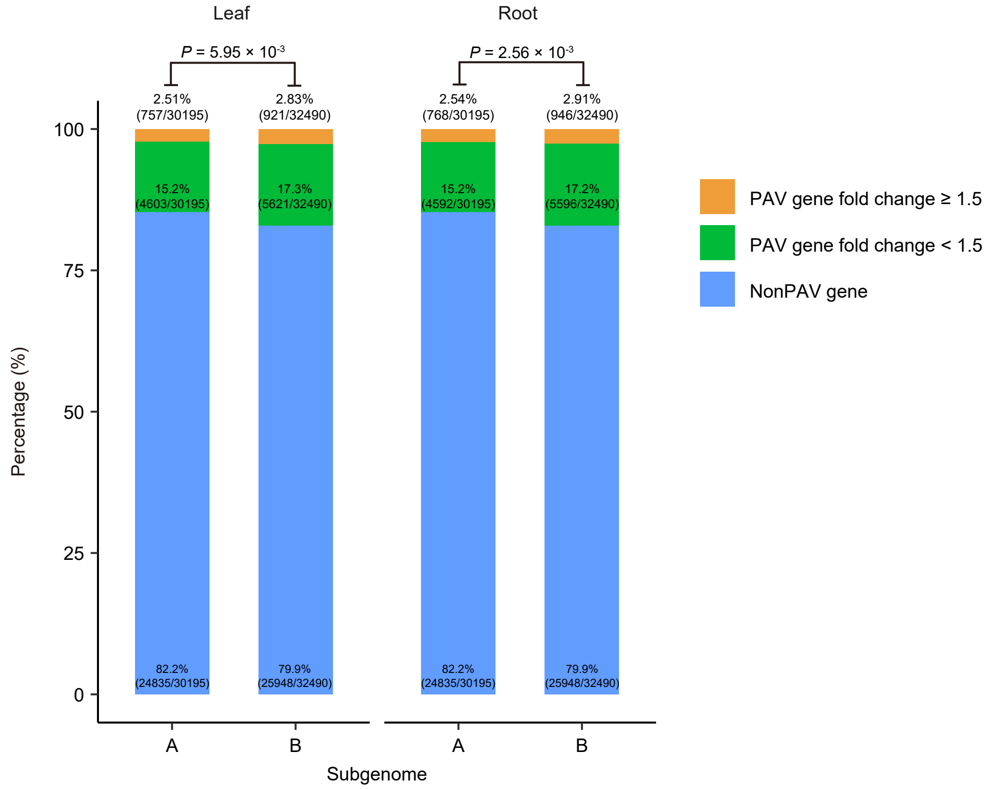

Supplementary Fig. 21. Differences in the expression of PAV and nonPAV genes in two subgenomes of broomcorn millet. A and B represent the subgenomes of broomcorn millet. Proportion of differential expressed PAV genes (fold change ≥ 1.5) between subgenome A and B was compared by two-sided Z-test..

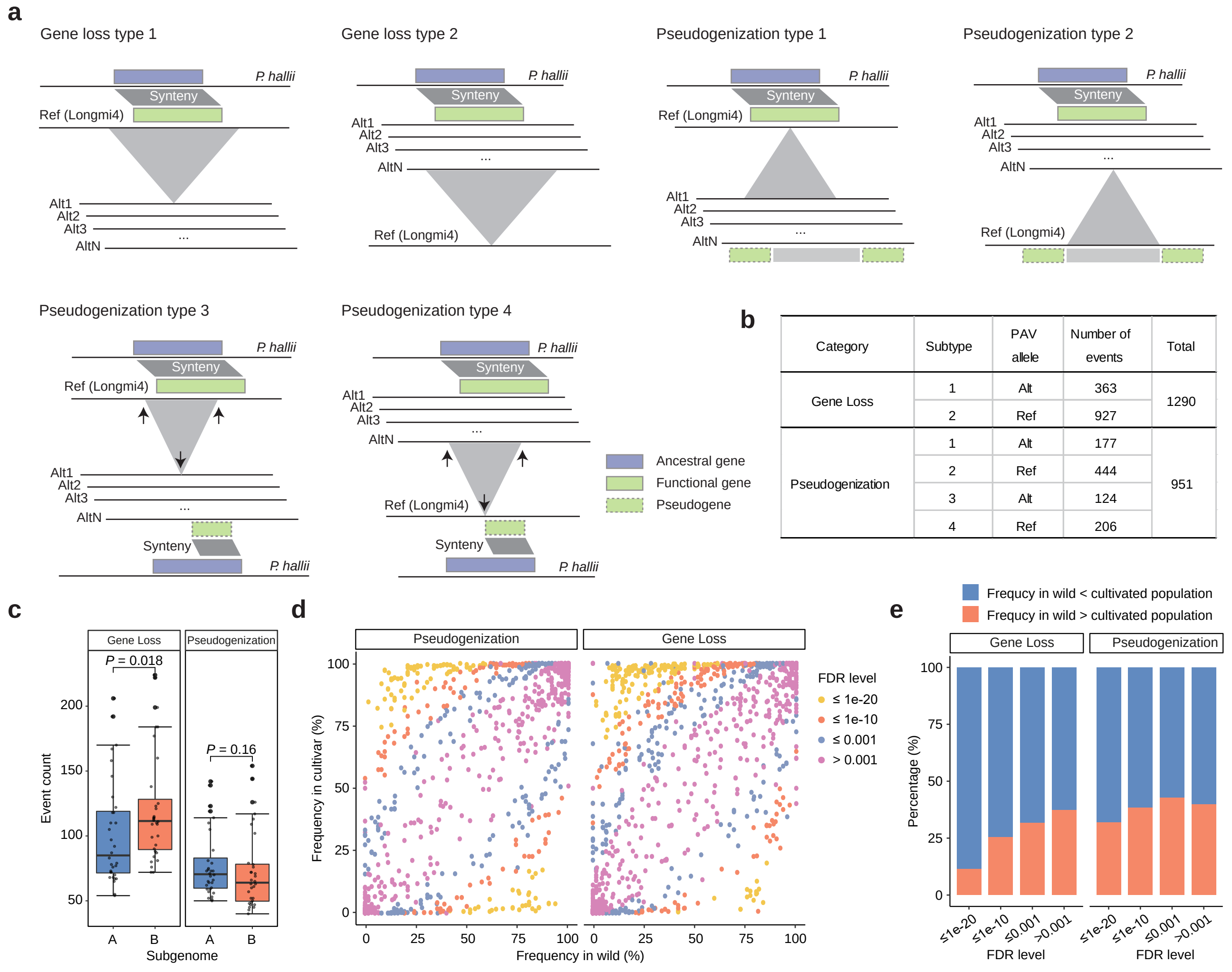

Category	Subtype	PAV allele	Number of events	Total
Gene Loss	1	Alt	363	1290
	2	Ref	927	
Pseudogenization	1	Alt	177	951
	2	Ref	444	
	3	Alt	124	
	4	Ref	206	

Supplementary Fig. 22. Analysis of gene loss and pseudogenization associated with PAVs in broomcorn millet. a, Schematic diagram of gene loss and pseudogenization subtypes. We defined ancestral genes as genes conserved in broomcorn millet, *P. hallii*, and switchgrass. Gene loss type 1 indicates that an ancestral gene of Ref allele is disrupted by a deletion; Gene loss type 2 indicates that an ancestral gene of Alt allele is disrupted by deletion; Pseudogenization type 1 indicates that an insertion disrupted an ancestral gene in Ref allele; Pseudogenization type 2 indicates that an insertion disrupted an ancestral gene in Alt allele; Pseudogenization type 3 indicates that an ancestral gene in Ref allele is truncated by a deletion; Pseudogenization type 4 indicates that an ancestral gene in Alt allele is truncated by a deletion; **b,** Count of gene loss and pseudogenization events associated with PAVs in broomcorn millet; **c,** The number of gene loss and pseudogenization events in two subgenomes of broomcorn millet. Statistical significance was calculated by two-sided Wilcoxon rank-sum test based on event presence in 32 genomes (n = 32). Edges and centerlines of the boxes represent the interquartile ranges and the medians, with whiskers extending to most extreme points (1.5 × IQR); **d,** Allele frequency of PAVs associated with gene loss and pseudogenization events. The significance of frequency differences for each event between wild and cultivated population was determined using two-sided Fisher's exact test. *P* values of all events were corrected using false discovery rate (FDR); **e,** Proportion of gene loss and pseudogenization events with frequency changes between wild and cultivated populations (FDR ≤ 0.001).

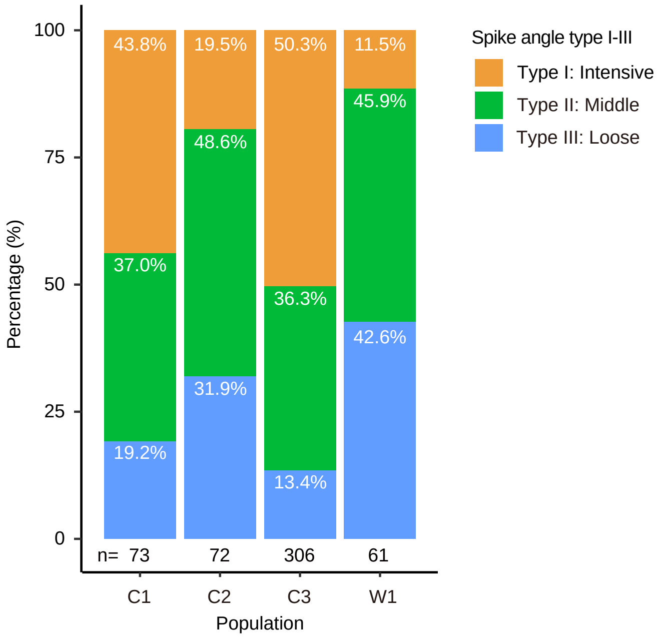

Supplementary Fig. 23. Phenotypic variation in the angle between panicle branch and spindle in C1, C2, C3, and W1 populations. N numbers on the *x*-axis represent the numbers of corresponding accessions.

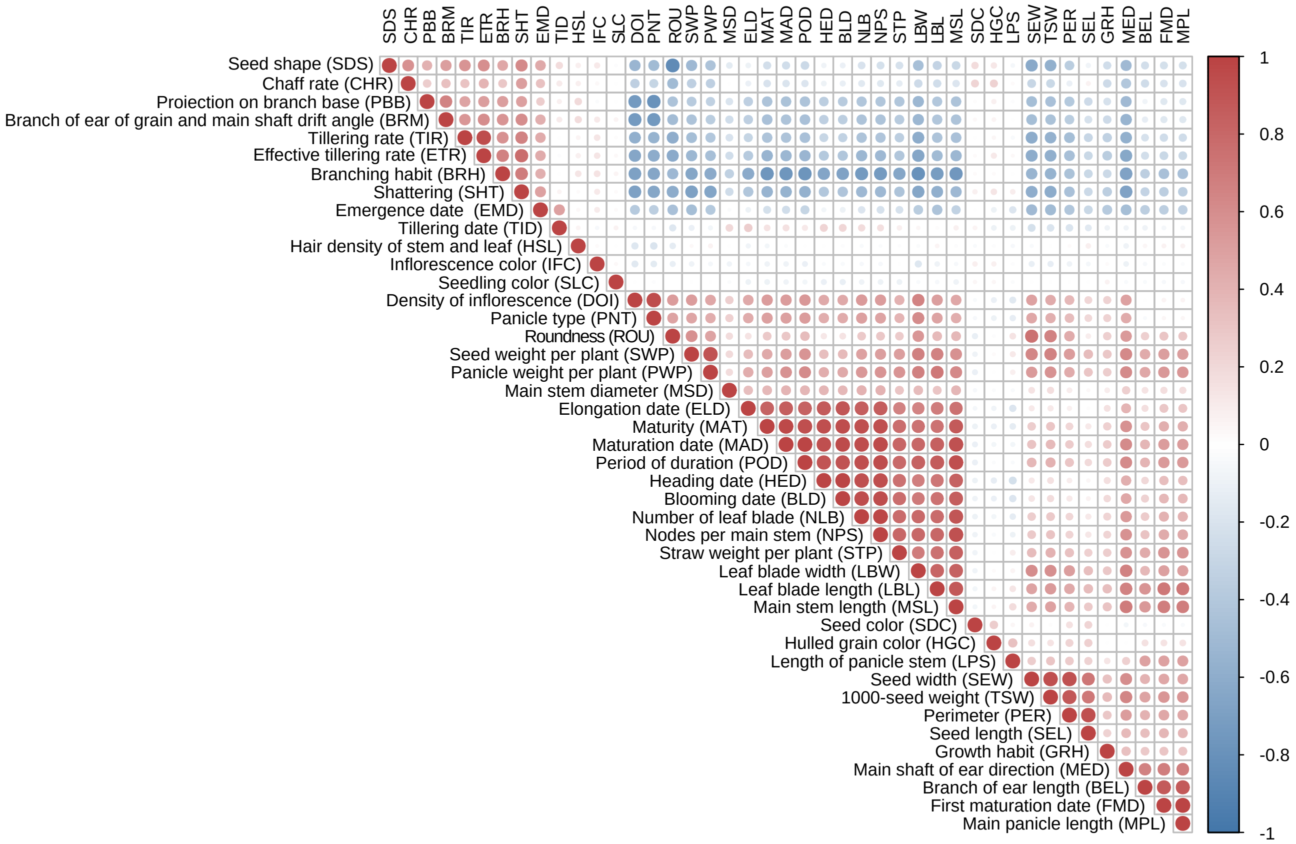

Supplementary Fig. 24. Correlation of 43 traits collected in 2019 and 2020. Best linear unbiased estimate (BLUE) values of phenotypic data were calculated using R package lme4. Correlation coefficients among traits based on BLUE values were calculated using pearson correlation by R package Corrplot. Circles show *R* and *P* value of correlation with significance threshold at $P < 0.05$. Size of circles indicate *P* value and colors indicate *R* of correlation.

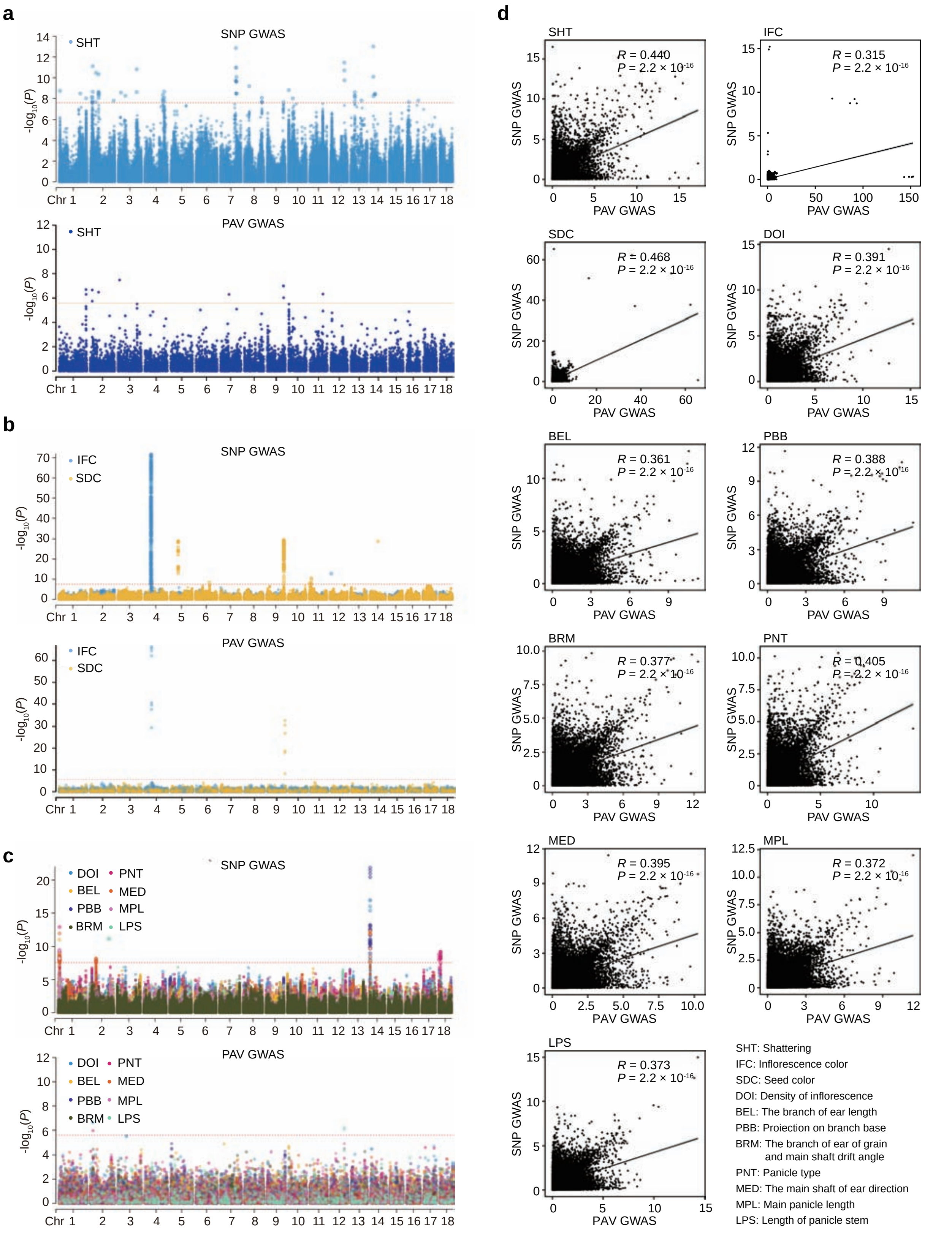

Supplementary Fig. 25. Comparison between SNP-GWAS and PAV-GWAS. a, The Manhattan plots of SNP- and PAV-GWAS results from analysis of shattering (SHT); **b,** The Manhattan plots of SNP- and PAV-GWAS results from analysis of inflorescence color (IFC) and seed color (SEC); **c,** The Manhattan plots of SNP- and PAV-GWAS results from analysis of density of inflorescence (DOI), the branch of ear length (BEL), projection on branch base (PBB), the branch of ear of grain and main shaft drift angle (BRM), panicle type (PNT), the main shaft of ear direction (MED), main panicle length (MPL), and length of panicle stem (LPS). For **a-c**, the horizontal lines depict the threshold for GWAS ($P = 2.64 \times 10^{-8}$ or $-\log_{10}(P) = 7.58$ for SNP-GWAS and $P = 2.57 \times 10^{-6}$ or $-\log_{10}(P) = 5.59$ for PAV-GWAS). GWAS significance

thresholds were set at 0.05 / total number of SNPs or PAVs; **d,** Correlation analyses of signals from SNP- and PAV-GWAS analyses of shattering (SHT), inflorescence color (IFC), seed color (SDC) and panicle traits (DOI, BEL, PBB, BRM, PNT, MED, MPL, LPS). Pearson's correlation coefficient (*R*) and *P* value was calculated with R function cor.test. Black lines indicate fitted curves for linear regression.

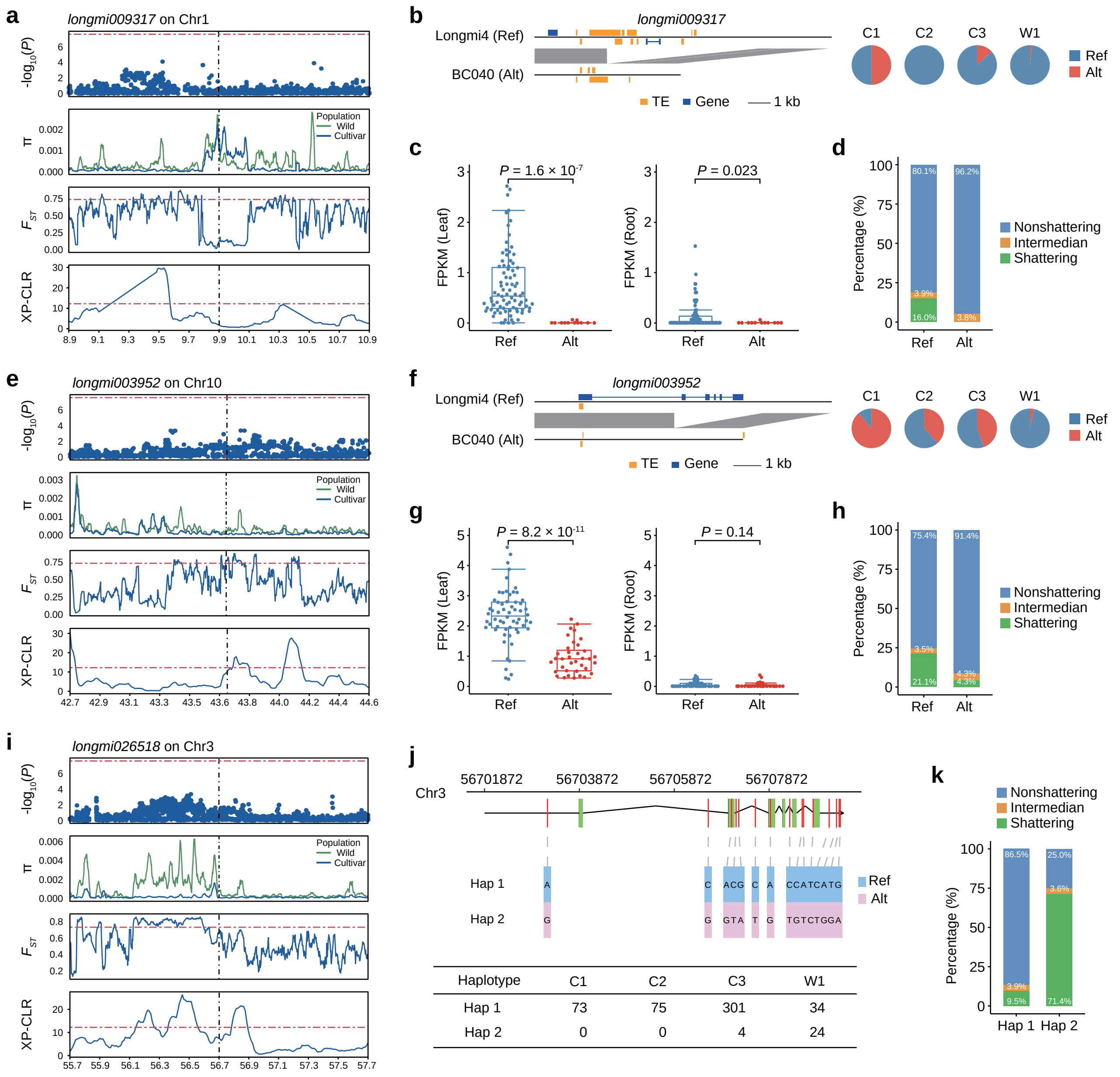

Haplotype	C1	C2	C3	W1
Hap 1	73	75	301	34
Hap 2	0	0	4	24

Supplementary Fig. 26. Analysis of homologous genes of *OsSh1* in broomcorn millet. a, GWAS signal, π, F_{ST}, and XP-CLR of *longmi009317* (the ortholog of *OsSh1*) in its nearby 1-Mb region; **b,** A PAV deleted *longmi009317* in BC040. Pie plots show the PAV frequency in C1, C2, C3, and W1 populations; **c,** Expression of *longmi009317* in 4 accessions with PAV (Alt) and 28 accessions without PAV (Ref) in leaf (left) and root (right) tissues, and each accession has three biological independent experiments; **d,** Shattering phenotype in accessions with (n = 78) or without (n = 438) PAVs; **e,** GWAS signal, π, F_{ST}, and XP-CLR of *longmi003952* (a close paralog of *OsSh1*) in its nearby 1-Mb region; **f,** A PAV at the gene body truncated *longmi003952*. Pie plots show the PAV frequency in C1, C2, C3, and W1 populations; **g,** Expression of *longmi003952* in 12 accessions with PAV (Alt) and 20 accessions without PAV (Ref) in leaf (left) and root (right) tissues, and each accession has three biological independent experiments; **h,** Shattering phenotype in accessions with (n = 232) or without (n = 284) PAVs; **i,** GWAS signal, π, F_{ST}, and XP-CLR of *longmi026518* in its nearby 1-Mb region; **j,** Distribution of the two haplotypes of *longmi026518* in C1, C2, C3, and W1 populations; **k,** Shattering phenotype of accessions with Hap 1 (n = 483) and Hap 2 (n = 28). In **c** and **g**, significant levels of differential expression were determined by two-sided Wilcoxon rank-sum test. Edges and centerlines of the boxes represent the interquartile ranges and the medians, with whiskers extending to most extreme points (1.5 × IQR).

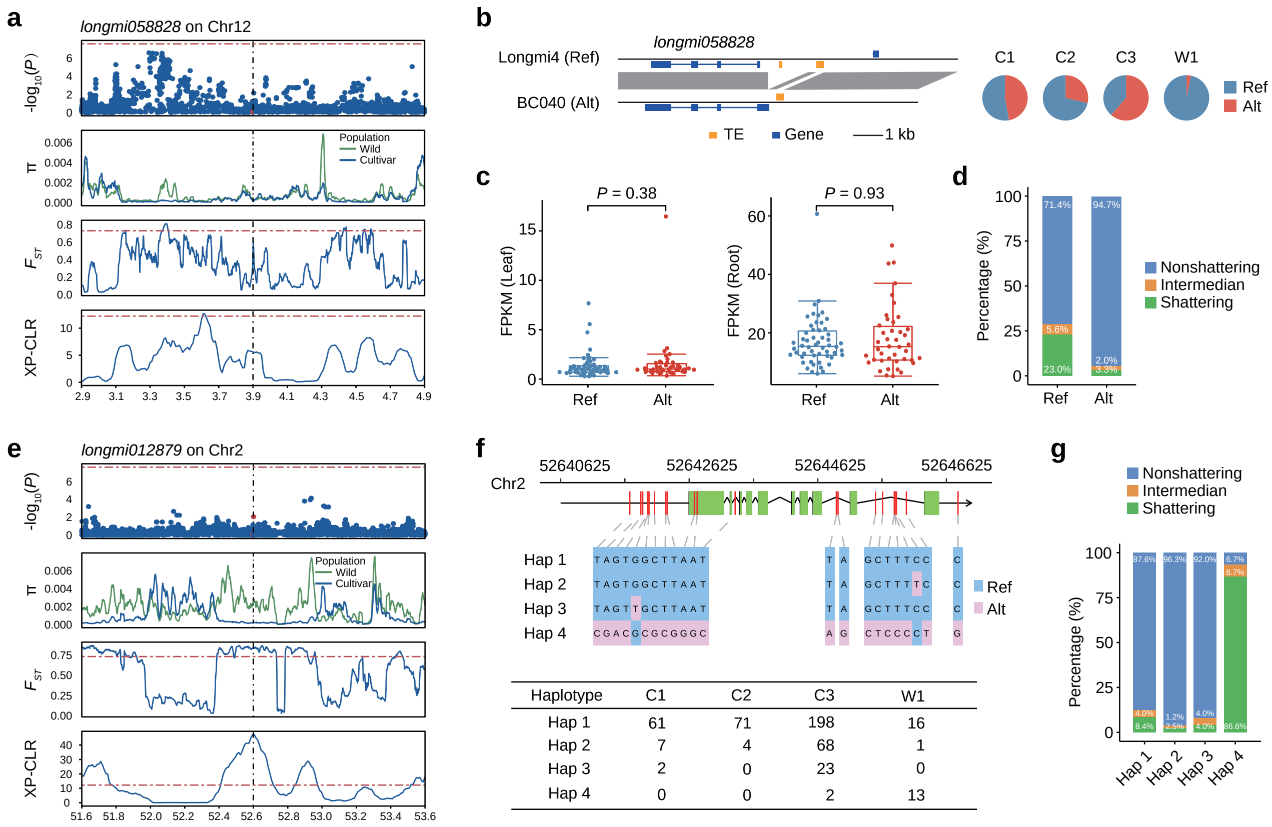

Supplementary Fig. 27. Analysis of ortholog of *OsCAD2* and *SSH1/OsSNB* in broomcorn millet. a, GWAS signal, π, F_{ST}, and XP-CLR of *longmi058828* (the ortholog of *OsCAD2*) in its nearby 1-Mb region; **b,** Distribution of PAVs of *longmi058828* in C1, C2, C3, and W1 populations; **c,** Expression of *longmi058828* in 14 accessions with PAV (Alt) and 18 accessions without PAV (Ref) in leaf (left) and root (right) tissues, and each accession has three biological independent experiments; **d,** Shattering phenotype in accessions with (Alt; n = 269) or without (Ref; n = 247) PAVs; **e,** GWAS signal, π, F_{ST}, and XP-CLR of *longmi012879* (the ortholog of *SSH1/OsSNB*) in its nearby 1-Mb region; **f,** Distribution of the four haplotypes of *longmi012879* in C1, C2, C3, and W1 populations; **g,** Shattering phenotype in accessions of Hap 1, 2, 3, and 4 haplotypes with 346, 80, 25, and 15 individuals, respectively. In **c**, significant levels of differential expression were determined by two-sided Wilcoxon test. Edges and centerlines of the boxes represent the interquartile ranges and the medians, with whiskers extending to most extreme points (1.5 × IQR).

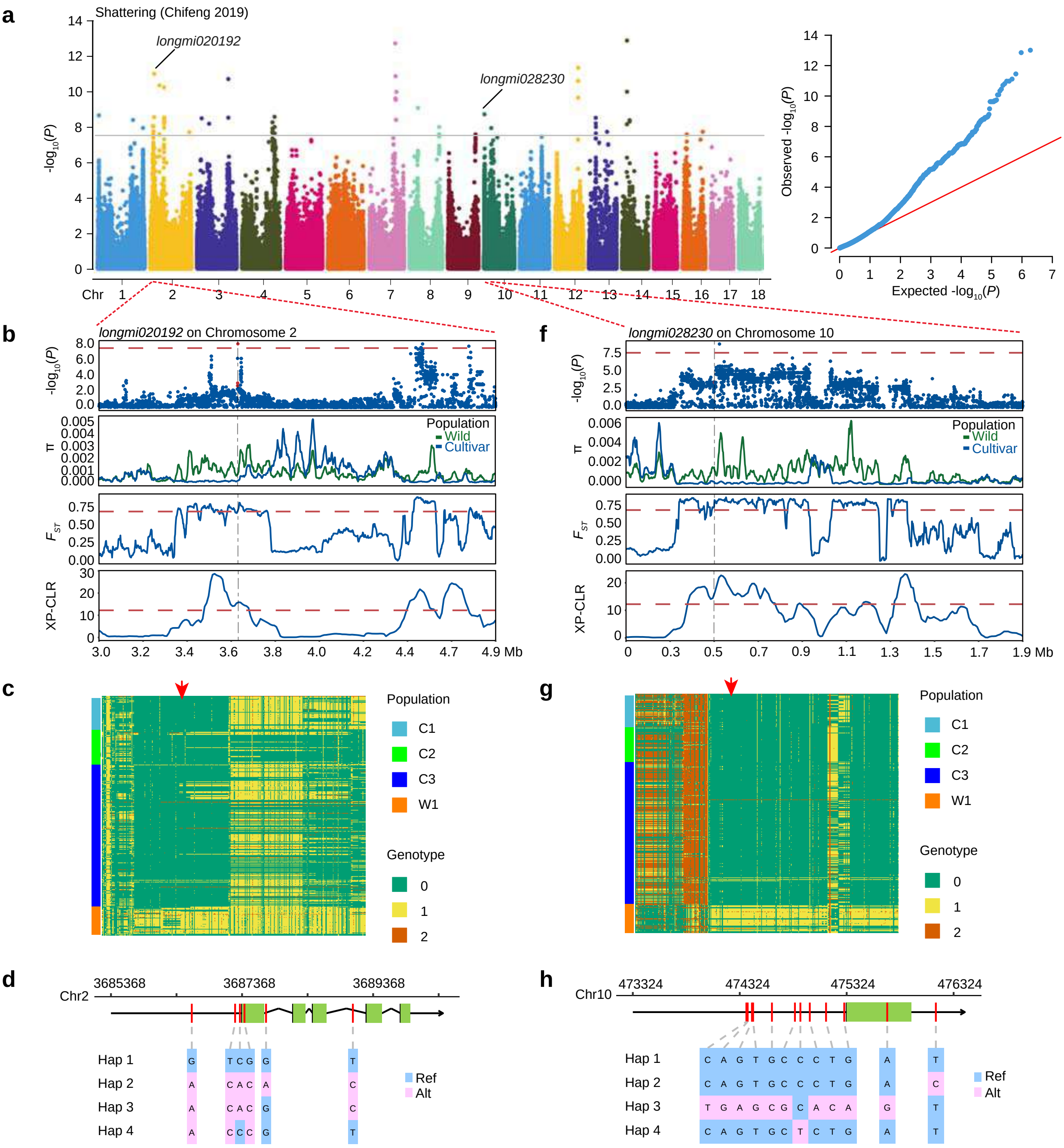

e

Haplotype	C1	C2	C3	W1
Hap 1	73	70	291	12
Hap 2	0	0	2	24
Hap 3	0	0	0	15
Hap 4	0	2	10	0

i

Haplotype	C1	C2	C3	W1
Hap 1	47	67	191	13
Hap 2	16	1	82	0
Hap 3	0	0	3	46
Hap 4	6	6	25	0

Supplementary Fig. 28. Analysis of candidate genes for shattering in broomcorn millet. a, The Manhattan plot of GWAS result from analysis of data for shattering; **b,** Local Manhattan plot of chromosome 2 obtained from GWAS for the shattering (top) and signatures of artificial selection at the *longmi020192* locus evaluated by π, F_{ST}, and XP-CLR (bottom); **c,** Haplotypes of the region surrounding *longmi020192* in the broomcorn millet population. The red arrow indicates the location of *longmi020192*. The colored bars on the left represent C1, C2, C3, and W1 populations; **d,** Six SNPs composed four major haplotypes located near *longmi020192*; **e,** Distribution of the four haplotypes surrounding *longmi020192* in C1, C2, C3, and W1 populations; **f,** Local Manhattan plot of chromosome 10 obtained from GWAS for the shattering (top) and signatures of artificial selection at the *longmi028230* locus evaluated by π, F_{ST}, and XP-CLR; **g,** Haplotypes of the region surrounding *longmi028230* in the broomcorn millet population. The red arrow indicates the location of *longmi028230*. The colored bars on the left represent C1, C2, C3, and W1 populations; **h,** Twelve SNPs

composed four major haplotypes located near *longmi028230*; **i,** Distribution of the four haplotypes surrounding *longmi028230* in C1, C2, C3, and W1 populations. In **a**, **b**, and **f**, the GWAS significance threshold was set at 0.05 / total number of SNPs ($P = 2.64 \times 10^{-8}$ or $-\log_{10}(P) = 7.58$).

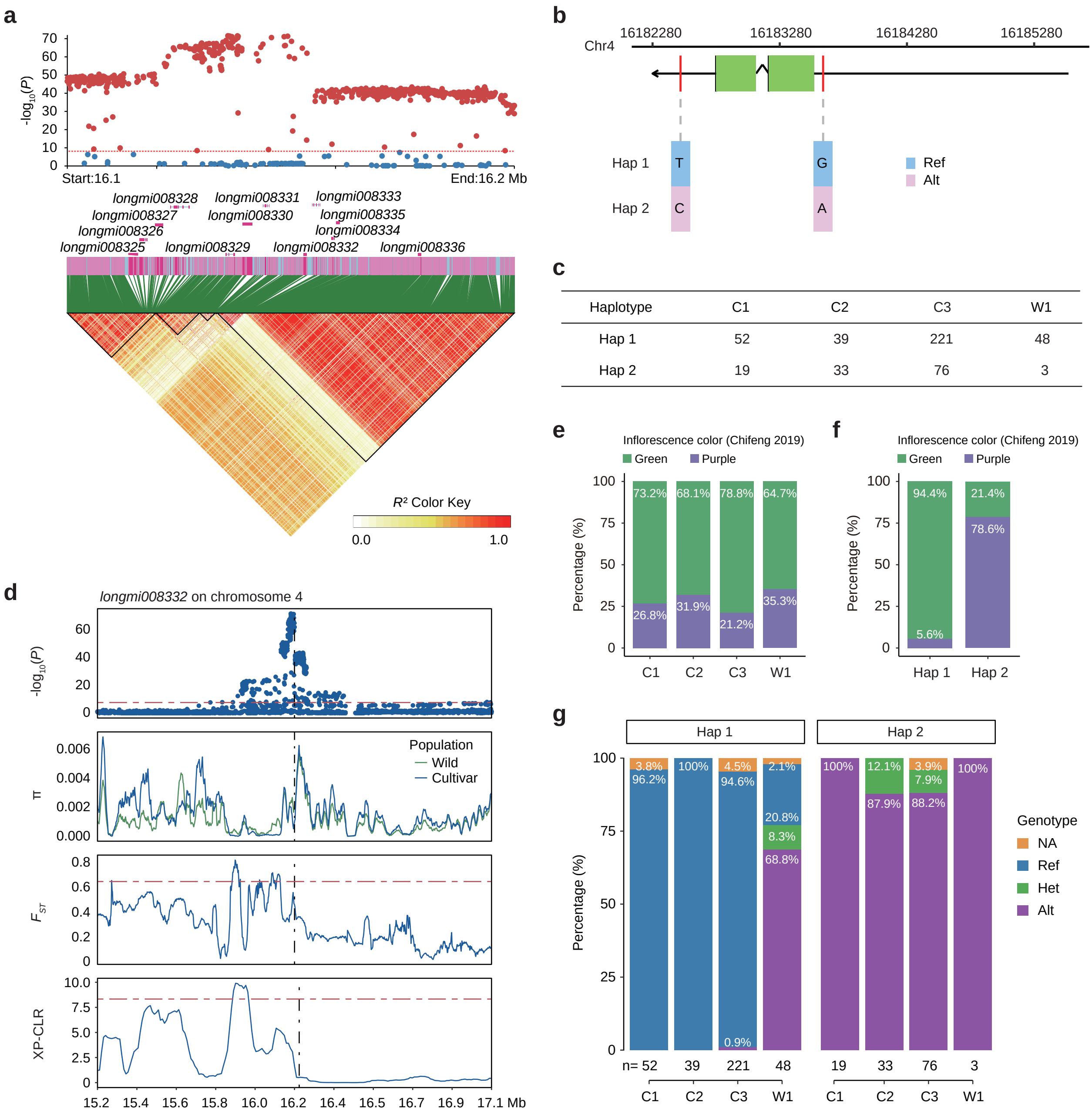

Supplementary Fig. 29. GWAS analysis of the inflorescence color candidate gene *longmi008332*. a, Local Manhattan plot of chromosome 4 obtained from GWAS for inflorescence color (top) and heatmap of LD surrounding the GWAS signals (bottom). LD blocks are highlighted in black triangles. The color key indicates R^2. The red horizontal line depicts the threshold for GWAS; **b,** Two major haplotypes of longmi008332; **c**, Occurrences of two haplotypes in C1, C2, C3, and W1 populations; **d,** GWAS signal and signatures of artificial selection at the *longmi008332* locus evaluated by π, F_{ST}, and XP-CLR; **e,** Inflorescence color of accessions in C1 (n = 71), C2 (n = 72), C3 (n = 297), and W1 (n = 51) populations; **f,** Inflorescence color of accessions of Hap 1 (n = 369) and Hap2 (n = 131); **g,** Comparison of PAV genotypes and SNP-haplotypes in C1, C2, C3, and W1 populations. In **a** and **d**, the GWAS significance threshold was set at 0.05 / total number of SNPs ($P = 2.64 \times 10^{-8}$ or $-\log_{10}(P) = 7.58$).

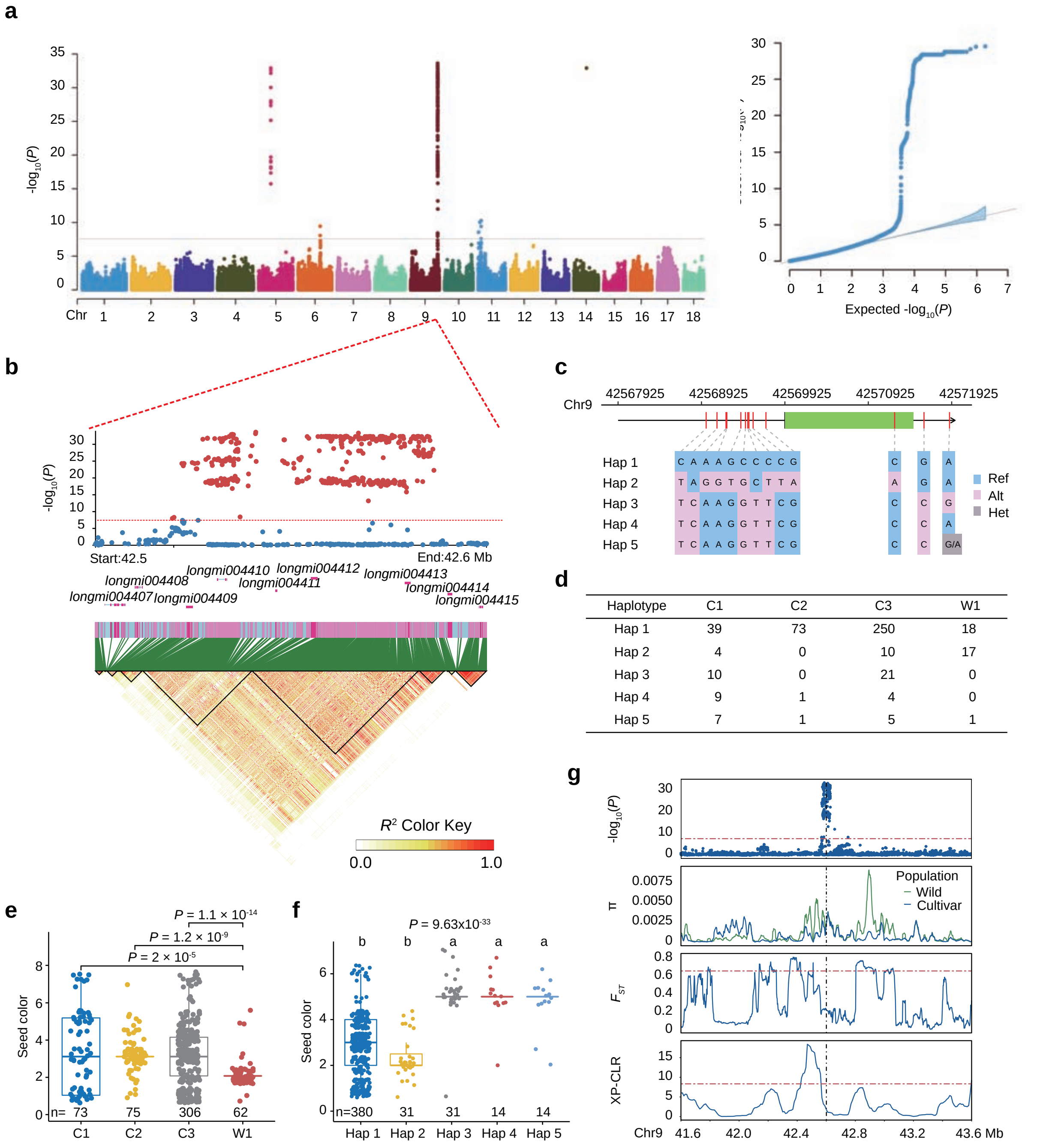

Haplotype	C1	C2	C3	W1
Hap 1	39	73	250	18
Hap 2	4	0	10	17
Hap 3	10	0	21	0
Hap 4	9	1	4	0
Hap 5	7	1	5	1

Supplementary Fig. 30. GWAS analysis of the seed color candidate genes *longmi004409*, *longmi004412*, and *longmi004413*. a, The Manhattan plot of GWAS result from analysis of data for seed color; **b,** Local Manhattan plot of chromosome 9 obtained from GWAS for the seed color (top) and heatmap of LD surrounding the GWAS signals (bottom). LD blocks are highlighted in black triangles. The color key indicates R^2; **c,** five major haplotypes located on *longmi004412*; **d,** Distribution of the five haplotypes in C1, C2, C3, and W1 populations; **e,** Seed color of accessions in C1, C2, C3, and W1 populations. Significant levels were determined by two-sided Wilcoxon rank-sum test between C1/C2/C3 and W1 population; **f,** Seed color of accessions with different haplotypes. The values of *y*-axis indicate seed color categories. One-way ANOVA test was used to determine differences among groups. Pairwise comparison was conducted by the least significant difference (LSD) method with Bonferroni correction for multiple comparisons. Different lowercase letters above the box plots represent significant phenotype differences ($P \leq 0.05$); **g,** GWAS signal and the signatures of artificial selection at the *longmi004412* locus evaluated by π, F_{ST}, and XP-CLR. In **a**, **b**, and **g**, the red horizontal line depicts the threshold for GWAS ($P = 2.64 \times 10^{-8}$ or $-\log_{10}(P) = 7.58$), which was set at 0.05 / total number of SNPs. In **e** and **f**, edges and centerlines of the boxes represent the interquartile ranges and the medians, with whiskers extending to most extreme points (1.5 × IQR). N numbers on the *x*-axis represent the number of samples in corresponding categories.

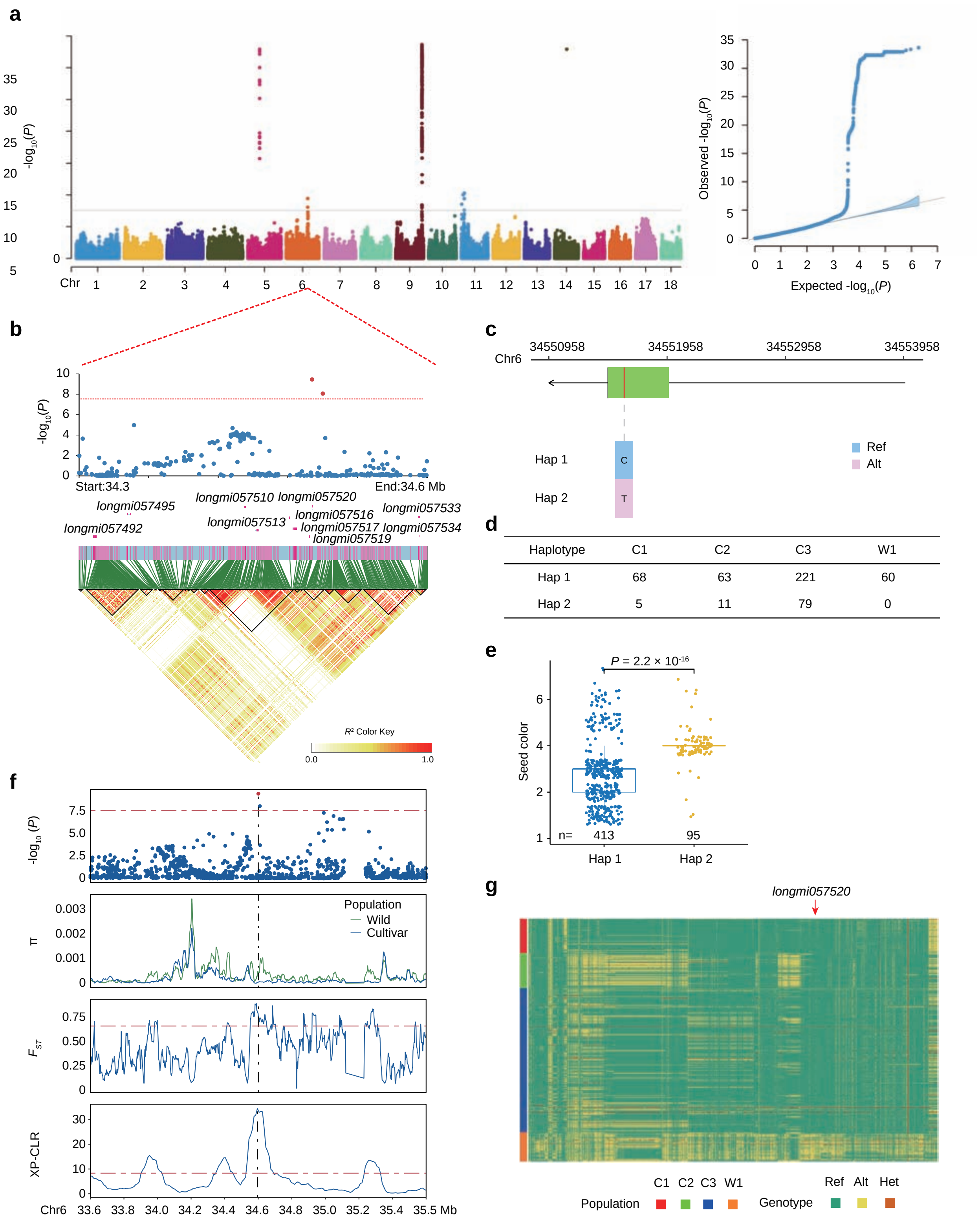

Supplementary Fig. 31. GWAS analysis of the seed color candidate gene *longmi057520*. a, The Manhattan plot of GWAS result from analysis of data for seed color; **b,** Local Manhattan plot of chromosome 6 obtained from GWAS for the seed color (top) and heatmap of LD surrounding the GWAS signals (bottom). LD blocks are highlighted in black triangles. The color key indicates R^2; **c,** A SNP (S6_34551595, C/T) located on chromosome 6 lead to a premature stop codon of *longmi057520*; **d,** Distribution of the two haplotypes in C1, C2, C3, and W1 populations; **e,** Seed color of accessions with different haplotypes. The values of *y*-axis indicate seed color categories. N numbers on the *x*-axis represent the number of samples in corresponding categories. Significantly level was determined by two-sided Wilcoxon rank-sum test. Edges and centerlines of the boxes represent the interquartile ranges and the medians, with whiskers extending to most extreme points (1.5 × IQR); **f,** Signatures of artificial selection at the *longmi057520* locus evaluated by

π, F_{ST}, and XP-CLR; **g,** Haplotypes of the region surrounding *longmi057520* in the broomcorn millet population. The red arrow indicates the location of *longmi057520*. The colored bars on the left represent C1, C2, C3, and W1 populations. In **a**, **b**, and **f**, the threshold for GWAS ($P = 2.64 \times 10^{-8}$ or $-\log_{10}(P) = 7.58$) was set at 0.05 / total number of SNPs.

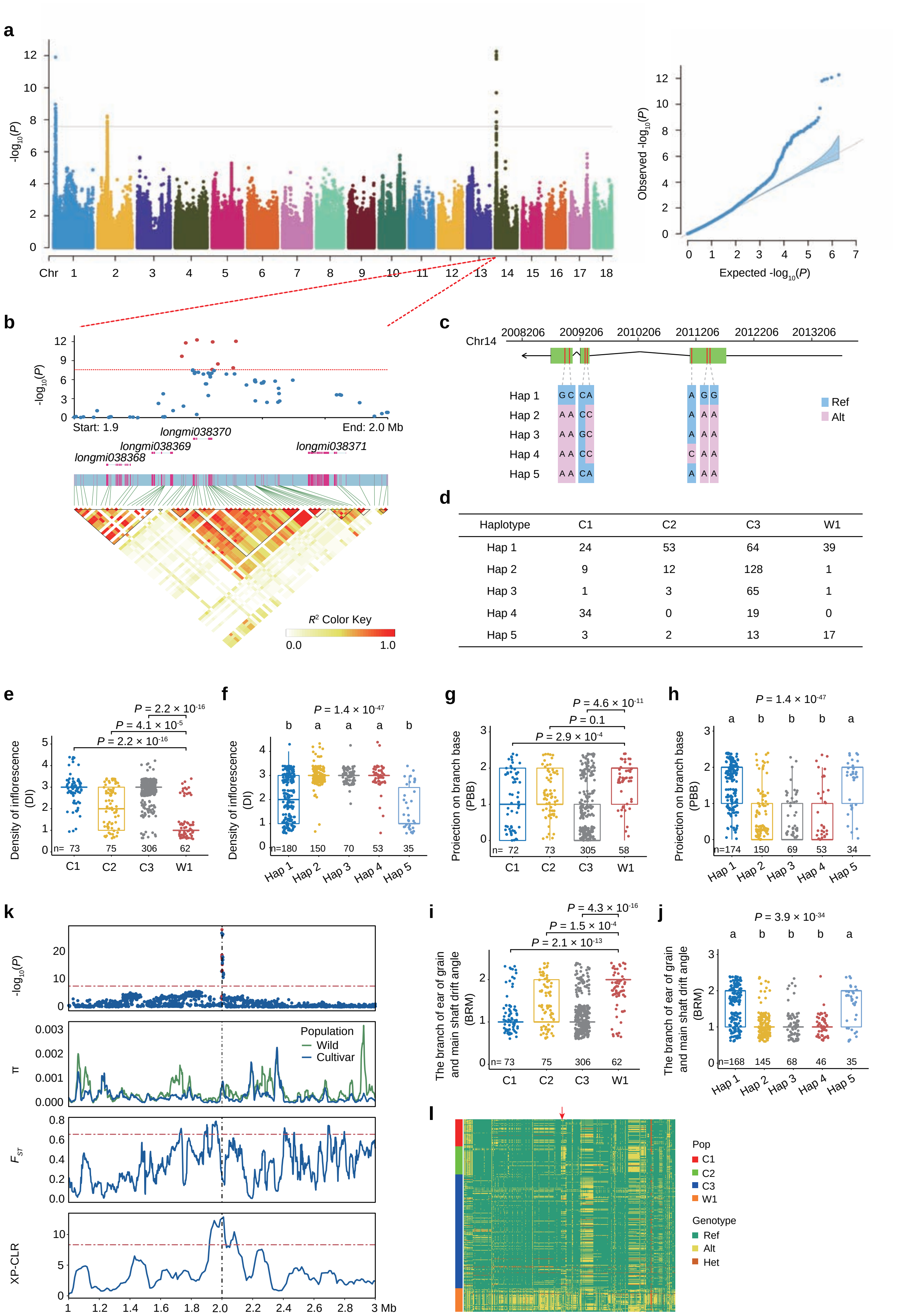

Haplotype	C1	C2	C3	W1
Hap 1	24	53	64	39
Hap 2	9	12	128	1
Hap 3	1	3	65	1
Hap 4	34	0	19	0
Hap 5	3	2	13	17

Supplementary Fig. 32. GWAS analysis of the panicle type candidate gene *longmi038370*. a, The Manhattan plot of GWAS result from analysis of data for panicle type; **b,** Local Manhattan plots of chromosome 14 obtained from GWAS

for the panicle type (top) and heatmap of LD surrounding the GWAS signals (bottom). LD blocks are highlighted in black triangles. The color key indicates R^2; **c,** Seven nonsynonymous SNPs composed five major haplotypes located on *longmi038370*; **d,** Distribution of the five haplotypes in C1, C2, C3, and W1 populations; **e,** Density of inflorescence in C1, C2, C3, and W1 populations; **f,** Density of inflorescence in accessions with different haplotypes; **g,** Projection on branch base in C1, C2, C3, and W1 populations; **h,** Projection on branch base in accessions with different haplotypes; **i,** Branch of ear of grain and main shaft drift angle in C1, C2, C3, and W1 populations; **j,** Branch of ear of grain and main shaft drift angle in accessions with different haplotypes; **k,** Signatures of artificial selection at the *longmi038370* locus evaluated by π, F_{ST}, and XP-CLR; **l,** Haplotypes of the region surrounding *longmi038370* in the broomcorn millet population. The red arrow indicates the location of *longmi038370*. The colored bars on the left represent C1, C2, C3, and W1 populations. In **a**, **b**, and **k**, the red horizontal line depicts the threshold for GWAS (P = 2.64 × 10^{-8} or $-\log_{10}(P)$ = 7.58), which was set at 0.05 / total number of SNPs. In **e**-**j**, edges and centerlines of the boxes represent the interquartile ranges and the medians, with whiskers extending to most extreme points (1.5 × IQR). N numbers on the *x*-axis represent the number of samples in corresponding categories. In **e**, **g**, and **i**, significant levels were determined by two-sided Wilcoxon rank-sum test between C1/C2/C3 and W1 population. In **f**, **h**, and **j**, one-way ANOVA test was used to determine differences among groups. Pairwise comparison was conducted by the least significant difference (LSD) method with Bonferroni correction for multiple comparisons. Different lowercase letters above the box plots represent significant phenotype differences ($P \leq 0.05$).

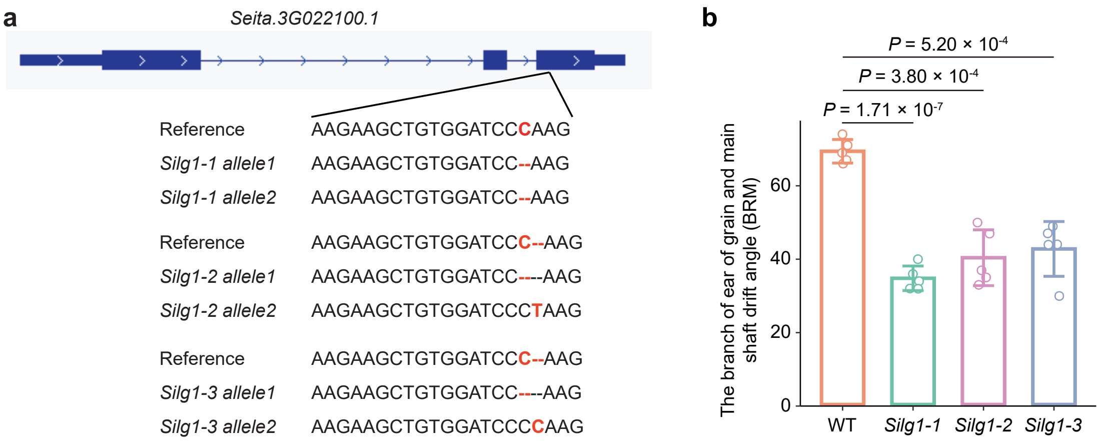

Supplementary Fig. 33. CRISPR mutation analysis of the orthologous gene (*Seita.3G022100.1*) of the panicle type candidate gene *longmi038370* in *Setaria italica*. a, Mutation analyses of three CRISPR mutants of *SiLG1* (*Seita.3G022100.1*, Sitalica_312_v2.2). Sequences of reference and mutated alleles were shown. Mutations and corresponding sequences were highlighted in red; **b,** Comparison of the branch of ear of grain and main shaft drift angle (BRM) in WT and three CRISPR mutants. Traits were collected from five plants in each line. *P* values were obtained from two-sided *t* test performed with R function t.test. BRMs of WT and three CRISPR mutants, each with five biological independent plants, were presented as mean ± s.d. in barplot.

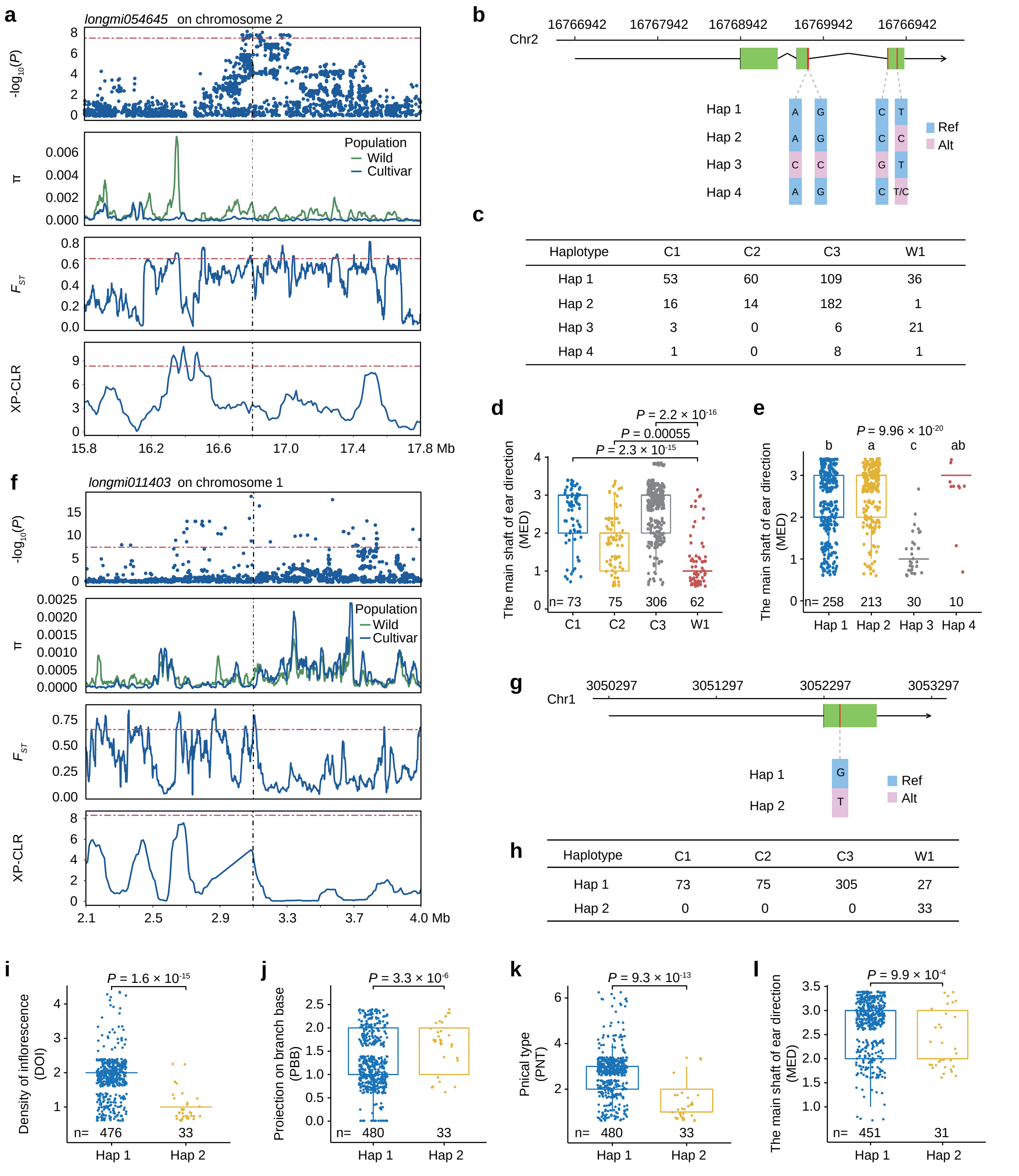

Supplementary Fig. 34. Haplotype analysis of the panicle type candidate genes *longmi011403* and *longmi054645*. a, GWAS signal and signatures of artificial selection at the *longmi054645* locus evaluated by π, F_{ST}, and XP-CLR; **b,** Four major haplotypes of *longmi054645*; **c,** Occurrences of four haplotypes in C1, C2, C3, and W1 populations; **d,** The main shaft of ear direction (MED) of accessions in C1, C2, C3, and W1 populations; **e,** The main shaft of ear direction (MED) of accessions with different haplotypes; **f,** GWAS signal and signatures of artificial selection at the *longmi011403* locus evaluated by π, F_{ST}, and XP-CLR; **g,** Two major haplotypes of *longmi011403*; **h,** Occurrences of two haplotypes in C1, C2, C3, and W1 populations; **i-l,** Density of inflorescence (DOI), projection on branch base (PBB), panicle type (PNT), and the main shaft of ear direction (MED) of accessions with different haplotypes. The values of *y*-axis indicate DOI, PBB, PNT, and MED categories. In **d**, **e**, **i**, **j**, **k**, and **l**, edges and centerlines of the boxes represent the interquartile ranges and the medians, with whiskers extending to most extreme points (1.5 × IQR). N numbers on the *x*-axis represent the number of samples in corresponding categories. In **d**, **i**, **j**, **k**, and **l**, significant levels of phenotype between different

populations and haplotypes were determined by two-sided Wilcoxon rank-sum tests. In **e**, one-way ANOVA test was used to determine differences among groups. Pairwise comparison was conducted by the least significant difference (LSD) method with Bonferroni correction for multiple comparisons. Different lowercase letters above the box plots represent significant phenotype differences ($P \leq 0.05$).
