# Supplementary material for: Pangenome analysis reveals genomic variations associated with domestication traits in broomcorn millet
Source: Nat Genet. 2023 Nov 30;55(12):2243–54. doi: 10.1038/s41588-023-01571-z (PMC10703678; doi:10.1038/s41588-023-01571-z)
Supplement: Supplementary file 2 — Reporting Summary [file 41588_2023_1571_MOESM2_ESM.pdf]

Corresponding author(s): Jinfeng Chen, Sha Tang, and Xianmin Diao

Last updated by author(s): Oct 14, 2023

Reporting Summary

Nature Portfolio wishes to improve the reproducibility of the work that we publish. This form provides structure for consistency and transparency in reporting. For further information on Nature Portfolio policies, see our Editorial Policies and the Editorial Policy Checklist.

Statistics

For all statistical analyses, confirm that the following items are present in the figure legend, table legend, main text, or Methods section.

n/a	Confirmed	
☐	☒	The exact sample size (*n*) for each experimental group/condition, given as a discrete number and unit of measurement
☐	☒	A statement on whether measurements were taken from distinct samples or whether the same sample was measured repeatedly
☐	☒	The statistical test(s) used AND whether they are one- or two-sided<br>*Only common tests should be described solely by name; describe more complex techniques in the Methods section.*
☐	☒	A description of all covariates tested
☐	☒	A description of any assumptions or corrections, such as tests of normality and adjustment for multiple comparisons
☐	☒	A full description of the statistical parameters including central tendency (e.g. means) or other basic estimates (e.g. regression coefficient) AND variation (e.g. standard deviation) or associated estimates of uncertainty (e.g. confidence intervals)
☐	☒	For null hypothesis testing, the test statistic (e.g. *F*, *t*, *r*) with confidence intervals, effect sizes, degrees of freedom and *P* value noted<br>*Give P values as exact values whenever suitable.*
☒	☐	For Bayesian analysis, information on the choice of priors and Markov chain Monte Carlo settings
☒	☐	For hierarchical and complex designs, identification of the appropriate level for tests and full reporting of outcomes
☐	☒	Estimates of effect sizes (e.g. Cohen's *d*, Pearson's *r*), indicating how they were calculated

Our web collection on statistics for biologists contains articles on many of the points above.

Software and code

Policy information about availability of computer code

Data collection	1, Seed dimensions including seed width, seed length, and seed weight were measured with SC-G software (Hangzhou Wanshen Detection Technology)<br>2, High-fidelity (HiFi) long reads were collected using the Circular Consensus Sequencing (CCS) analysis application with SMRT Link.
Data analysis	1, The raw reads were filtered by removing low-quality bases and sequencing adaptors with Trimmomatic (v0.39)<br>2, Clean reads were aligned to the Longmi4 genome with BWA-MEM using speedseq (v0.0.2)<br>3, Genomic variations including SNPs and INDELs were performed with GATK UnifiedGenotyper (v3.8)<br>4, Fourfold degenerate synonymous sites (4DTv) were obtained from ANNOVAR (v2020-06-08)<br>5, 4DTv sites were used to construct a maximum likelihood phylogenetic tree using IQ-TREE (v2.1.4-beta)<br>6, Principle component analysis (PCA) of 516 broomcorn millet accessions was conducted by plink (v1.90b6.18)<br>7, Population structure analysis was performed by ADMIXTURE (v1.3.0), fastSTRUCTURE (v1.0), STRUCTURE (v2.3.4), DAPC in adegenet (v2.1.8)<br>8, Linkage disequilibrium was calculated with PopLDDecay (v3.41)<br>9, Artificial selection sweeps during domestication and improvement were detected by combining cross-population composite likelihood ratio test (XP-CLR v1.0)<br>10, Top 5% was used as a threshold for significance. XP-CLR scores were then smoothed using 100-kb windows with 10-kb steps on each chromosome. VCFtools (v0.1.13)<br>11, Overlap among selection sweeps detected by XP-CLR, πwild / πcultivar, Fst were identified with BEDTools (v2.29.1)<br>12, Raw contigs were generated from HiFi long reads using Hifiasm (v0.14.2-r315)<br>13, Contigs were anchored onto chromosomes using RagTag (v2.0.1)<br>14, To evaluate the quality of assembly, we first performed gene completeness analysis with BUSCO (v4.0.6) and repeat completeness analysis

with the LTR assembly index (LAI) using LTR_retriever (v2.9.0)
15, Hi-C reads were manually inspected with Juicebox (v1.11.08)
16, Gene annotation was performed on each assembly using the MAKER2 pipeline (v2.31.11)
17, Repetitive sequences in each de novo assembly were annotated with RepeatModeler (v2.0.1)
18, The non-redundant protein sequences were clustered into gene families using OrthoFinder (v2.5.4)
19, The ratios of Non-synonymous/Synonymous mutation (Ka/Ks) for each gene of the pan-genome were calculated using KaKs_Calculator (v2.0)
20, A reference-based alignment approach named PopASSYsv (https://github.com/yiliao1022/PoPASSYSV) was used to call and genotype structural variations from our population-scale and highly contiguous genome assemblies.
21, Merged deletions and insertions were genotyped across 516 accessions by short-read data using paragraph (v2.3)
22, Gene Ontology (GO) and Pfam annotation of Pop-DEGs were performed with InterProScan (v5.52-86.0)
23, Kyoto Encyclopedia of Genes and Genomes (KEGG) annotation was performed with BlastKOALA (v2.2) and KofamKOALA
24, RNA seq reads were mapped to the Longmi4 genome using subjunc (v2.0.1)
25, Read counts and FPKM values were calculated using featureCounts (v2.0.1)
26, The differential expression of the genes was analyzed using DESeq2 (v1.32.0)
27, Enriched GO terms and KEGG categories were identified with R package ClusterProfiler (v4.2.2)
28, The missing SNP data were imputed with Beagle (v4.1)
29, The association analysis was performed with EMMAX (vbeta-07Mar2010)
30, The haplotypes of protein-coding genes within candidate regions were analyzed with CandiHap (v1.0.1)
31, The geographical location information of the world sampled accessions in this study are generated using ggmap package in R (v4.1.0) and ArcGIS (v10.2, https://www.arcgis.com/) software
32, Statistical analyses and plotting were performed in R (v.4.1.0) using built-in functions and third-part R packages including tidyverse (v1.3.1), ggplot2 (v.3.4.3), ggpubr (v.0.4.0) and agricolae (v1.3-5)
33, Two-tailed Wilcoxon's rank-sum test was used to compare the difference of expression or phenotype between two groups with R built-in functions wilcox.test
34, One-way ANOVA test was used to determine differences among groups. Pairwise comparison was conducted by the least significant difference (LSD) method with Bonferroni correction for multiple comparisons using function LSD.test in third-part R package agricolae (v1.3-5)
35, Pearson's correlation coefficient (R) and P value was calculated with R function cor.test, fitted curves and 95% confidence intervals for linear regression were also calculated

Code availability
All codes or tools used in this study are described in the methods. Codes are available at Zenodo (https://doi.org/10.5281/zenodo.8373683).

For manuscripts utilizing custom algorithms or software that are central to the research but not yet described in published literature, software must be made available to editors and reviewers. We strongly encourage code deposition in a community repository (e.g. GitHub). See the Nature Portfolio guidelines for submitting code & software for further information.

Data

Policy information about availability of data

All manuscripts must include a data availability statement. This statement should provide the following information, where applicable:

- Accession codes, unique identifiers, or web links for publicly available datasets
- A description of any restrictions on data availability
- For clinical datasets or third party data, please ensure that the statement adheres to our policy

The raw sequences of 516 accessions (BioProject accession no. PRJNA603255), PacBio HiFi reads and RNA-Seq data of 32 accessions (BioProject accession no. PRJNA847741), and the Hi-C sequences of BC170 and BC418 (BC170: SRR17710547-50; BC418: SRR17710545-6, SRR17710553-4) have been deposited in the National Center for Biotechnology Information SRA. The assembled pan-genome sequences and gene and transposable element annotations are available at Zenodo (https://doi.org/10.5281/zenodo.6627574). The assembled pan-genome sequences have also been deposited into the NCBI genome database and their accession numbers (JAVRMQ000000000- JAVRNV000000000) were listed in Supplementary Table 3. The phenotype data are available at Zenodo (https://doi.org/10.5281/zenodo.7749727). All study data are included in the main article and Supplementary Materials. All broomcorn millet accessions are available at the National Crop Genebank of China.

Human research participants

Policy information about studies involving human research participants and Sex and Gender in Research.

Reporting on sex and gender	N/A
Population characteristics	N/A
Recruitment	N/A
Ethics oversight	N/A

Note that full information on the approval of the study protocol must also be provided in the manuscript.

Field-specific reporting

Please select the one below that is the best fit for your research. If you are not sure, read the appropriate sections before making your selection.

☒ Life sciences ☐ Behavioural & social sciences ☐ Ecological, evolutionary & environmental sciences

For a reference copy of the document with all sections, see nature.com/documents/nr-reporting-summary-flat.pdf

Life sciences study design

All studies must disclose on these points even when the disclosure is negative.

Sample size	The sample size of resequencing samples (516) were chosen based on accessions available at the National Crop Genebank of China to cover geographic distribution of broomcorn millet. The sample size (32) of pan-genome samples were chosen based on previous pan-genome studies (1-3) to represent population diversity of broomcorn millet.<br><br>Reference:<br>1, Liu, Y. et al. Pan-genome of wild and cultivated soybeans. Cell 182, 162-176 (2020).<br>2, Hufford, M. B. et al. De novo assembly, annotation, and comparative analysis of 26 diverse maize genomes. Science 373, 655-662 (2021).<br>3, Qin, P. et al. Pan-genome analysis of 33 genetically diverse rice accessions reveals hidden genomic variations. Cell 184, 3542-3558 (2021).
Data exclusions	We did not exclude any data from data analyses.
Replication	RNAseq experiments were performed with three biological replicates. Trait evaluation of 516 accessions were performed with three biological replicates per accession at seven sites. Trait evaluation of CRISPR mutants were performed with five biological replicates. All replications were successful and reported in the manuscript.
Randomization	For trait evaluation, we randomly sampled three plants out of 80 plants per accession in the field. For RNA and DNA extractions, we randomly sampled 3 plants out of 10-15 plants in the greenhouse or growth chamber.
Blinding	Blinding is not necessary for sampling accessions for genome sequencing. The samples for genome sequencing of broomcorn millets were selected based on their geographic distribution, phylogenetic tree as we need to cover the geographic distribution and genetic diversity of this species. The investigators were blinded to population structure or origin of accessions when collecting traits in the field.

Reporting for specific materials, systems and methods

We require information from authors about some types of materials, experimental systems and methods used in many studies. Here, indicate whether each material, system or method listed is relevant to your study. If you are not sure if a list item applies to your research, read the appropriate section before selecting a response.

Materials & experimental systems

n/a	Involved in the study	
☒	☐	Antibodies
☒	☐	Eukaryotic cell lines
☒	☐	Palaeontology and archaeology
☒	☐	Animals and other organisms
☒	☐	Clinical data
☒	☐	Dual use research of concern

Methods

n/a	Involved in the study	
☒	☐	ChIP-seq
☒	☐	Flow cytometry
☒	☐	MRI-based neuroimaging
